# The gene-regulatory evolution of the human skeleton

**DOI:** 10.1038/s41586-026-11053-x

**Published:** 2026-09-23

**Authors:** Yizhi Yan, Nadav Mishol, Katharina Lange, Zicong Zhang, Gal Bodek, Aya Kigel, Noam Priel, Nachshon Egyes, Omer Ronen, Itamar Nini, Liat Rotenstreich, Amit Philosoph, Sira Martinez, Silvia Beltramone, Rika Tsujikawa, Adi Rozenblatt, Lucas Esteban Wange, María Torralvo, Guy Hirsh, Yael Elboim, Sergey Viukov, Idan Korenfeld, Mythili Damal Kandadai, Océane Cluzeau, Malka Nissim-Rafinia, Eran Meshorer, Jacob H. Hanna, Evie Vereecke, Assaf Marom, Martin Kuhlwilm, Guillaume Bourque, Tomas Marques-Bonet, Simon Fishilevich, Fumitaka Inoue, David Gokhman

**Affiliations:** 1 Institute for the Advanced Study of Human Biology (WPI-ASHBi), Kyoto University, Kyoto, Japan; 2 Department of Human Genetics, McGill University, Montréal, Quebec Canada; 3 Department of Molecular Genetics, Weizmann Institute of Science, Rehovot, Israel; 4 Department of Anatomy, Rappaport Faculty of Medicine, Technion — Israel Institute of Technology, Haifa, Israel; 5 European Molecular Biology Laboratory (EMBL Barcelona), Barcelona Biomedical Research Park, Barcelona, Spain; 6 Institute of Evolutionary Biology (UPF-CSIC) and Centre for Genomic Regulation (CRG), Barcelona Biomedical Research Park, Barcelona, Spain; 7 Catalan Institution of Research and Advanced Studies (ICREA), Barcelona, Spain; 8 Centro Nacional de Análisis Genómico (CNAG), CRG, Barcelona Institute of Science and Technology, Barcelona, Spain; 9 Institut Català de Paleontologia Miquel Crusafont, Universitat Autònoma de Barcelona, Barcelona, Spain; 10 Neurogenomics Group, Research Programme on Biomedical Informatics (GRIB), Hospital del Mar Medical Research Institute (IMIM), Barcelona, Spain; 11 Department of Development and Regeneration, KU Leuven, Leuven, Belgium; 12 Department of Human Structure and Repair, Faculty of Medicine and Health Sciences, Ghent University, Ghent, Belgium; 13 Department of Genetics, Institute of Life Sciences, Hebrew University of Jerusalem, Jerusalem, Israel; 14 Edmond and Lily Safra Center for Brain Sciences (ELSC), Hebrew University of Jerusalem, Jerusalem, Israel; 15 Department of Evolutionary Anthropology, University of Vienna, Vienna, Austria; 16 Human Evolution and Archaeological Science Network, University of Vienna, Vienna, Austria; 17 Victor Phillip Dahdaleh Institute of Genomic Medicine, McGill University, Montréal, Quebec Canada; 18 Canadian Center for Computational Genomics, McGill University, Montréal, Quebec Canada

**Keywords:** Gene expression profiling, Evolutionary biology, Gene regulation, Evolutionary genetics, Bone

## Abstract

Skeletal modifications were central to human evolution, enabling adaptations for bipedalism, large cranial vaults and childbirth^1^. Despite their importance, the genetic changes that gave rise to the unique human form remain mostly unknown^2^. Here we systematically map the gene-regulatory changes that shaped human skeletal evolution. Using massively parallel reporter assays (MPRAs) in chondrocytes, we assayed 561,410 human-derived substitutions in promoters and enhancers, identifying 15,077 loci with human-specific regulatory activity. We then generated human–ape hybrid cells and differentiated them into osteochondral progenitors. Integrating the hybrid cells with MPRA measurements produced genome-wide atlases of human-specific changes in *cis*-regulatory expression, and the sequence variants that drive them. These atlases reveal an extensive rewiring of the extracellular matrix (ECM), including a marked suppression of glycosaminoglycan (GAG) biosynthesis, leading to an approximately three-to-fourfold reduction in joint GAG content in humans compared with non-human apes. We find that this human-specific shift bears signatures of selection, and is likely to be a key contributor to the exceptional susceptibility of humans to degenerative skeletal diseases^3–5^. Together, our results reveal a coordinated evolutionary remodelling of the human skeletal ECM, and establish a comprehensive framework for dissecting the genetic basis of human skeletal biology.

## Abstract (web summary)

Massively parallel reporter assays and human–ape hybrid skeletal cells are used to study the evolution of human *cis*-regulatory elements (CREs), revealing how differences in CREs have shaped the composition of human skeletal tissue.

## Main

Humans have a unique set of phenotypes that distinguish them from other great apes. The skeleton has had a particularly central role in human evolution, facilitating hallmark human features such as bipedal locomotion, slender bones and large cranial vaults. These skeletal features are well-characterized at the phenotypic level^1^, but our understanding of the genetic changes that propelled them, and the pathologies that emerged with them, is limited^2^.

Phenotypic divergence between closely related species is thought to be driven mainly by gene-regulatory changes^6,7^. Many of these changes occur in *cis*-regulatory elements (CREs), such as promoters and enhancers, which control the spatio-temporal expression of their target genes^8^. *Cis*-regulatory changes are particularly central in skeletal evolution because they are considered major drivers of morphological divergence^8,9^. Therefore, to decipher the genetic basis of human adaptations, it is imperative to investigate human evolution through the lens of *cis*-regulation.

**Fig. 1:**
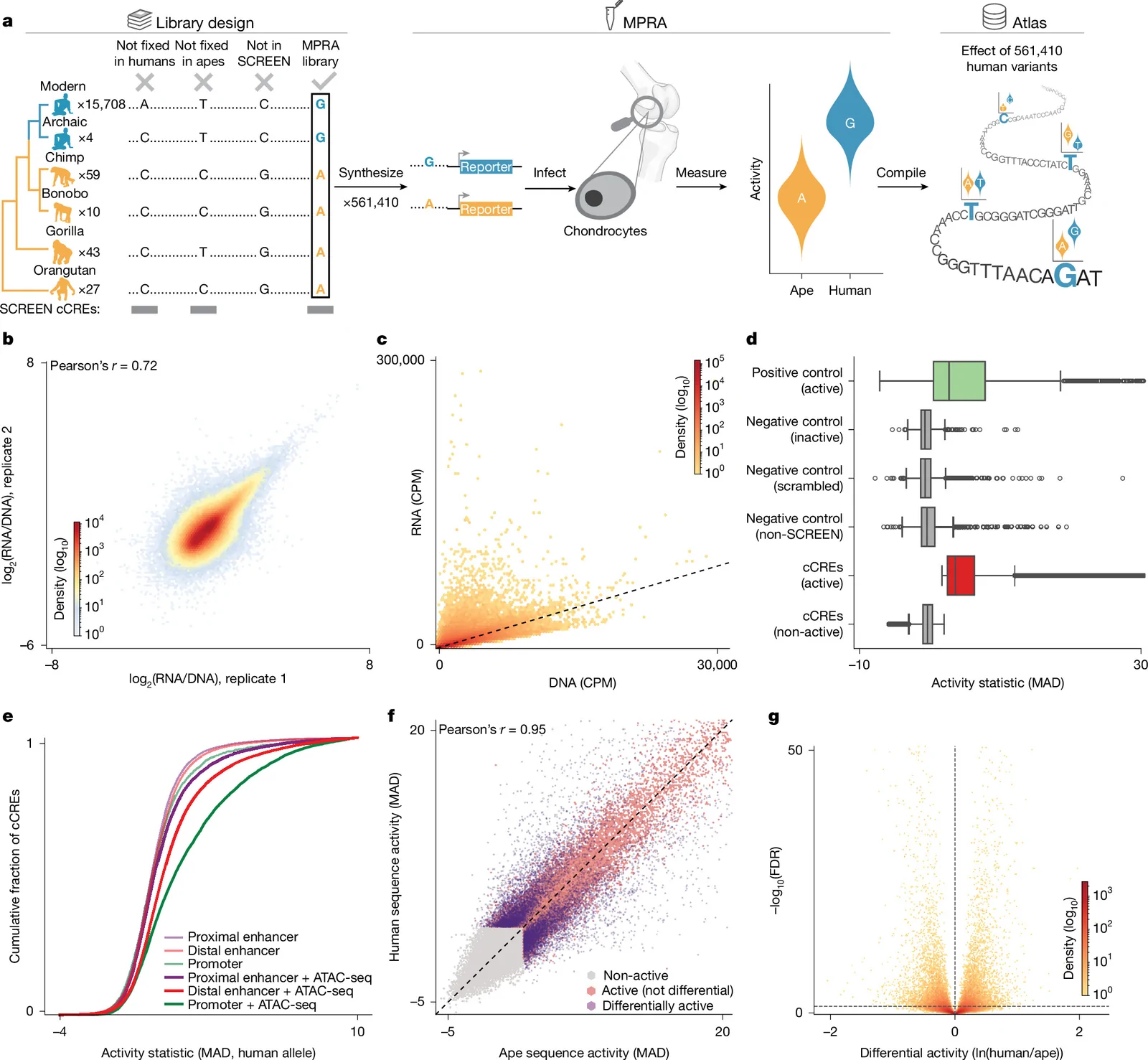
MPRA identifies variants that drive differential expression in human chondrocytes. **a**,A total of 561,410 single-nucleotide substitutions that distinguish humans from other great apes and that fall within cCREs^21^ were assayed by synthesizing the human (derived) and great ape (ancestral) allele of each cCRE, introducing them into human fetal chondrocytes and quantifying their transcriptional effect. This yielded a genome-wide atlas of the functional effects of each regulatory variant that arose and became fixed or nearly fixed in human evolution. **b**, Correlation of cCRE activity across biological replicates. **c**, RNA versus DNA counts per cCRE, normalized to sequencing depth (counts per million; CPM). RNA counts are capped at 300,000. **d**, Box plots showing activity (median absolute deviation; MAD) of positive controls (*n* = 1,404), negative controls (inactive, *n* = 961; scrambled, *n* = 1,051; and non-SCREEN, *n* = 759) and cCRE test sequences (active, *n* = 87,704; non-active, *n* = 557,093). Values are capped at 30. Box plots show the median (centre), second and third quartiles (box boundaries) and the minima and maxima within 1.5× the interquartile range (whiskers). Points beyond these whiskers are plotted individually as outliers. **e**, Empirical cumulative distribution function, showing the overlap of cCREs with chondrocyte active chromatin marks^21^, and open chromatin (ATAC-seq). cCREs with higher activity exhibit greater overlap with active chromatin features. **f**, Scatter plot of human versus great ape alleles of non-active (grey), active but non-differentially active (pink) and differentially active (purple) cCREs. Values are capped at MAD score = 20. **g**, Differential activity volcano plot of human versus great ape alleles for all cCREs with 50 or more DNA reads.

MPRAs are a powerful method to measure the *cis*-regulatory activity of thousands of variants simultaneously^10,11^. In MPRAs, a candidate CRE (cCRE) is cloned upstream of a transcribable DNA barcode, and the RNA abundance of each barcode is used to measure the expression driven by each sequence (Fig. 1a). Thus, MPRAs can be used to characterize variants underlying divergent regulation and to generate a genome-wide atlas of functional variants^10,11^.

We previously used MPRAs to investigate the 14,042 substitutions that separate modern humans from Neanderthals and Denisovans^12^. MPRAs have also been applied to investigate deeper hominin evolution; that is, the divergence between humans and other great apes^10,11^. These studies have made important contributions to our understanding of human divergence, but—with the exception of one report^13^—have focused mostly on neural cell types or on restricted classes of loci (for example, human accelerated regions), which represent a small fraction of evolutionary changes. Of particular interest are fixed human-derived substitutions, which possibly contributed to human-specific phenotypes, and which in some cases might have reached fixation through positive selection. However, their functional effects have not so far been systematically studied. As a result, the overwhelming majority of variants that distinguish humans from their closest relatives remain uncharacterized.

To investigate the regulatory effects of fixed or nearly fixed human-derived substitutions, we designed a library of 732,804 sequences encompassing the human and great ape allele of each cCRE, and assayed their activity using MPRAs. We introduced this library into chondrocytes, a cell type that is central for skeletal development and maintenance. Chondrocytes have a pivotal role in the morphogenesis of most bones by forming the skeletal scaffold during development^1^. They also remain the predominant resident cell type in adult cartilage, where they determine mechanical properties such as viscosity. Using this approach, we identified 15,077 cCREs exhibiting human-specific regulatory activity. To link these sequence-level effects to gene-level regulation, we generated human–chimpanzee and human–gorilla hybrid osteochondral progenitor cells (composite cells^14^), in which the human and non-human ape genomes are exposed to a shared *trans* environment, enabling *cis*-regulatory contributions to be separated from *trans*-regulatory one. Together, these complementary platforms reveal extensive human divergence affecting the ECM, with possible consequences for human skeletal morphology, function and disease.

## Variants modulating human gene activity

Variants that emerged and spread to fixation in the human lineage but that are absent from other great apes represent prime candidates for driving human-specific traits, with some potentially driven to fixation by positive selection. To identify such variants, we examined genomes from 139 non-human great apes^15^ (10 bonobos, 59 chimpanzees, 43 gorillas and 27 orangutans; hereafter referred to as great apes) and identified positions at which the genomes of all great ape individuals differed from the human reference genome (GRCh37). To filter out positions that are variable in humans, we used 15,708 whole-genome sequences from gnomAD v.2.1.1 (ref. ^16^), as well as three high-coverage Neanderthal genomes^17–19^ and a high-coverage Denisovan genome^20^. We took only variants that are 100% fixed across all of these human genomes, which are likely to have emerged before the split between the modern and archaic human lineages. Finally, we filtered the list for variants found within putative regulatory elements, defined by SCREEN v.2 using active chromatin marks across 839 human cell types and tissues^21^ (Methods). Together, this generated a catalogue of 561,410 candidate regulatory variants that distinguish the genus *Homo* (Fig. 1a) and that probably emerged in the period when most hallmark human-derived skeletal phenotypes—including bipedalism, small jaws and prolonged skeletal maturation—appeared in the fossil record^1^.

**Extended Data Fig. 1:**
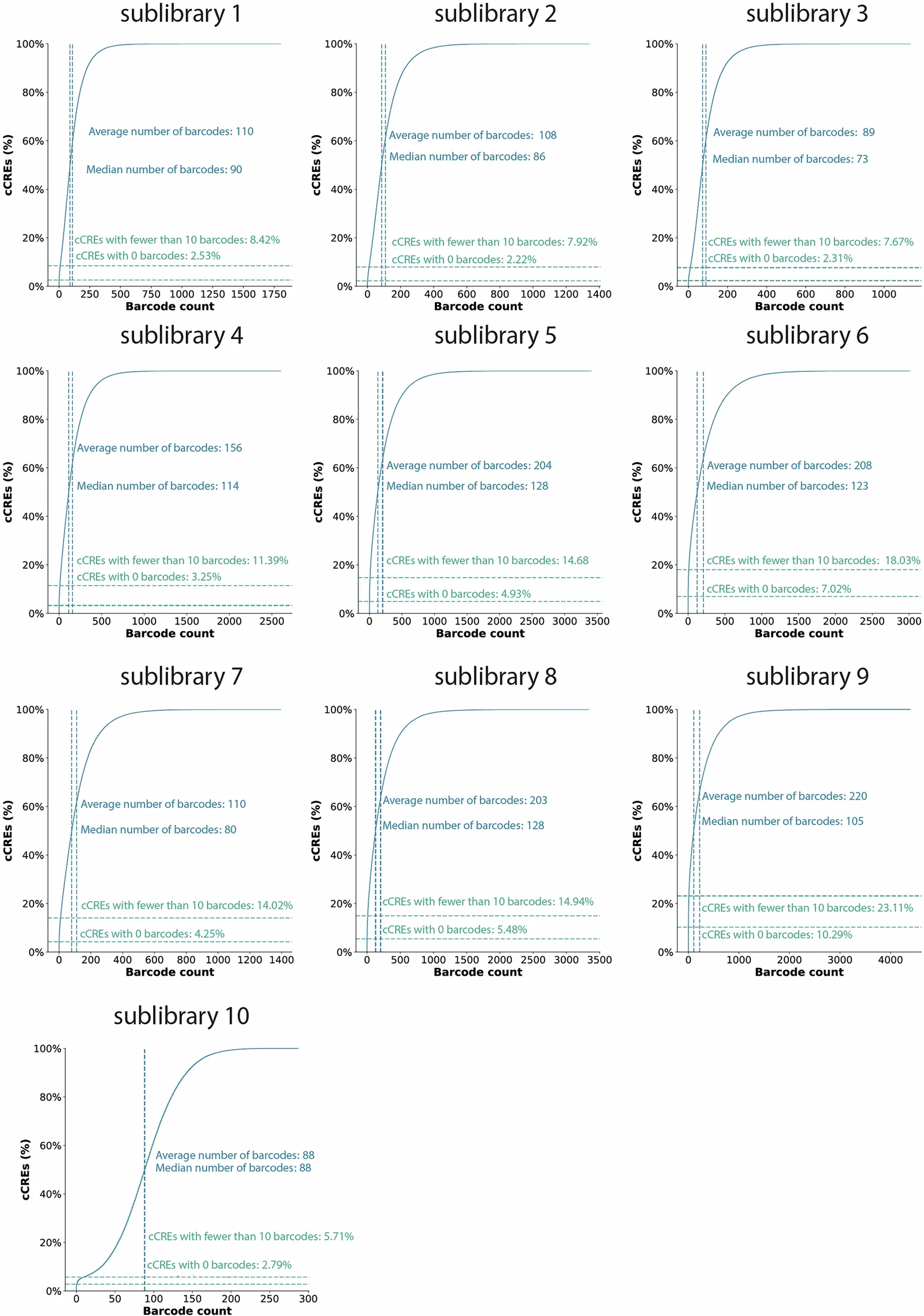
MPRA quality control: cCRE–barcode associations. Empirical cumulative distributions of barcodes per cCRE for each of the ten sublibraries.

To systematically examine the regulatory function of the candidate variants, we performed lentivirus-based MPRAs (lentiMPRAs) using human primary chondrocytes derived from fetal articular cartilage. We synthesized pairs of 270-base pair (bp) sequences centred around each variant, with one sequence for the human-derived allele and the other for the great ape ancestral allele. Variants fewer than 200 bp apart were included together in the same sequence. Overall, the library comprised 561,410 human-derived variants in 366,402 sequence pairs (732,804 total sequences). Each sequence was linked to a median of 73 unique barcodes, enabling independent molecular replication and robust estimates of activity across barcodes (Extended Data Fig. 1 and Supplementary Table 1). In addition, we included 1,500 positive and 3,000 negative controls for activity, as well as 1,307 sequence pairs as positive controls for differential activity. We then infected the library into chondrocytes in triplicate, and sequenced the RNA and DNA barcodes to quantify the relative barcode expression. Finally, we performed an assay for transposase-accessible chromatin with sequencing (ATAC-seq) and cleavage under targets and tagmentation (CUT&Tag) for acetylation of lysine 27 on histone H3 (H3K27ac) on these cells to identify endogenously accessible cCREs (Methods).

**Extended Data Fig. 2:**
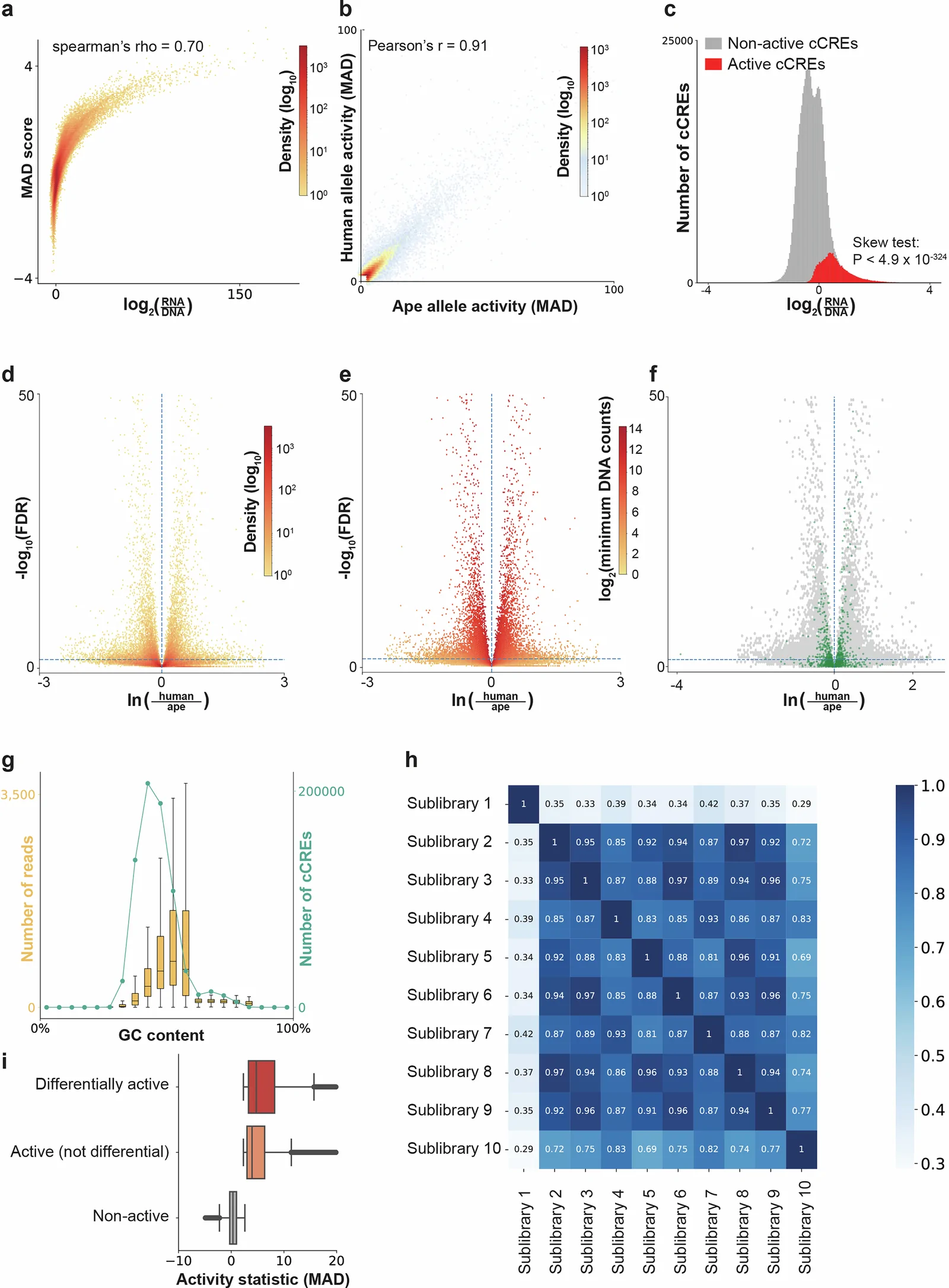
MPRA quality control for activity and differential activity. **a**, Correlation between RNA-to-DNA ratio and MAD score. Hexbin plot; *x* axis capped at 200. **b**, Correlation of activity (MAD score) between allelic pairs for active cCRE with ≥50 DNA counts per allele. Values capped at 100 in both axes. **c**, Histogram of RNA-to-DNA ratio for all cCREs. Active cCREs (FDR < 0.05) are in red. Skewness *P* value was calculated using a two-sided D’Agostino’s skewness test. **d**, Hexbin volcano plot of differential activity, coloured by cCRE density. *Y* axis capped at 50. **e**, Hexbin volcano plot of differential activity, coloured by minimum DNA counts per cCRE. *Y* axis capped at 50. **f**, Hexbin volcano plot of differential activity for all test cCREs (grey), overlayed with scatter plot of positive controls for differential activity (green). *Y* axis capped at 50.** g**, The number of reads per cCRE and number of cCREs at various GC content levels. **h**, Pearson’s r between positive controls across sublibraries. **i**, Activity values of test cCREs, separated into differentially active cCREs (n = 18,434), active cCREs (n = 66,270), and non-active cCREs (n = 557,093). Values capped at 20. Box plots show the median (centre), second and third quartiles (box boundaries) and the minima and maxima within 1.5 times the interquartile range (whiskers). Points beyond these whiskers are plotted individually as outliers in **i**.

To identify sequences that can drive expression, we used MPRAnalyze^22^, which jointly models DNA and RNA counts to estimate transcriptional activity. A sequence pair was classified as active if at least one of its alleles drove significant expression (false discovery rate (FDR) ≤ 0.05; Methods). In total, 65,576 (17.90%) sequence pairs showed significant activity in at least one allele (Extended Data Fig. 2a–c and Supplementary Table 2).

**Extended Data Fig. 3:**
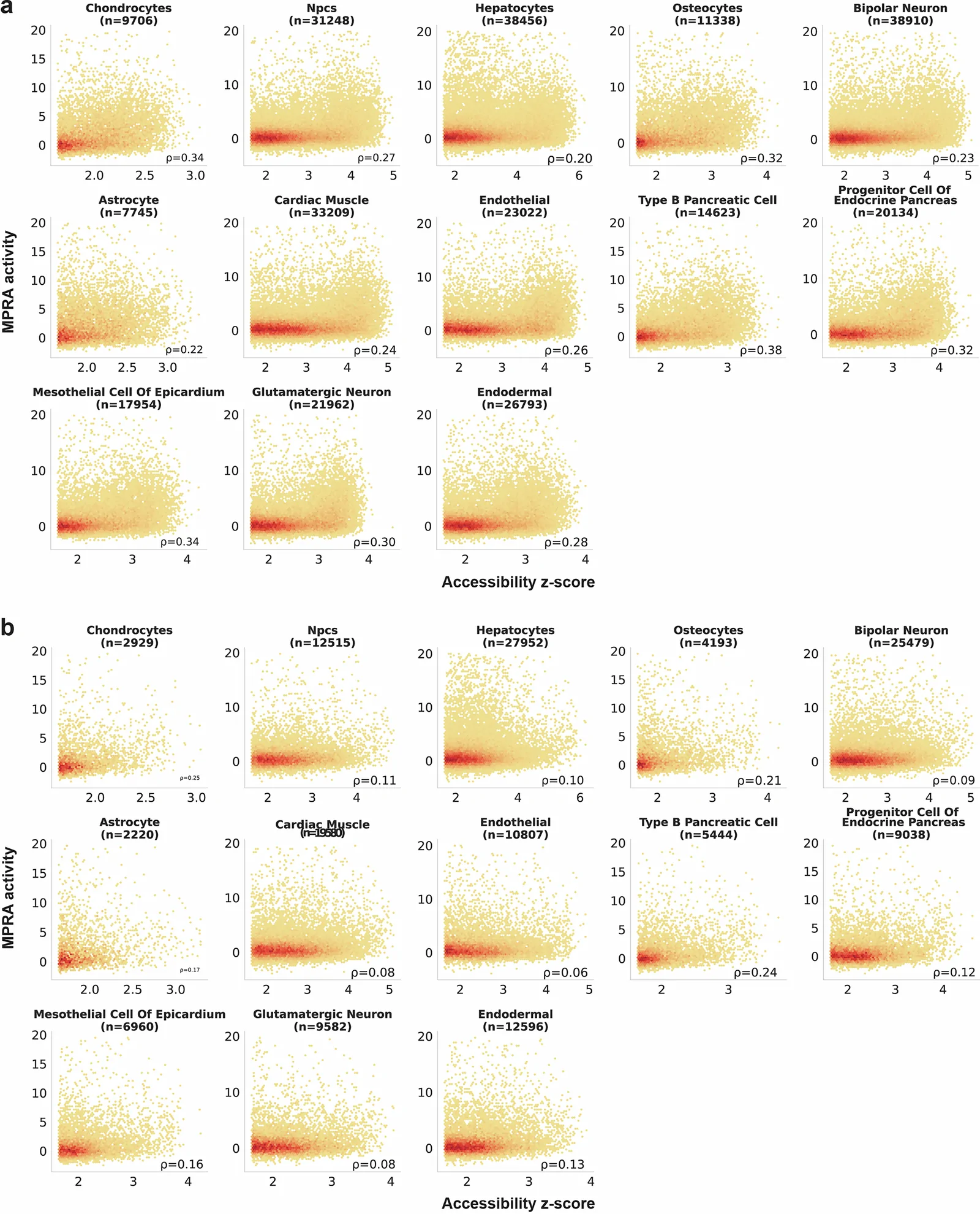
Concordance between MPRA activity and open-chromatin marks across cell types. **a**, Correlation of activity with DNase I cCRE *z*-scores, for chondrocytes and comparable cell types. **b**, Same analyses restricted to enhancer-type cCREs. Y axes were capped at 20 for visualization.

We confirmed that the MPRA results capture true regulatory activity by following the steps described in our MPRA quality-control guidelines^23^. We tested the following key metrics: (i) DNA complexity, (ii) RNA complexity, (iii) reproducibility, (iv) dynamic range of activity and (v) concordance with endogenous signals, and found that the experiment showed high scores across all five metrics (Fig. 1b–e, Extended Data Figs. 2 and 3 and Supplementary Table 3; see Supplementary Information for further details, including further strengths and limitations of MPRAs).

**Extended Data Fig. 4:**
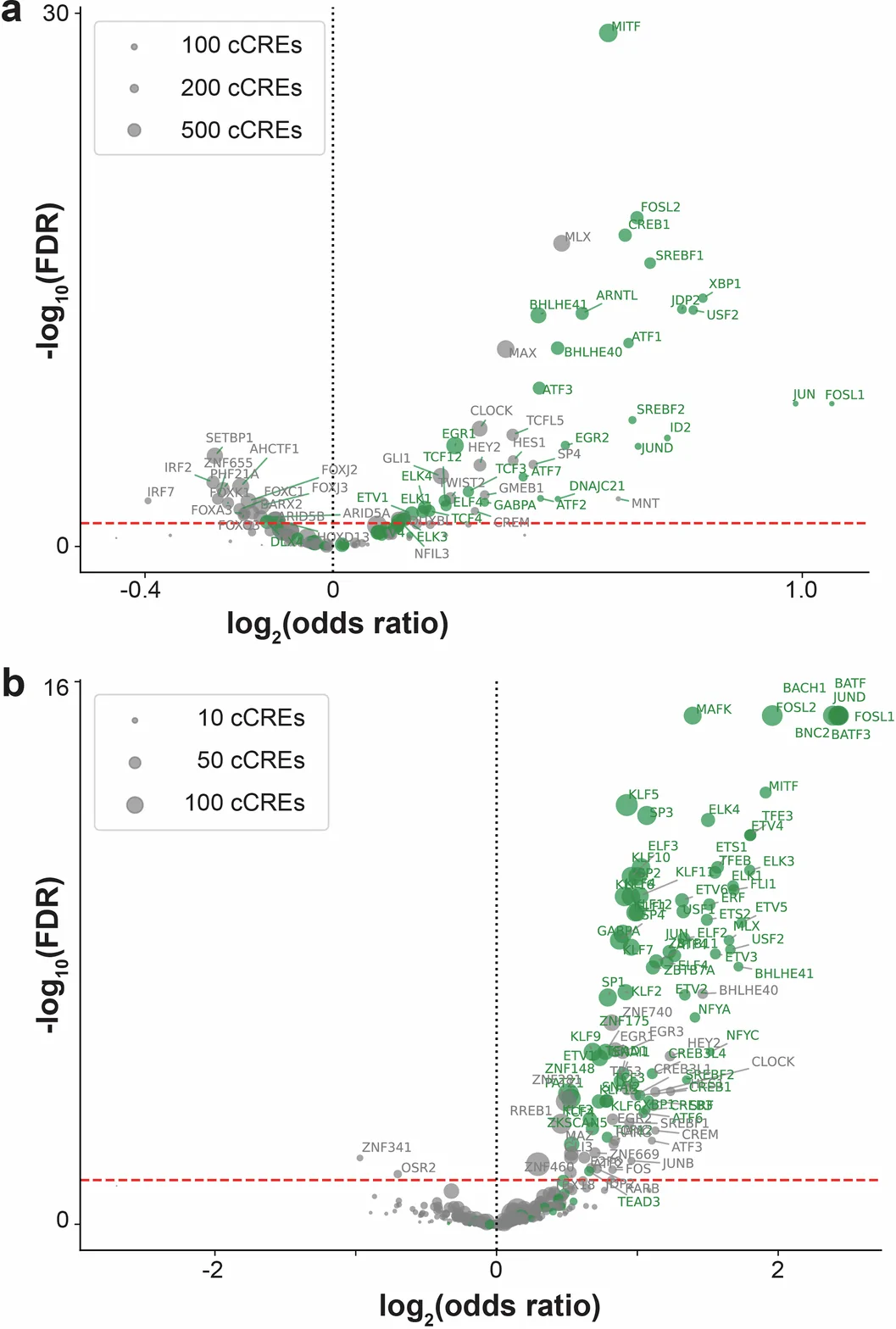
Enriched TFs in differentially active cCREs. **a**,**b**, Volcano plots of predicted TF binding within differentially active cCREs compared to active cCREs. Binding values inferred from PBM data (**a**) and FIMO (**b**). Green indicates significant Pearson’s correlations between TF differential binding and MPRA activity (FDR < 0.05). *X* axis: odds ratio of TF binding in differentially active versus active cCREs. *Y* axis: Fisher’s exact −log_10_ FDR-adjusted *P* values, capped at 30.

To identify variants that alter human gene-regulatory activity, we computed the fold change between the human (derived) and great ape (ancestral) allele using MPRAnalyze^22^. Although activity was highly correlated between the two alleles (Pearson’s *r* = 0.95, *P* < 2.0 × 10^−300^; Fig. 1f and Extended Data Fig. 2b), 15,077 (23.0%) of the active cCREs showed significant differential activity (FDR ≤ 0.05; Fig. 1g, Extended Data Figs. 2d–f and 4, Supplementary Tables 4–7, Supplementary Information and Methods). Here, too, we applied our quality-control pipeline^23^ to assess the robustness of differential activity. Overall, the data showed strong performance, including high reproducibility, a broad dynamic range and concordance with endogenous regulatory signals (Fig. 1b–e and Extended Data Fig. 2).

## Hybrid cells reveal regulatory evolution

**Fig. 2:**
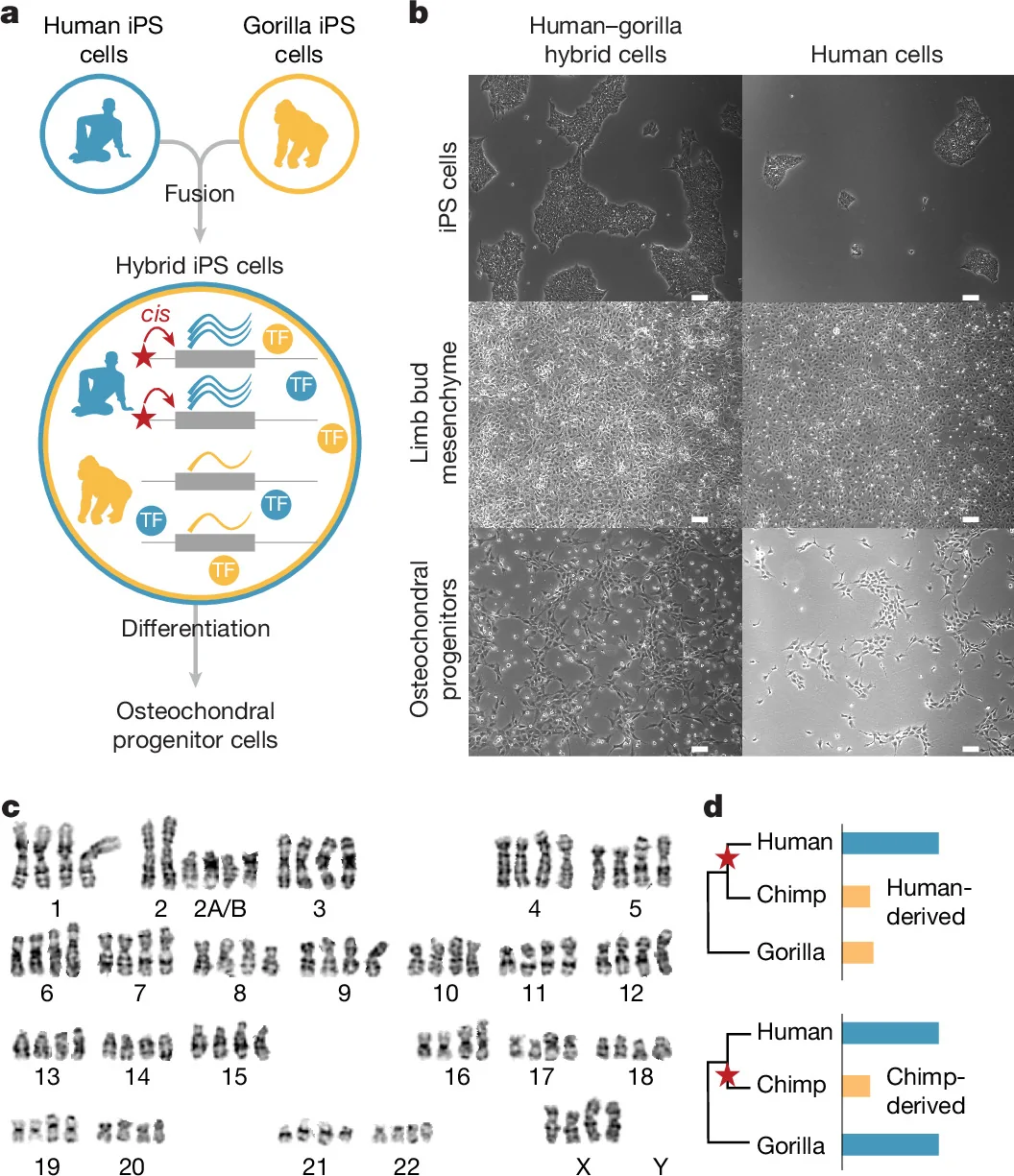
Human–ape hybrid cells reveal human-specific changes in gene expression. **a**, Scheme of the fusion of human and gorilla cells. In hybrid cells, alleles share a *trans* environment, and thus expression changes are the result of *cis*-regulatory divergence. **b**, Phase contrast images of limb osteochondral derivation from human–gorilla hybrid iPS cells and positive control human iPS cells. Differentiation of three human–chimp and two human–gorilla hybrid cells into osteochondral progenitors was done in triplicate. Scale bars, 100 μm. **c**, Karyotype of a tetraploid human–gorilla hybrid cell line (HG2). **d**, Schematic of parsimony-based polarization of *cis*-regulatory gene expression changes. Stars indicate the lineage in which the expression change is likely to have arisen.

Gene expression is shaped not only by single-nucleotide substitutions, but also by other classes of *cis*-regulatory variation, such as indels and structural variants, which were not assayed in our MPRA. To capture *cis*-regulatory divergence irrespective of the underlying variant class, we used allotetraploid composite cell lines, commonly termed interspecies hybrid cells^14^. Unlike hybrid organisms produced through sexual reproduction, these hybrid cells are generated by experimentally fusing cultured cells from two species^24–26^. In such hybrids, alleles from two species share a nucleus, thus experiencing the same *trans*-regulatory environment. Consequently, any difference in expression between the alleles of the two species must be due to *cis*-acting divergence^8^. Moreover, the shared nucleus provides a well-controlled setting, minimizing non-genetic factors such as environmental and batch effects, which often confound interspecific comparisons (Fig. 2a). Of note, unlike aneuploid cells, in which relative gene dosage is distorted, fully tetraploid hybrids of closely related species preserve karyotypic integrity, mostly maintain the balanced gene dosage of diploid cells and are stable in vitro^24–30^ (for further strengths and limitations of the hybrid platform, see Supplementary Information). These properties make hybrid cells a powerful tool for dissecting *cis*-regulatory evolution.

**Extended Data Fig. 5:**
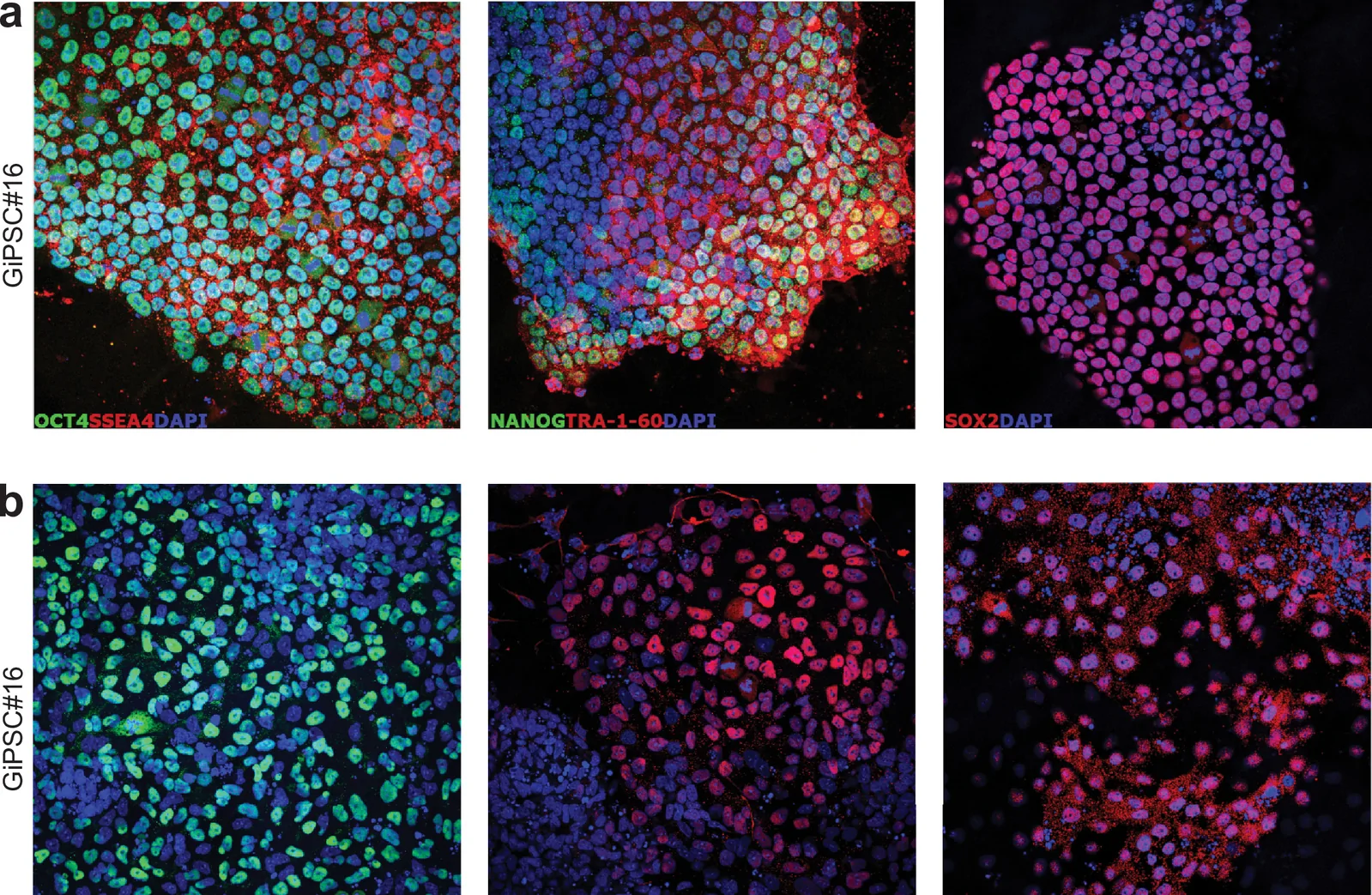
Validation of iPS cells derived from gorilla fibroblasts. **a**, Immunocytochemical characterization of gorilla iPS cell line GiPSC16 showing expression of pluripotency-associated markers OCT4 and SSEA4 (left), NANOG and TRA-1-60 (centre), and SOX2 (right) using the StemLight Pluripotency IF Antibody Sampler Kit (Cell Signaling Technology, 9656). **b**, Immunocytochemical analysis of differentiated derivatives from GiPSC16, showing expression of markers representative of the three germ layers: SOX17 (endoderm, left), OTX2 (ectoderm, middle) and BRACHYURY (mesoderm, right) using the Human Pluripotent Stem Cell Functional Identification Kit (R&D Systems, SC027B). Nuclei were counterstained with DAPI. Scale bars, 20 μm.

**Extended Data Fig. 6:**
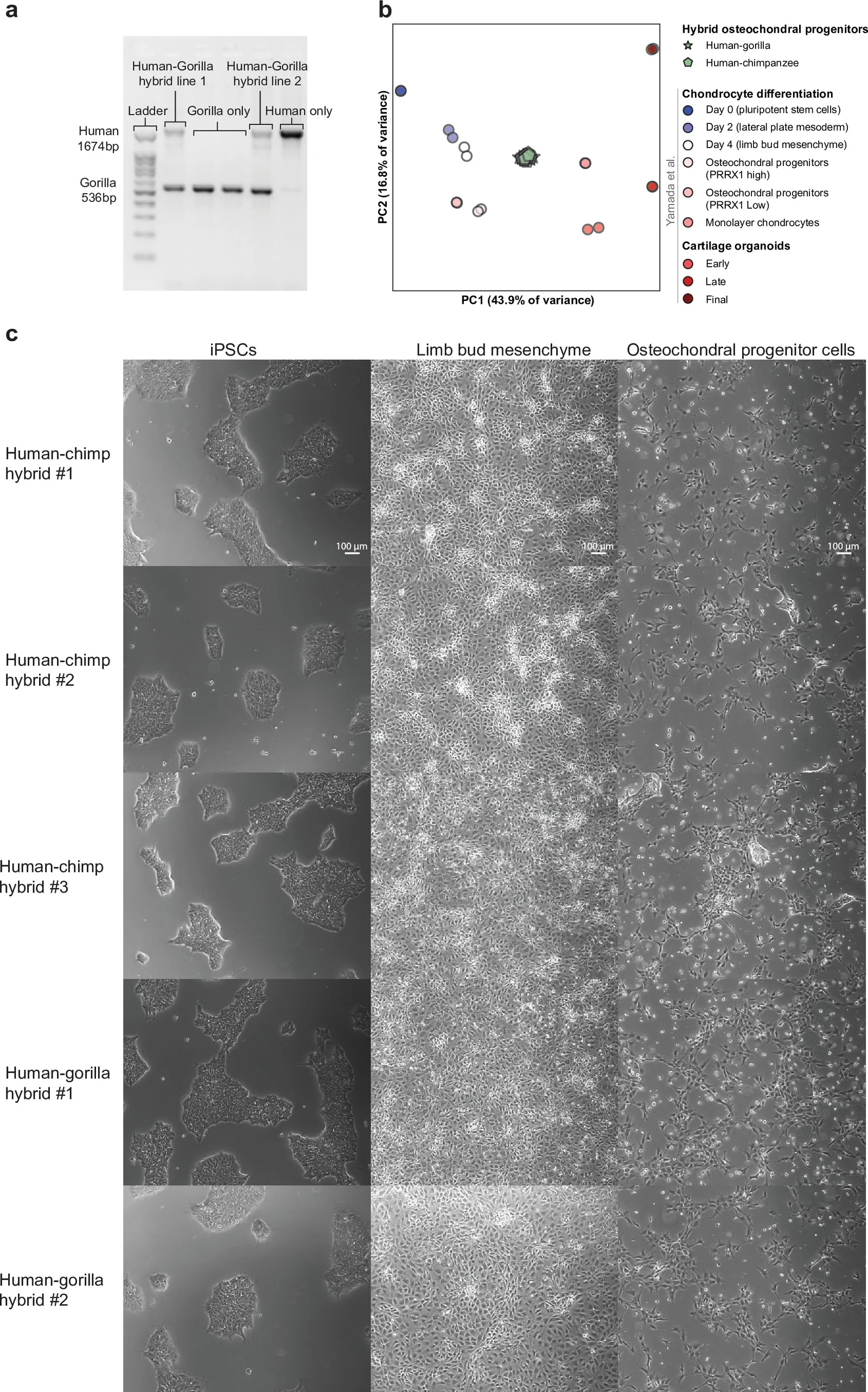
Validation and differentiation of human–ape hybrid cells. **a**, PCR was performed using a mixed set of allele-specific (amplification refractory mutation system) primers for human (1,674 bp) and gorilla (536 bp) amplicons. Two cell lines, denoted as human–gorilla hybrid 1 (HG1) and human–gorilla hybrid 2 (HG2) exhibited both human- and gorilla-specific bands, whereas the others displayed a single species-specific band. **b**, PCA plot of gene expression from cells at different stages of chondrogenic differentiation from Yamada et al. ^123^, and of human–ape hybrid cells. **c**, Differentiation of human-chimpanzee and human–gorilla hybrid iPS cells into osteochondral progenitor cells. Differentiation of three human–chimp and two human–gorilla hybrid cells into osteochondral progenitors was done in triplicate.

To investigate changes in *cis*-regulatory expression in the human skeleton, we established an experimental platform comprising human–gorilla and human–chimpanzee hybrid skeletal cells. To achieve this, we first fused human and gorilla induced pluripotent stem (iPS) cells (Extended Data Fig. 5) using our hybridization protocol^24,25^ to generate two human–gorilla hybrid (allotetraploid) pluripotent stem cell lines (Fig. 2b and Methods). We validated their hybrid identity using karyotyping (Fig. 2c) and PCR (Extended Data Fig. 6a). Then, we differentiated the human–gorilla hybrid cells, along with our previously generated human–chimpanzee hybrid cells^24,25^, into limb osteochondral progenitor cells (Fig. 2b and Extended Data Fig. 6b,c and Methods). Together, these human–ape hybrid platforms allowed us to identify human-specific *cis*-regulatory changes in skeletal gene expression.

Because differences in gene expression between humans and chimpanzees could stem from changes in either lineage, we used the human–chimpanzee hybrid osteochondral progenitor cells to identify differential expression between humans and chimpanzees, and then used the human–gorilla hybrid osteochondral progenitor cells to polarize the lineage on which these changes emerged (Fig. 2d and Methods). Overall, we identified 4,152 chimpanzee-specific and 4,463 human-specific changes in *cis*-regulatory expression (Supplementary Table 8).

Hybrid cells and MPRAs enable regulatory divergence to be examined at two complementary levels: hybrid cells reveal the differences in *cis*-regulatory expression between the species, whereas MPRAs identify the candidate variants that drive those differences. To integrate these datasets into a gene-centric resource, we linked each cCRE to its probable target gene(s) by following the strategy implemented in our GeneHancer^31^ and our modern-human-specific MPRA^12^ frameworks. We used a combination of linear and three-dimensional genomic proximity, statistical associations (expression quantitative trait loci; eQTLs), overlap with promoters and co-variation between gene expression and enhancer RNA (eRNA) levels (Supplementary Table 9 and Methods). Through these complementary regulatory layers, we studied the genes, pathways and functions whose regulation diverged during human skeletal evolution.

## GAG metabolism is an evolutionary hotspot

**Fig. 3:**
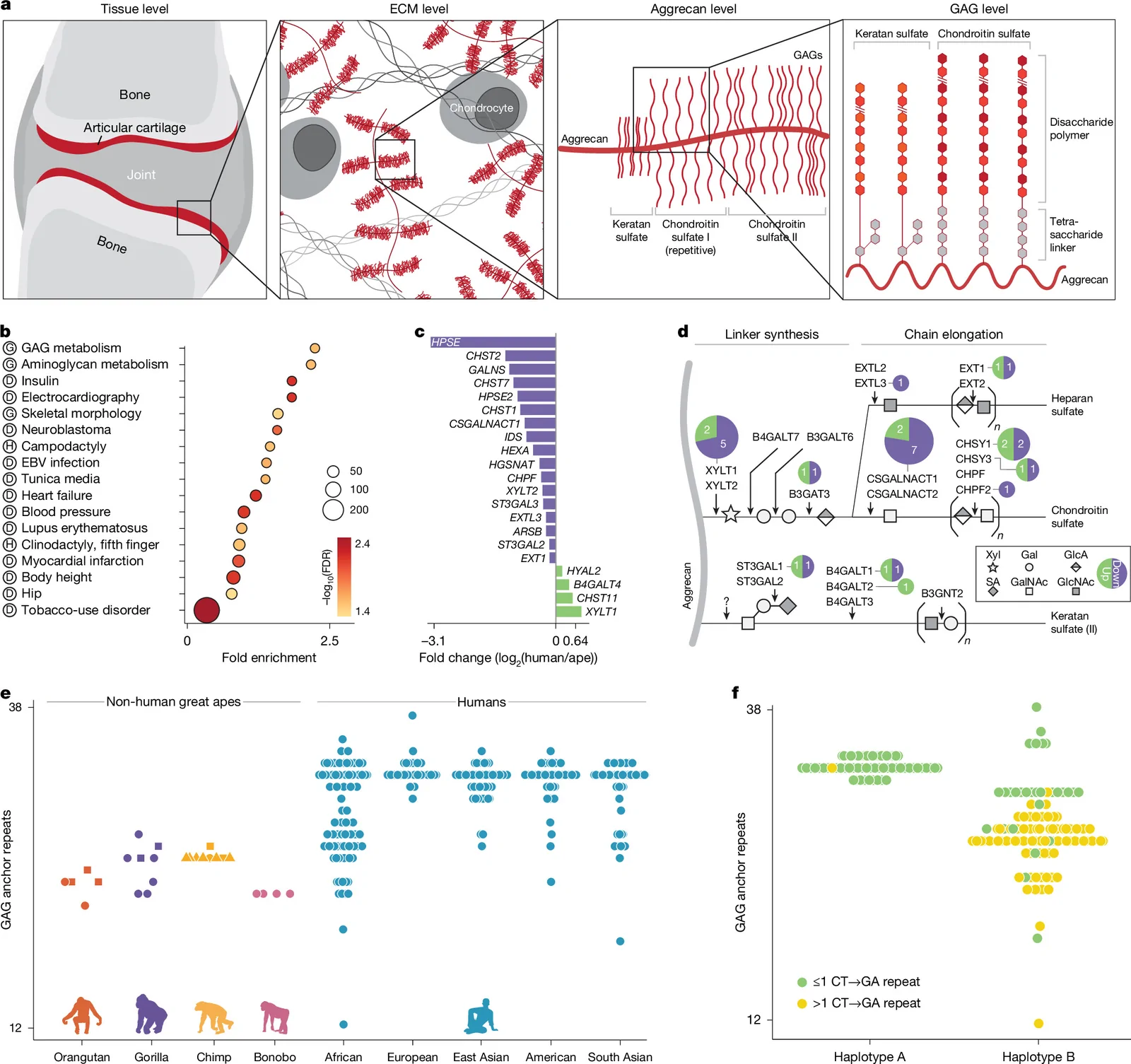
Human divergence in the skeletal ECM. **a**, Schematic of GAGs across biological scales, from the tissue level to the molecular level. At the molecular level, keratan sulfate and chondroitin sulfate chains are synthesized as repeating disaccharide polymers and are attached via a linker region to aggrecan. At the aggrecan level, multiple GAG chains decorate the aggrecan core protein, forming a highly charged, hydrated macromolecule. At the ECM level, aggrecan assemblies are embedded in the cartilage matrix that surrounds chondrocytes. At the tissue level, these ECM structures collectively give rise to the mechanical properties and development of articular cartilage. **b**, Top functional enrichments of MPRA differentially active cCREs using GO^33^ (circled G), HPO^34^ (circled H) and the GAD^35^ (circled D). Circle size indicates the number of genes. EBV, Epstein–Barr virus. **c**, Human-derived differential expression in GAG-related genes, observed in human–ape hybrid osteochondral progenitor cells. Only significantly differentially expressed genes are shown. **d**, GAG linker and chain elongation pathway (adapted from KEGG^38^), overlaid with the number and direction of MPRA differentially active cCREs per gene. Gal, galactose; GalNAc, *N*-acetylgalactosamine; GlcA, glucuronic acid; GlcNAc, *N*-acetylglucosamine; SA, sialic acid; Xyl, xylose. **e**, Human-derived expansion of GAG anchor repeats in ACAN. Shapes denote subspecies (Supplementary Table 13). **f**, Expectation-maximization inference identifies two distributions of repeat numbers. These distributions are also distinguished by the nucleotide composition of their repeats. For example, individuals with a low repeat number tend to have many repeats containing a CT>GA variant at position 30–31 of the repeat.

To investigate the biological functions affected by human regulatory divergence, we used four platforms: (i) ingenuity pathway analysis (IPA)^32^; (ii) Gene Ontology (GO)^33^; (iii) the Human Phenotype Ontology (HPO) database^34^; and (iv) the Genetic Association Database (GAD)^35^. First, in each database, we tested for enrichment in genes associated with MPRA differentially active cCREs compared with in genes associated with other active cCREs (Supplementary Table 10). We found that the most significant enrichment was observed in the GAG pathway. GAGs are carbohydrate chains that decorate ECM- and cell-associated proteoglycans. They are highly abundant in cartilage, comprising 15–30% of its dry weight, second only to collagen^36^. These molecules have particularly central roles in skeletal morphogenesis, growth factor signalling and cartilage maintenance^37^. In GO, we observed an enrichment of GAG metabolic process (2.2×, FDR = 0.03), aminoglycan metabolic process (2.1×, FDR = 0.03) and skeletal system morphogenesis (1.8×, FDR = 0.04) (Fig. 3a,b and Supplementary Table 11). Similarly, we found that in IPA^32^, which infers activation and inhibition, the most significantly downregulated process was GAG metabolism (FDR = 4.0 × 10^−6^; Supplementary Table 11).

Next, we used the osteochondral progenitor hybrid cells to examine gene expression in the GAG biosynthesis pathway (Supplementary Table 12). Similarly to the MPRA results, a downregulation of GAG genes was observed. Pathway analysis using IPA^32^ indicated a downregulation of GAG biosynthesis (FDR = 2.6 × 10^−3^, right-tailed Fisher’s exact test; Supplementary Table 12). This downregulation is further reflected in the observations that (i) most human-derived differentially expressed GAG genes were downregulated (17 out of 21; Fig. 3c); (ii) the magnitude of differential expression of downregulated genes was, on average, nearly twice that of upregulated genes (1.9×; *P* = 0.04, *t*-test; Fig. 3c); and (iii) relative to other human-derived differentially expressed genes, GAG genes tended to be more downregulated (*P* = 2.6 × 10^−3^, *t*-test; Supplementary Table 8). Overall, the integration of MPRA and human–ape hybrid data suggests that the GAG biosynthesis pathway underwent particularly extensive downregulation in human evolution.

To further investigate the evolutionary forces acting on the GAG pathway, we applied a test for lineage-specific selection that compared cCREs associated with GAG genes to other cCREs genome-wide. This test integrates the architecture of cCRE–gene links with the number and magnitude of up- and downregulatory effects to estimate whether their cumulative effect leads to more extreme regulatory shifts than would be expected under a random model (Methods). To achieve higher resolution within GAG biosynthesis, we used KEGG^38^, which partitions this pathway into four components: (a) biosynthesis of chondroitin sulfate; (b) biosynthesis of heparan sulfate; (c) biosynthesis of keratan sulfate; and (d) GAG degradation. Applying the test to the cCREs associated with these GAG pathway components, we detected a pronounced bias towards downregulation in three of these components: chondroitin sulfate biosynthesis (FDR = 0.01, permutation test), heparan sulfate biosynthesis (FDR = 0.01) and GAG degradation (FDR = 0.01).

Substantial downregulation was also observable at the level of specific genes (Fig. 3d), particularly in *CSGALNACT1*, which initiates the key step of chondroitin sulfate chain elongation (Fig. 3d). In the MPRA, *CSGALNACT1* is associated with nine differentially active cCREs, seven of which are downregulating (Fig. 3d and Supplementary Tables 4 and 9). The two most differentially regulated cCREs, both downregulatory, exhibit strong enhancer chromatin marks and multiple signatures of divergence. For instance, the sequence at chromosome (chr.) 8: 19535884–19536153 lies within the first intron of *CSGALNACT1* and is also linked to the gene by overlapping eQTLs and eRNA signal. This cCRE overlaps with ATAC-seq peaks in various bone and cartilage samples, including lumbar and thoracic vertebrae, hand and foot bones^39^ and chondrocytes (Methods). The human allele carries a dinucleotide change (CG>TA; chr. 8: 19536018–19536019) that is predicted to disrupt the binding of two transcription factors (TFs) expressed in chondrocytes: HES1 and BHLHE41 (ref. ^40^). Consistent with this, the human allele shows 34% lower MPRA activity than the ancestral allele does (FDR = 2.1 × 10^−9^). In agreement with the MPRA results, in the human–ape hybrid osteochondral progenitor cells, the human *CSGALNACT1* allele is significantly downregulated relative to both the chimpanzee (−42% FDR = 2.4 × 10^−4^; Fig. 3c) and the gorilla (−34% FDR = 0.012; Supplementary Table 8) counterparts. A similar pattern is observed in in-vitro-differentiated osteogenic cells^41^. Together, this suggests that *CSGALNACT1* became downregulated in the human lineage.

Notably, both *CSGALNACT1* and *ACAN*—which encodes the GAG scaffold proteoglycan aggrecan (Fig. 3a)—exhibit marked divergence in more recent human skeletal evolution; their promoters became hypermethylated in modern humans after their split from Neanderthals and Denisovans^42,43^. Notably, *ACAN* is one of the hotspots of skeletal methylation changes that distinguish modern humans from their closest extinct relatives^43^.

Given its role as the main scaffold for GAG anchoring in cartilage, and one of the most heavily GAG-decorated proteins in the body, we further delved into the evolutionary dynamics of *ACAN*. We focused on exon 12, which encodes the GAG anchor sites. This exon encodes two distinct groups of chondroitin sulfate anchor sites: (i) a 1,995-bp region (chr. 15: 89400653–89402648) encoding 53 anchor sites (domain CS-II), and (ii) a series of 57-nucleotide repeats, each specifying two anchor sites (chr. 15: 89398370–89400652) (domain CS-I). Phenotypically, variation in *ACAN* GAG anchor repeats has been shown to have a probable causal effect on human height^44^, stronger than any other polymorphism in the genome^45^. This variation in repeats has also been associated with a risk of disc degeneration, disc herniation and knee and hand osteoarthritis^46^.

**Extended Data Fig. 7:**
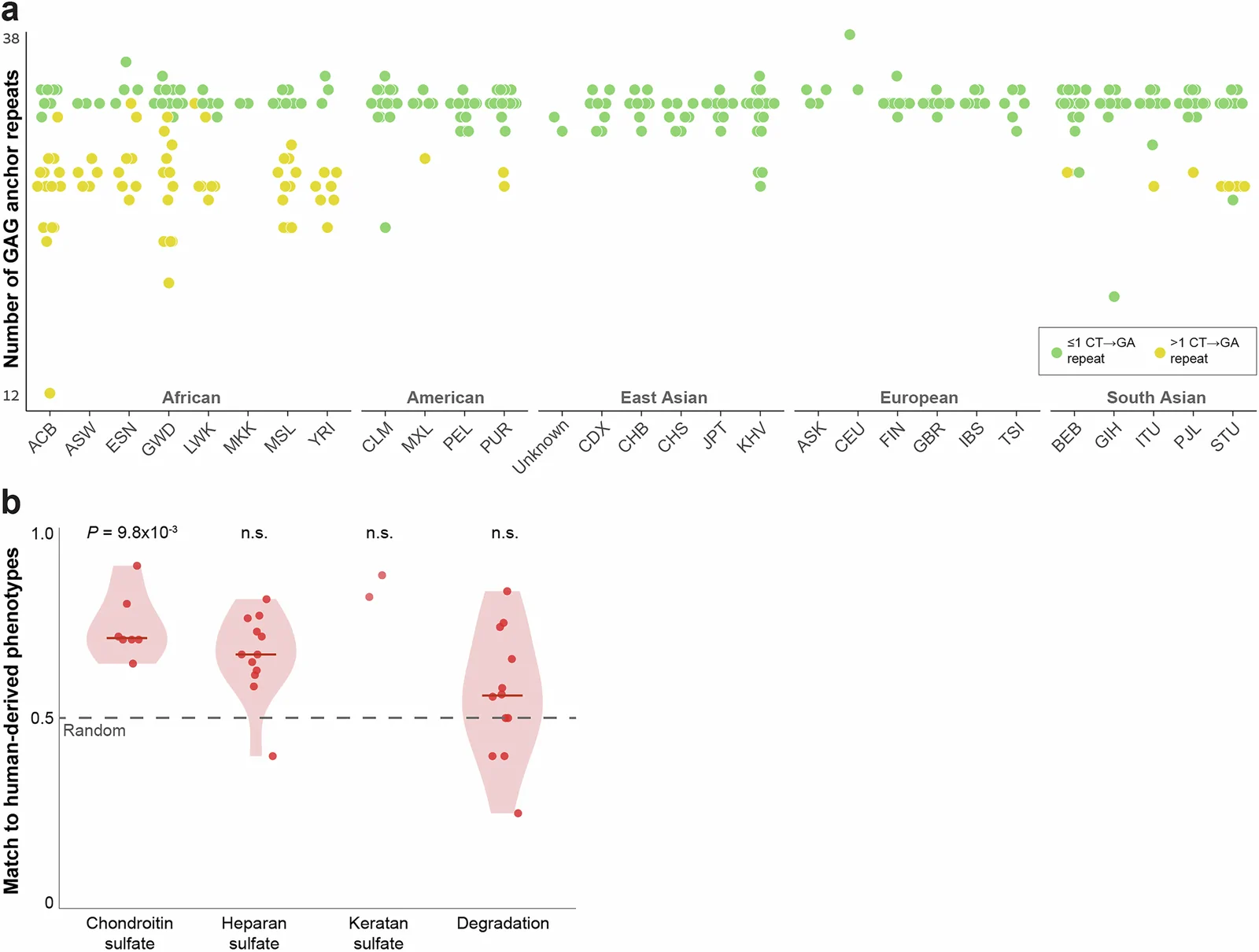
GAG and ACAN divergence. **a**, The number of ACAN GAG anchor repeats across human populations. Humans exhibit two distinct haplogroups, differing in both repeat number and the nucleotide composition of repeats. Repeats with a GA at position 30–31 are common in the low-copy-number allele, but rare otherwise. See Supplementary Table 14 for full details on populations. **b**, Violin plots showing the fraction of HPO loss-of-function phenotypes whose direction matches the direction of phenotypic divergence between humans and chimpanzees. Each point shows the fraction of matching traits per GAG gene. Horizontal red lines indicate the median. *P* values were obtained from a one-sided t-test, values were not adjusted for multiple testing.

To study the evolutionary forces that shape *ACAN* repeats, we analysed long-read sequences of 26 non-human great ape (Supplementary Table 13) and 316 human (Supplementary Table 14) haplotypes, allowing us to compare between-species and within-species variation (Methods). Extending findings from a previous study that analysed a single individual per species^47^, we found that the number of anchor points is highly conserved among non-human great ape lineages, whereas humans exhibit a marked increase, averaging 6 additional repeats corresponding to 12 GAG anchor sites, 342 bp and 114 amino acids (*P* = 3.6 × 10^−15^, Mann–Whitney *U*-test; Fig. 3e). Notably, this elongation appears to have happened in two pulses; using an expectation-maximization algorithm (Methods), we identified two divergent human haplogroups, differing in both repeat number and the nucleotide composition of their repeats (Fig. 3f). The low-copy-number haplogroup is found mainly in Africa, whereas the high-copy-number haplogroup is common across all human populations (Extended Data Fig. 7a). The conservation of *ACAN* repeat number across millions of years of great ape evolution, contrasted with two human-specific pulses of repeat expansion that elongated the coding sequence by hundreds of base pairs, as well as some of the most extensive methylation changes in modern humans^42,43^, points to accelerated evolution of *ACAN* in the human lineage.

## GAG loss shaped human joint evolution

**Fig. 4:**
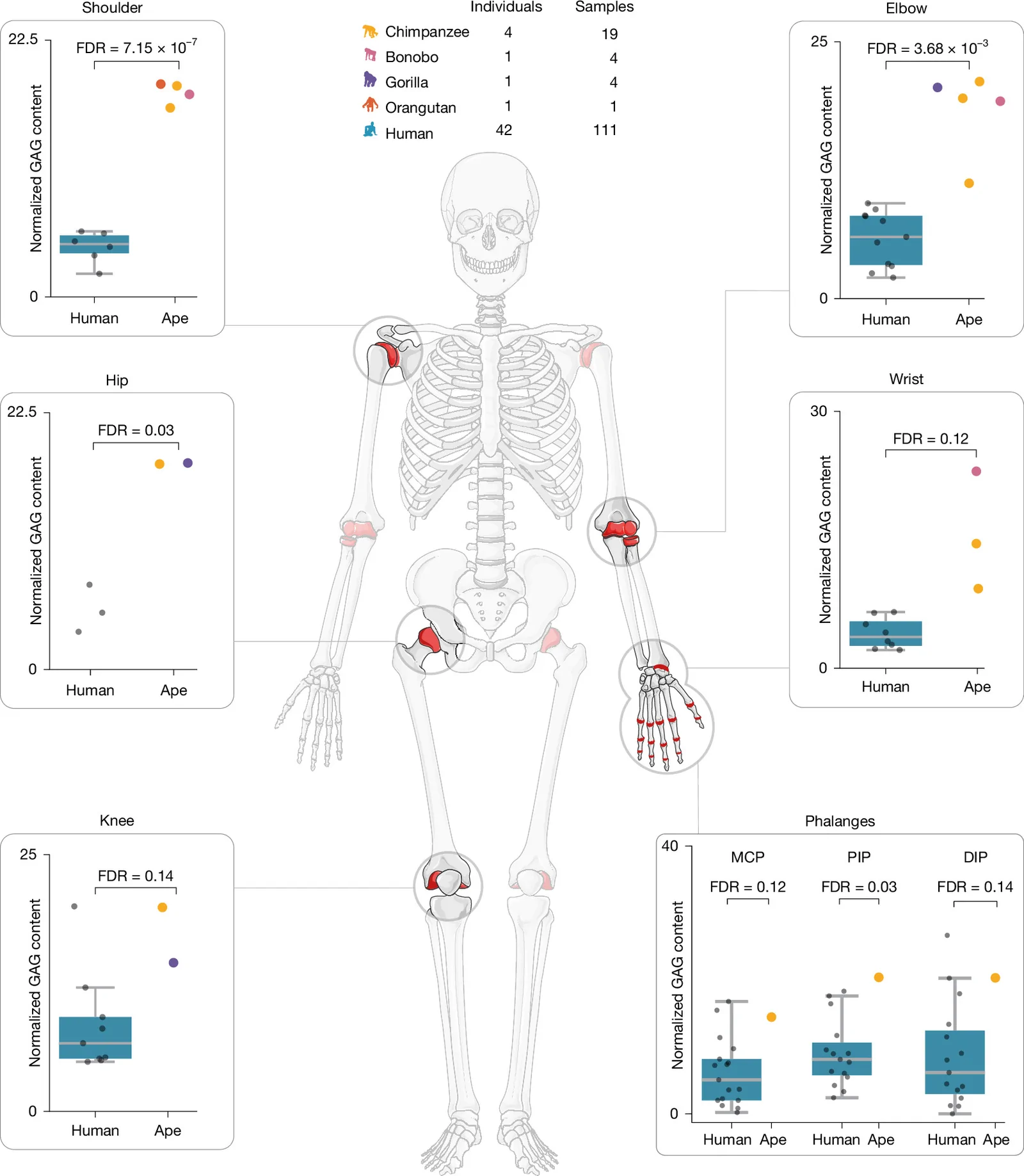
GAG content decreased in several joints during human skeletal evolution. GAG levels in humans (box plots) and great apes (coloured points) across eight joint types. Each data point shows the average across three technical replicates. sGAG content was measured using a DMMB assay and normalized to DNA content. FDR-adjusted *P* values were calculated using two-sided *t*-test for all joints, except the phalangeal joints, for which a *z*-score-based test was used because only a single ape individual was available (Methods). MCP, metacarpophalangeal joint; PIP, proximal interphalangeal joint; DIP, distal interphalangeal joint. Box plots show the median (centre), second and third quartiles (box boundaries) and the minima and maxima within 1.5× the interquartile range (whiskers). Points beyond these whiskers are plotted individually as outliers. Skeleton image adapted from Servier Medical Art (https://smart.servier.com/smart_image/skeleton-and-cartilage-face), under a Creative Commons licence CC BY 4.0.

**Extended Data Fig. 8:**
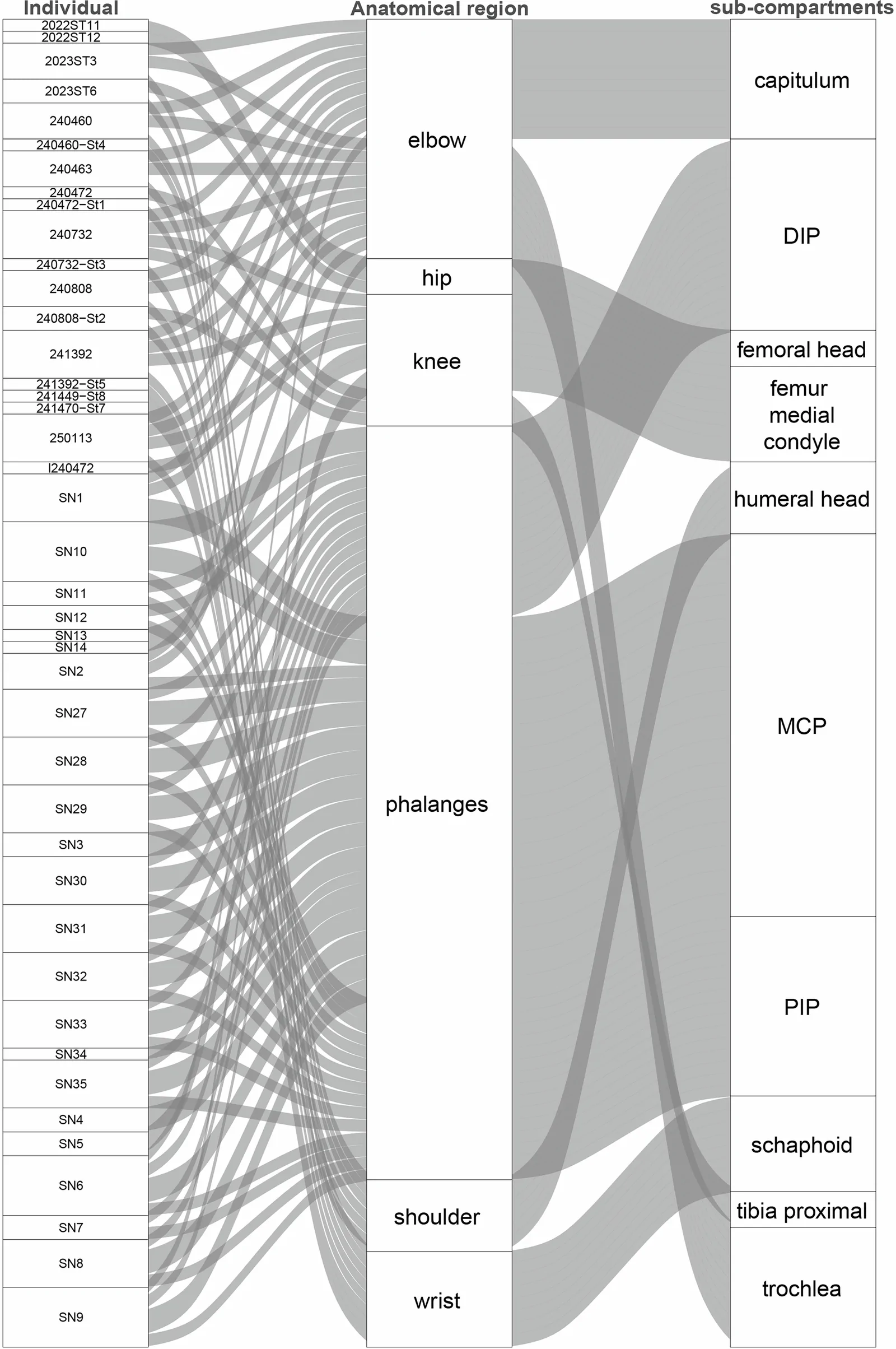
DMMB assay of human articular cartilage samples. Alluvial (Sankey) plot of the 111 human samples used in the DMMB assay, showing the individual, anatomical region and subcompartment for each sample. MCP, metacarpophalangeal; PIP, proximal interphalangeal; DIP, distal interphalangeal.

**Extended Data Fig. 9:**
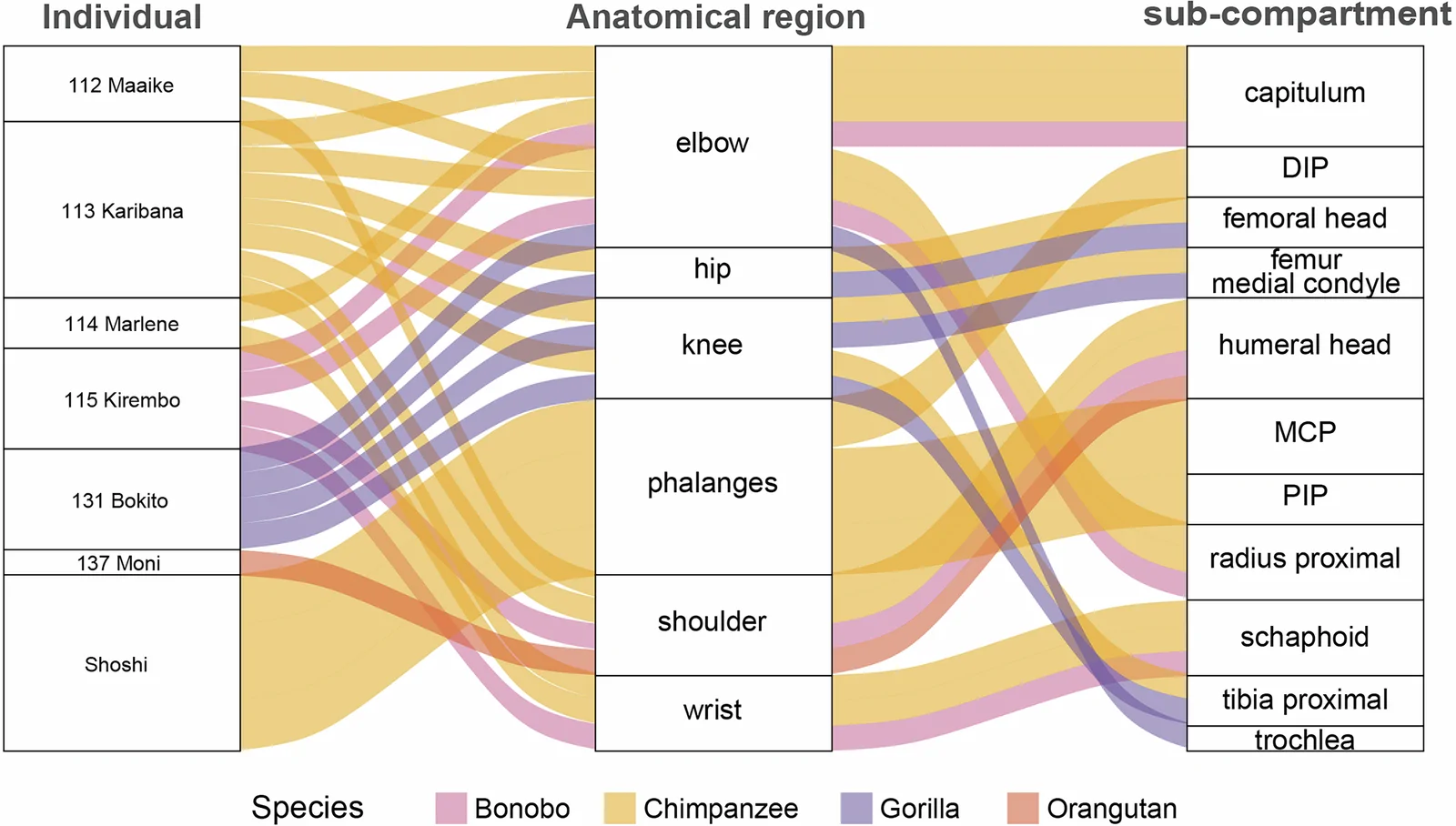
DMMB assay of ape articular cartilage samples. Alluvial (Sankey) plot of the 28 great ape samples used in the DMMB assay, showing the individual, anatomical region and subcompartment for each sample. Species is indicated by the flow colour. MCP, metacarpophalangeal; PIP, proximal interphalangeal; DIP, distal interphalangeal.

**Extended Data Fig. 10:**
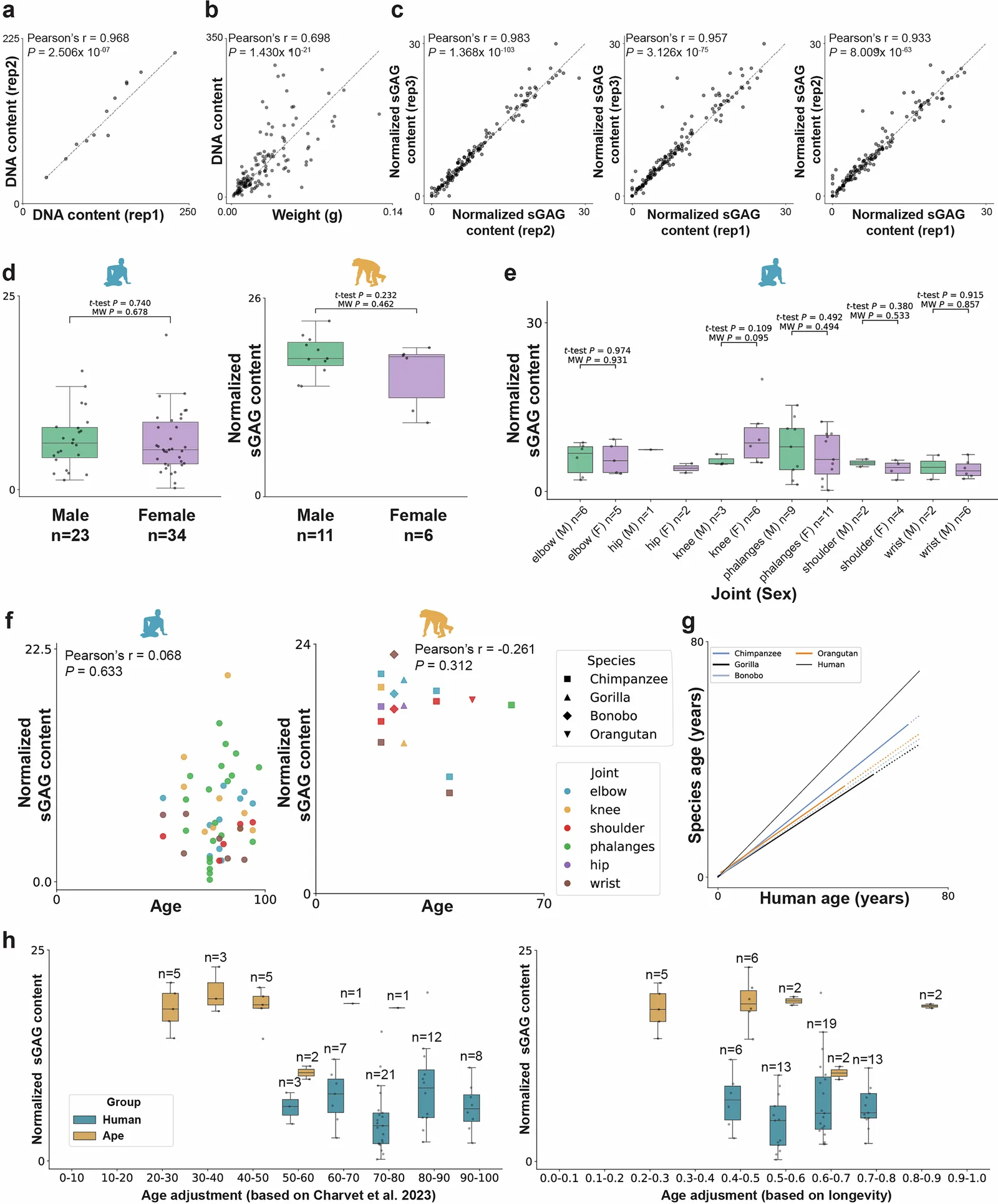
Reproducibility of DMMB assays. **a**, Correlation between DNA content across technical replicates. **b**, Correlation between DNA content and weight. **c**, Correlation across technical replicates of sGAG content normalized by DNA content. *P* values in **a**–**c** were are calculated using two-sided Pearson correlation tests. **d**, sGAG content normalized by DNA content for human (left) and great ape (right) samples, grouped by sex. P values calculated using two-sided tests. **e**, Normalized sGAG content for human samples, groups by joint and sex. *P* values calculated using two-sided tests. **f**, Correlation between age and normalized sGAG content for humans (left) and great apes (right). *P* values calculated using two-sided Pearson correlation test. **g**, Linear model used for cross-species age adjustment. Extrapolated age ranges are indicated by dashed line segments. **h**, Normalized sGAG content across age groups after age adjustment based on a previous study^145^ (left) and longevity (right). Box plots show the median (centre), second and third quartiles (box boundaries) and the minima and maxima within 1.5 times the interquartile range (whiskers). MW, Mann–Whitney *U*-test.

The opposing trends of increased GAG anchor points on the one hand and downregulation of key GAG biosynthesis genes on the other hand make it difficult to predict their net effect on GAG abundance. To determine whether the composition of human cartilage ECM differs from that of other great apes, we analysed 139 cartilage samples from 7 great ape and 42 human individuals, spanning 8 joints (Extended Data Figs. 8 and 9 and Supplementary Table 15). Sulfated GAG (sGAG) concentrations were quantified using a dimethylmethylene blue (DMMB) assay and normalized by DNA content (Methods). Whereas GAG levels are similar across non-human great apes, humans exhibit a marked threefold overall reduction (*P* = 1.5 × 10^−5^, two-sided *t*-test). This reduction is also observed at the level of individual joints, with humans showing a 2.6- to 4-fold reduction in each of the eight joints analysed (Fig. 4, Extended Data Fig. 10 and Supplementary Tables 16 and 17). These differences could not be explained by variation in age or sex (Extended Data Fig. 10 and Methods). In addition, although differences in mechanical loading can influence GAG content^48–50^, the reduction observed in humans is consistent across joints experiencing lower (elbow), comparable (thumb) or higher (hip) loading relative to great apes^51^ (Fig. 4 and Supplementary Table 17). This suggests that physiological response to mechanical loading is unlikely to be the primary driver of the reduced GAG content observed in humans. Together, these findings suggest that GAG content remained mostly stable throughout millions of years of ape evolution, but underwent a sharp and widespread reduction in the human lineage.

Reductions in GAG biosynthesis affect many morphological phenotypes, mainly skeletal ones, including short stature, facial flattening, high forehead, short fingers, thumb reorientation and more^34^. Changes in GAGs have also been implicated in shaping phenotypic divergence across species^52^. To assess the potential consequences of GAG downregulation, we applied our directional phenotyping framework^25,53–55^, and found that phenotypes driven by reduced GAG biosynthesis match particularly well with human-specific phenotypes (2.9× enrichment, *P* = 9.8 × 10^−3^, one-sided *t*-test; Extended Data Fig. 7b, Supplementary Table 18 and Supplementary Information). Of note, reduced GAG levels are also linked to several degenerative skeletal disorders, all of which are more common in humans than in non-human apes, even after controlling for age, sex and environmental factors (see ‘Discussion’ below). Together, these observations suggest that the human-specific reduction in GAG content contributed both to skeletal morphology and to the increased susceptibility of humans to degenerative skeletal diseases.

Combined, our results indicate (i) widespread *cis*-regulatory downregulation of the GAG biosynthesis pathway at both the cCRE and the gene expression level; (ii) signatures of accelerated evolution; (iii) reduced GAG content in human relative to great ape joints; and (iv) possible implications of these changes for human-specific skeletal phenotypes and diseases.

## Discussion

Identifying regulatory changes that underlie human traits is a major challenge in genetics. The human skeleton represents a system that underwent extensive phenotypic alterations in human evolution^1^, but whose underlying genetics remains poorly understood. Here, we have presented a framework for dissecting how *cis*-regulatory evolution shaped the human skeleton. Using MPRA, we quantified the regulatory activity of the 561,410 single-nucleotide substitutions that arose and reached fixation in human promoters and enhancers, and integrated these measurements with gene expression differences in human–ape hybrid cells. This approach yielded atlases of the *cis*-regulatory changes in human skeletal evolution (Supplementary Tables 4 and 8). Analysis of these atlases revealed many signatures of ECM divergence, including widespread suppression of GAG biosynthesis, signatures of lineage-specific selection and two human-specific expansions of GAG anchor repeats in the core ECM protein aggrecan.

At the phenotypic level, we found pronounced divergence in skeletal tissue composition (Fig. 4), with human tissues containing threefold less GAG than those of non-human apes. Alterations in GAG biosynthesis affect susceptibility to several skeletal disorders, particularly intervertebral disc degeneration, disc herniation and degenerative joint disease (osteoarthritis)^36,56–59^. Previous studies have reported a 25–38% reduction in GAG content in patients with these conditions, relative to healthy control individuals^58,59^. The evolutionary change we detected in humans compared with great apes is twice as large (−62%), suggesting that it might have pushed human cartilage towards a threshold of vulnerability—such that all humans carry a baseline GAG deficit that primes the joint for degeneration. Indeed, humans have a substantially higher prevalence of degenerative skeletal diseases than do non-human primates^3,4^. The most notable difference is observed in osteoarthritis: this highly heritable disease is the most common skeletal disorder in humans, affecting hundreds of millions of people worldwide and constituting a major cause of disability and reduced quality of life. However, in great apes, it is much rarer, even after accounting for age, sex and environment^3–5^. Knee-specific regulatory divergence might have further increased the prevalence of knee osteoarthritis in humans^60^. Consistent with the role of decreased GAG in osteoarthritis, direct intra-articular administration of GAGs is used therapeutically to manage osteoarthritis^61^, indicating that restoring GAG levels towards the ancestral state helps to alleviate the symptoms of the disease. Together, these observations suggest that the human-specific reduction in GAG content is likely to have not only shaped human skeletal morphology, but also increased our vulnerability to degenerative skeletal diseases.

Whereas great apes show conservation in the GAG pathway at both the genetic and the phenotypic level (Figs. 3 and 4), the shift in human GAG biosynthesis shows mixed signatures of selection. On the one hand, some observations are compatible with a relaxation of negative selection, because in the absence of selection, gene expression levels are more likely to decrease than they are to increase^62,63^, and within-species phenotypic variation tends to increase^64^. On the other hand, several other observations are more difficult to reconcile with purely relaxed constraint, and instead point to non-neutral, potentially adaptive changes: (i) the extent of downregulation exceeds neutral expectations derived from saturation mutagenesis experiments^62^; (ii) at the protein level, GAG genes show conservation levels comparable with those of other genes (*P* = 0.635, two-sided *t*-test on non-synonymous versus synonymous changes (dN/dS; ref. ^65^); and (iii) the differences in GAG content are large and tightly associated with substantial effects on multiple phenotypes and diseases^57^, making neutrality less likely. Furthermore, the increase in GAG anchor points in aggrecan could itself reflect either relaxed negative selection or a compensatory adaptive response to reduced GAG levels. Regardless of whether these changes reflect adaptation or relaxation of negative selection, they point to a shift in the selective pressures shaping human skeletal biology, with implications for both skeletal morphology and health.

Given the strong correlation in MPRA differential activity across cell types^12^, many of the differentially active cCREs identified here are likely to exhibit differential activity in non-skeletal tissues as well. GAGs also have essential roles in non-skeletal tissues; for example, serving as viral co-receptors and moderators of brain development and function^66^. One of the most notable GAG-related changes in human evolution is the complete loss of the ability to produce *N*-glycolylneuraminic acid—the predominant sialic acid in most organs of non-human great apes^67^.

Although our study focused on evolution, its scope also enables applications beyond evolutionary analyses. In particular, our MPRA library covers approximately 40% of all putative enhancers in the human genome^21^, and thus, the activity measurements we report could help researchers to distinguish which of these candidates might function as true enhancers in chondrocytes.

## Methods

### MPRA design

#### Library design

To generate the catalogue of fixed human-derived substitutions we used genotyping data from 139 non-human great apes^15,68–71^ (10 bonobos, 59 chimpanzees, 43 gorillas and 27 orangutans), all mapped to the human reference genome GRCh37. To restrict the analysis to high-confidence genotypes, we excluded variants that did not meet any of the following criteria: DP ≥ 5 or DP ≥ sample 2.5% percentile, DP ≤ 100 or DP ≤ sample 97.5% percentile, GQ ≥ 20, allelic balance ≥ 0.75 for homozygous calls or between 0.25 and 0.75 for heterozygous calls. In addition, to reduce false positives arising near indel calls, we excluded variants located within ±10 bp of an indel.

To generate a catalogue of substitutions that are likely to be derived and fixed in humans, and fixed for the ancestral allele in all non-human great apes, we performed the following filtering steps. First, to include only positions that are fixed in the human population, we used substitutions that are completely (100%) fixed for the human reference allele in 19 human individuals from the great ape catalogue^15,68–71^ and do not have an alternative allele in any of the 15,708 human genomes in gnomAD v.2.1.1 (refs. ^16,72^; we then validated the variant frequencies in additional datasets, see below). Overall, 99.9963% of positions had at least 1,000 genotyped individuals (read coverage > 5). Second, we restricted the analysis to sites fixed (100%) for the non-human allele across all non-human great ape samples with genotypes passing the above filters. Only positions with valid genotypes in at least 80% of individuals of each group (*Pan*, *Gorilla* and *Pongo*) were kept. Multi-allelic alternative variants were excluded as well. Third, to retain variants shared across all human groups, including Neanderthals and Denisovans, we further filtered the dataset by using the variant call files generated using the four high-coverage archaic genomes and excluding sites at which any archaic humans carried an alternative allele^17–20^ using standard parameters (removing indels; keeping FILTER = ‘PASS’ or ‘.’; DP ≥ 10; QUAL ≥ 20; GQ ≥ 20). Fourth, for technical limitations in downstream synthesis and cloning, we excluded variants overlapping RepeatMasker repetitive regions (‘low complexity’, ‘simple’ and ‘satellite’ repeat types)^73^, and simple tandem repeats from Tandem Repeats Finder^74^, both downloaded from the UCSC table browser^75^. Finally, we excluded variants overlapping the ENCODE set of problematic genomic regions^76^. This resulted in a catalogue of 5,731,772 single-nucleotide variants.

We validated the frequencies of all variants using two datasets. First, in gnomAD v.4.0 (ref. ^16^), we found that 80.5% of the variants remained completely fixed in humans, 99.96% had a frequency higher than 0.999 and 99.99% had a frequency higher than 0.99. Second, we used a combined dataset of the 1000 Genomes Project (1KG) and the Human Genome Diversity Project (HGDP), downloaded from gnomAD v.3.1 (ref. ^16^). This dataset enables a more accurate estimation of global allele frequency. Here, 96.8% of the variants were completely fixed, 99.91% had a frequency higher than 0.999 and 99.99% had a frequency higher than 0.99.

Libraries of this size are beyond the current capacity of lentiMPRA. Thus, we further filtered the catalogue for substitutions within candidate cCREs. To this end, we used SCREEN (v.2), the ENCODE project’s database of cCREs defined by chromatin marks^21^. SCREEN contains 926,535 cell-type-agnostic cCREs, calculated on the basis of 839 human tissues and cells. Elements with the following classifications were included: (1) enhancer-like cCREs; (2) promoter-like cCREs; and (3) DNase-H3K4me3 cCREs, which together cover 7.62% of the genome. This filtering increases the likelihood that a sequence is active by threefold^12^. This resulted in an initial within-cCRE catalogue of 472,528 substitutions.

On the basis of this variant list, we designed a library of 270-bp DNA sequences centred around substitutions in the catalogue. To reduce pool size, adjacent substitutions (≤170 bp apart) were synthesized in the same sequence, centred midway between the outermost substitutions. If additional substitutions fell within the first or last 50 bp of a sequence, they were also included. However, these substitutions (i) did not affect the centring, and (ii) to control for potential edge effects associated with substitutions located near the boundaries of the synthesized sequence, an additional construct centred on each such flanking substitution was designed. Regardless of the number of substitutions in a sequence, each sequence was synthesized twice: once with the great ape ancestral sequence and once with the full set of human-derived substitutions (including substitutions outside SCREEN elements). Sequences overlapping repetitive or problematic regions by more than 25% were excluded. This resulted in a library of 343,503 sequence pairs covering 524,751 substitutions, each represented by its ancestral and derived versions. Because this number exceeds the capacity of a single lentiMPRA, we randomly divided the library into nine sublibraries of around 80,000 sequences. To minimize potential batch effects between sequence pairs, we included both alleles of each sequence within the same sublibrary.

A tenth sublibrary included two groups of sequences. First, to take into account promoter strand-specific regulation^77^, the 5,942 sequences (2,971 sequence pairs) that overlapped with promoters of genes on the minus strand were resynthesized in their native reverse orientation. Promoters were defined as regions ± 500 bp away from a transcription start site (TSS)^78^. Sequences overlapping promoters of both plus-strand and minus-strand genes were treated as plus-strand sequences.

Second, substitutions in CpG islands^79^ were underrepresented in the original library owing to lower sequencing coverage in the ape genomes. To rescue them, we relaxed the minimum percentage of individuals with a valid genotype required per species group from 80% of all individuals to 30% of individuals with high CpG island coverage in each species group. This translated to 22% in *Pan*, 20% in *Gorilla* and 12% in *Pongo* and added 45,798 sequences (22,899 sequence pairs). Owing to PCR limitations observed in the other nine libraries, sequences with a GC content lower than 43% were excluded. In total, among genomic positions annotated as cCREs by SCREEN v.2 (ref. ^21^) (7.62% of the genome), 13.98% did not pass genotype quality control, 1.90% overlapped repetitive or problematic regions and 2.54% were located near indel calls. After accounting for overlaps between these regions, 15.59% of the cCRE genomic territory was excluded. The remaining 84.41% was retained for identification of human-derived substitutions. Overall we synthesized 366,402 sequence pairs containing 561,410 single-nucleotide substitutions (Supplementary Table 1).

Analyses were performed using BEDTools^80^, VCFtools^81^ and BCFtools^82^. Unless otherwise mentioned, we used GRCh37 as the reference genome in all analyses. The UCSC liftOver tool was used to lift over coordinates between genome assemblies^83^.

#### MPRA controls

To estimate the quality of the experiment, and to compare performance across sublibraries, each sublibrary included the following set of controls (Supplementary Table 1). (1) Positive controls for regulatory activity included: (i) 138 sequences previously shown to be active in a published lentiMPRA performed in osteoblasts, the cell type most closely related to chondrocytes (100 of those were also found to be active in embryonic stem cells and/or neural progenitor cells)^12^; and (ii) 12 previously validated CREs active in chondrocytes^84–89^. In total, there are 150 positive controls for activity in each sublibrary. (2) Negative controls for activity: (i) 100 sequences showing no activity in any of the three tested cell types (including osteoblasts) in a previous lentiMPRA^12^; (ii) 100 scrambled sequences, generated by randomly shuffling 100 sequences from each sublibrary; and (iii) non-SCREEN controls; that is, 100 sequences centred around human-derived fixed variants that do not fall within SCREEN-defined putative regulatory regions, but do fulfil all other criteria of our library design. In total, we included 300 negative controls for activity in each sublibrary. (3) Positive controls for differential activity: 100 sequence pairs that showed differential activity in a previous lentiMPRA^12^. In addition, to gain insight into their activity in chondrocytes, sublibrary L3a3 also included the other 307 pairs of differentially active modern-human-derived sequences, allowing all 407 of the reported differentially active sequences to be tested in chondrocytes.

### MPRA experiment

#### Library cloning

Fifteen-base-pair primer-binding sequences were added to both sides of each 270-bp sequence (Supplementary Table 1), and synthesized in three batches of 240,000 sequences by Twist Bioscience. The tenth sublibrary was synthesized in an additional batch. Each sublibrary was amplified separately by an eight-cycle PCR using sublibrary-specific forward primers (5BC-AG01-f01, 5BC-AG02-f01, 5BC-AG03-f01; Supplementary Table 19) that contain a vector overhang sequence and reverse primers (5BC-AG01-r01, 5BC-AG02-r01 and 5BC-AG03-r01; Supplementary Table 19) that add a minimal promoter (mP) downstream of the test sequence. A second round of nine-cycle PCR was performed with a forward primer (5BC-AG-f02; Supplementary Table 19) and a reverse primer (5BC-AG-r02; Supplementary Table 19) that adds a 15-bp random barcode downstream of the mP. The amplified fragments were then inserted into the AgeI and SbfI sites of the pLS-SceI vector (Addgene, 137725) using the NEBuilder HiFi Master Mix (NEB). The recombination product was electroporated into 10-beta competent cells (NEB) using a Gemini X2 electroporation system (BTX). The transformed cells were cultured overnight on 15-cm 100 mg ml^−1^ carbenicillin LB agar plates, and the resulting plasmid library was extracted using the QIAGEN Plasmid Plus Midi Kit (QIAGEN). We collected approximately 16 million colonies per sublibrary, yielding an average of 200 barcodes associated with each test sequence.

#### cCRE–barcode association

To determine the association between barcodes and cCREs, we first amplified a fragment containing the test cCRE, mP and barcode from each sublibrary using primers that contain Illumina flow cell adapters (P5-pLSmP-ass-i# and P7-pLSmp-ass-gfp; Supplementary Table 19). The amplified fragments were sequenced with a NextSeq 550 using custom primers for each sublibrary (R1, pLSmP-ass-seq01-R1, pLSmP-ass-seq02-R1, pLSmP-ass-seq03-R1; R2, pLSmP-ass-seq-ind1; R3, pLSmP-ass-seq01-R2, pLSmP-ass-seq02-R2, pLSmP-ass-seq03-R2; R4, pLSmP-rand-ind2; Supplementary Table 19).

The R1 and R3 read pair covered the test sequence and the R2 index read covered the barcode. We obtained at least 60 million reads for each sublibrary. We then used Bowtie2 (ref. ^90^) to map the read pair covering the test sequence to the original list of 732,804 test sequences, using the preset parameters ‘—very-sensitive’. Next, we kept only read pairs that (i) had the ‘proper pair’ SAM designation; (ii) mapped to the same sequence; and (iii) had at least one read with a mapping quality ≥ 6. After linking each read pair covering the test sequence to the read covering the barcode, we removed barcodes that (i) had a sequencing quality score ≤ 30 for any of the 15 bases of the R2 read; (ii) were associated with multiple sequences; or (iii) had fewer than two independent associations linking the barcode to the sequences. This resulted in a final list of barcode–sequence associations containing 112,470,912 unique barcodes and 684,577 sequences. Data were deposited in the NCBI Gene Expression Omnibus (GEO) under accession number GSE316891.

#### LentiMPRA experiment

Human primary chondrocyte culture was performed according to the manufacturer’s instructions (Cell Applications, 402K-05f). In brief, the cells were maintained in HC basal medium with HC growth supplement. For passaging, cells were dissociated using trypsin and EDTA and plated at approximately 30,000 cells per cm^2^.

Lentivirus packaging was performed as previously described^91^. In brief, 50,000 cells per cm^2^ HEK293T cells were seeded in T175 flasks and cultured for 48 h. The cells were co-transfected with (per flask) 7.5 μg of plasmid libraries, 2.5 μg of pMD2.G (Addgene, 12259) and 5 μg of psPAX2 (Addgene, 12260) using EndoFectin Lenti transfection reagent (GeneCopoeia) according to the manufacturer’s instructions. After 8 h, the cell culture medium was refreshed and ViralBoost reagent (ALSTEM) was added. The transfected cells were cultured for 2 days to complete lentivirus packaging. The lentiviruses in the culture medium were concentrated using the Lenti-X concentrator (Takara) according to the manufacturer’s protocol, and resuspended in 1,500 μl phosphate-buffered saline (PBS) per T175 flask. Lentiviral infection, DNA and RNA extraction and barcode sequencing were performed as described previously^91^. In brief, for each sublibrary, three biological replicates were performed. Eight million chondrocytes were seeded into three 10-cm dishes (2.7 million cells per dish), and cultured for 24 h. Chondrocytes were infected with the lentivirus libraries at a multiplicity of infection (MOI) of 50–100 using ViroMag according to the manufacturer’s protocol. We used 150 μl lentivirus and 220 μl ViroMag per 10-cm dish. Infected cells were grown for 3 days, and total RNA and genomic DNA were extracted using the QIAGEN AllPrep mini kit (QIAGEN). The extracted RNA was purified using the TURBO DNA-free Kit (Thermo Fisher Scientific) and reverse-transcribed into cDNA with SuperScript IV (Thermo Fisher Scientific), using a barcode-specific primer containing a unique molecular identifier (UMI) (P7-pLSmp-ass16UMI-gfp; Supplementary Table 19). Barcode fragments were amplified from both genomic DNA and cDNA, with three-cycle PCR using the UMI primer (P7-pLSmp-ass16UMI-gfp; Supplementary Table 19) and a primer that contained a sample index sequence (P5-pLSmP-5bc-i#; Supplementary Table 19). A second round of PCR was performed to amplify the library using primers containing flowcell adapters (P5 and P7; Supplementary Table 19). The barcode fragments were purified using Ampure XP (Beckman Coulter), pooled and sequenced with NextSeq 15PE using custom primers (R1, pLSmP-ass-seq-ind1; R2 (read for UMI), pLSmP-UMI-seq; R3, pLSmP-bc-seq; R4 (read for sample index), pLSmP-5bc-seq-R2; Supplementary Table 19).

### MPRA processing

#### Barcode RNA and DNA sequencing and processing

Per sublibrary, 50–110 million reads were sequenced for DNA and 120–320 million reads for RNA (data deposited in the NCBI GEO under accession number GSE316891). We aligned the R1 and R3 reads to the barcode–sequence association list using Bowtie2 (ref. ^90^) with the preset parameter option ‘—very-sensitive’. Next, we applied quality filters to the alignment. We kept only read pairs (i) that had ‘proper pair’ SAM designation; (ii) in which both reads had a mapping quality ≥ 20; and (iii) in which CIGAR string and MD Flag showed a perfect match (15M and 15, respectively). Finally, we removed PCR duplicates by collapsing reads on the basis of UMIs.

Some cCREs with multiple barcodes had one or more barcodes with extreme RNA read counts compared with the other barcodes of the same cCRE allele. Such outliers are likely to be due to hyperactive integration loci, and do not represent the true endogenous activity of the cCRE. To minimize such cases, we removed barcodes that had an RNA count higher than or lower than two standard deviations from the mean RNA barcode counts per cCRE allele, as described in our MPRA quality-control pipeline^23^. On average, 3.58% of barcodes were removed per replicate (324,822 in total; 4.39 per cCRE).

#### Quantifying activity

We used the R package MPRAnalyze^22^ (v.1.9.1) to analyse the MPRA data. We provided UMI-collapsed read abundances for each barcode as input. To determine which cCREs were capable of promoting expression, we used the RNA and DNA models of the quantification framework of MPRAnalyze (rnaDesign = ~1 and dnaDesign = ~replicate) and extracted alpha, the transcription rate, for each cCRE. MPRAnalyze uses the activity of the scrambled sequences as a baseline against which to estimate the expression level of each tested cCRE. We corrected the mean absolute deviation (MAD) score-based *P* values from MPRAnalyze for multiple testing across tested cCREs in all sublibraries, excluding any controls and cCREs with fewer than five DNA counts, using the Benjamini–Hochberg method^92^, thus generating a MAD-score-based activity FDR for each cCRE. We defined cCREs as capable of driving expression (active) if they had an FDR ≤ 0.05. As an additional measure of activity, we calculated a simple ratio of expression as RNA abundance normalized to DNA abundance (RNA/DNA ratio). To do so, we aggregated UMI-collapsed read abundances across all barcodes of each cCRE, separately for DNA and RNA. We then added pseudocounts (+1) to the resulting RNA and DNA counts. Next, we used counts per million (CPM) normalization to correct these counts according to library size. Finally, we divided the normalized RNA counts by the normalized DNA counts. We repeated this calculation for each replicate separately and for the combined outputs of all replicates. Overall, we identified 65,576 active cCREs (Supplementary Table 2).

#### Quantifying differential activity

We used the comparative framework of MPRAnalyze^22^ to measure differential activity between the human and great ape alleles. MPRAnalyze uses information across all the barcodes for both alleles of a given cCRE, as well as information across all replicates (note that because of run-time issues for the sequence pair seq325030, which had the highest number of barcodes in its sublibrary (10,933), we randomly subsampled approximately half (5,486) of the barcodes for this specific pair. For all other sequence pairs, we used information from all barcodes). To reflect the design of our experiment, we included barcode, allele and replicate information in the DNA model and allele information in the RNA model (rnaDesign = allele, dnaDesign = replicate + barcode_allele, reducedDesign = 1). The differential activity analysis included all cCREs in which at least one of the alleles was defined as active (see above), as well as the active positive and negative controls for differential activity. Then, we extracted the MPRAnalyze *P* values and differential activity estimate (fold change) of the human relative to the great ape allele. Using the Benjamini–Hochberg method^92^, we corrected the *P* values of all sublibraries for multiple testing (excluding controls). We defined all cCREs with FDR ≤ 0.05 as differentially active. Overall, we found 15,077 differentially active cCREs (Supplementary Table 4).

### Chondrocyte ATAC-seq and CUT&Tag

ATAC-seq was performed in two biological replicates following the manufacturer’s protocol (Tagment DNA Enzyme and Buffer Small Kit; Illumina) with modifications. In brief, the 500,000-chondrocyte cell pellet was washed with PBS and resuspended in 50 μl lysis buffer to isolate nuclei. The nuclei were pelleted and resuspended in 50 μl of a transposition reaction mixture containing 25 μl Tagment DNA buffer and 2.5 μl Tagment DNA enzyme, then incubated at 37 °C for 30 min. Tagmented DNA was purified using the MinElute reaction cleanup kit (QIAGEN).

CUT&Tag was performed in two biological replicates using the Hyperactive In-Situ ChIP Library Prep Kit (Vazyme) according to the manufacturer’s protocol, with minor modifications. In brief, 500,000 cells were washed with 250 μl wash buffer and resuspended in 50 μl wash buffer. Cells were incubated with 5 μl of ConA beads for 5 min and collected using a magnet stand. The ConA-bound cells were resuspended in 50 μl antibody buffer and incubated with an anti-H3K27ac antibody (ab4729, Abcam) at 4 °C overnight. Cells were then collected using a magnet stand and further incubated with goat anti-rabbit IgG (ab6702, Abcam) at room temperature for 2 h. Cells were then washed twice with wash buffer and incubated with pA-Tn5 at room temperature for 1 h. After two additional washes, cells were incubated in tagmentation buffer at 37 °C for 1 h to activate tagmentation. The tagmented DNA was treated with 5 μl of 0.5 M EDTA, 1.5 μl of 10% SDS and 1.25 μl Proteinase K, followed by phenol–chloroform extraction and ethanol precipitation.

The tagmented DNA extracted from ATAC and CUT&Tag was size-selected twice using 0.65×/1.8× SPRIselect (Beckman Coulter) according to the manufacturer’s protocol. Library amplification was performed as previously described^93^. The amplified libraries were further purified twice with SPRIselect and quantified on a TapeStation using the High Sensitivity D1000 kit (Agilent).

The ATAC-seq and CUT&Tag libraries were pooled and sequenced on an Illumina NextSeq platform to generate 80-bp paired-end reads using a Mid Output 150-cycle kit. Sequencing quality was assessed with FastQC v.0.12.1 (http://www.bioinformatics.babraham.ac.uk/projects/fastqc/). Adapter sequences were removed and reads were quality-trimmed using fastp v1.0.1 (ref. ^94^) with default parameters. Trimmed reads were aligned to the GRCh38 reference genome using bowtie2 v.2.5.1 (ref. ^90^) with the following parameters: –very-sensitive –dovetail -I 0 -X 700. PCR duplicates were removed using Picard MarkDuplicates v.3.0.0 (http://broadinstitute.github.io/picard). Peaks were called using MACS3 v3.0.1 (ref. ^95^) with the following parameters: -f BAMPE -g hs –nomodel –qvalue 0.05.

Data have been deposited in the DDBJ database under accession number PRJDB40123.

### TF-binding sites

We compared two versions of each variant in the library: one containing the reference human allele and the other containing the alternative (great ape) allele. Each cCRE was associated with potential binding TFs using two independent approaches: find individual motif occurrences (FIMO) and protein binding microarray (PBM). For this analysis, we used only TFs that have been shown to be expressed in chondrocytes (that is, with gene transcripts per million (TPM) > 1)^96^. The code used for this analysis has been deposited at https://github.com/GokhmanLabOrganization/differential-TF-binding.git.

#### Predicted-motif-based approach (FIMO)

Position frequency matrices were downloaded from JASPAR^97^ as MEME files (JASPAR2024_CORE_vertebrates_nonredundant_pfms_meme). For TFs with multiple reported motifs, we selected a single representative motif using the following prioritization scheme. (i) Motifs derived from in vitro assays (SELEX, HT-SELEX, CAP-SELEX, SMiLE-seq or PBM) were prioritized over in vivo chromatin immunoprecipitation followed by sequencing (ChIP–seq)-derived motifs, ensuring that comparisons were either between in vitro and ChIP–seq or within the same platform. (ii) When multiple motifs originated from the same assay type, we selected the one supported by the highest number of input sites used to determine the motif in the original study (‘nsites’). (iii) If nsites were identical (for example, closely related alternatives such as C/G versus CG), we selected the longer motif.

For each variant, we extracted the surrounding sequence and generated reference and alternative allele versions. Sequence length was set by the longest motif in the .meme file, ensuring that the variant is centred and all motif positions are covered. The sequences were written to a FASTA file and analysed with FIMO^40^ using the following command: fimo --text --thresh 1e-4.

FIMO reports scores only for statistically significant matches, but allele comparisons require scores for both sequence versions. Therefore, when a significant match was detected for one allele and the other was missing, we added the corresponding sequence and recalculated binding scores for all sequences to ensure consistency. Scores were computed using the same PWMs as in the .meme file, summing position-wise contributions across the sequence following FIMO’s logic. Significance was assessed by comparing each score with background sequences, with *P* values defined as the fraction of background scores that were greater than equal to the observed score. We confirmed that this approach reproduces FIMO’s scoring with high correlation. Finally, for each variant, we computed the differential binding score as the human allele version motif score minus the ancestral motif score (because FIMO output scores are expressed in a logarithmic scale).

#### Experimental binding data (PBM)

We further used PBM data to quantify TF–DNA interactions. For each variant, we generated all possible 8-mers spanning the variant in every position (eight sequences in total), extracted their binding intensities from PBM data (PBM median fluorescence values) and calculated the differential binding in the same way as for FIMO.

The PBM 8-mer data were taken from UniPROBE^98^ and CIS-BP^99^, downloaded from the NCBI GEO under accession number GSE53348. We retained pairs in which at least one 8-mer had a median intensity > 0.35. *P* values were derived from corresponding *z*-scores and then corrected for multiple comparisons (*q*-values). Scores with *q* > 0.05 were discarded, and only allelic pairs passing this filter for both alleles were used for differential binding analysis.

#### TF statistical analysis

Data were filtered to include only cCREs with a single variant to avoid confounding effects from multiple variants. Fisher’s exact test was used to test for the enrichment of TF-binding sites in differentially active cCREs versus all active cCREs. For each TF, a contingency table was constructed with the categories: TF binding/differential activity; TF binding/no differential activity; no TF binding/differential activity; and no TF binding/no differential activity. Odds ratios and *P* values were calculated for each TF, and *P* values were adjusted for multiple testing using the Benjamini–Hochberg FDR correction.

Pearson’s correlation coefficients were calculated between differential TF binding *z*-scores and cCRE fold change (ln), for cCREs with significant differential activity. *P* values were adjusted for multiple testing using FDR correction (Benjamini–Hochberg method).

### Human accelerated regions and quickly evolved regions

Some sequences have been shown to evolve rapidly along the human lineage. These include human ancestor quickly evolved regions (HAQERs)^100^ and human accelerated regions (HARs)^101,102^. So far, the majority of accelerated regions with known functional effects have been associated with neural processes^103,104^, but a few have been implicated in skeletal phenotypes^13,105^. We found that only six HARs contain fixed human-derived substitutions and intersect with active chromatin marks, and none show differential activity. HAQERs, however, show an enrichment in differentially active sequences (2.84×, *P* = 0.0195, two-sided Fisher’s exact test), suggesting that the emergence of HAQERs might have been associated with shifts in gene regulation, in line with results in neural^100^ and skeletal^13^ tissues.

### Association of MPRA cCREs with target genes

To predict the genes linked to each cCRE, we used five approaches (Supplementary Table 9). (i) Overlap with promoters: we defined promoters as the region 5 kb upstream to 1 kb downstream of NCBI GRCh37 transcripts^106^, and assigned each cCRE to all the promoters in which it fell. (ii) Proximity to a TSS: because many CREs preferentially affect their nearest gene^107–109^, we linked each cCRE to its closest TSS. (iii) Proximity to known eQTLs: we used GTEx eQTLs and their associated genes from all tissue types^110^, and intersected eQTLs with our set of cCREs. We linked each cCRE to the target gene(s) of any eQTL within ±1 kb. (iv) Spatial interaction with a promoter: we used high-throughput chromosome conformation capture (Hi-C) to map spatial interactions between the cCREs and their target genes. To this end, we used Hi-C data^111–113^ from chondrocytes and mesenchymal stem cells, as a close proxy for chondrocytes. We intersected each cCRE with the promoters it interacted with, up to ±1 kb away. (v) Association with eRNA: eRNAs have been shown to be significantly co-expressed with the promoters they regulate^114^. Thus, if a cCRE overlapped an eRNA-expressing locus, we associated it with the genes co-expressed with that eRNA. We downloaded FANTOM5 eRNA data^115^ from GeneHancer^31^, which contains a list of eRNA coordinates and their associated genes.

To enrich for cCRE–gene links that are relevant to chondrocytes, we retained only links to genes expressed in chondrocytes by using two datasets from the ENCODE portal^116^ (https://www.encodeproject.org/) with the following identifiers: ENCSR000CUE and ENCSR774MGO. We defined genes as expressed if they had an expression level higher than 1 TPM). After combining both datasets and their replicates, we defined 14,206 genes as expressed in chondrocytes.

For analyses in which the cCRE–gene associations should be particularly reliable (enrichment analyses, and selection of top candidates; see below), we used a stricter subset of cCRE–gene associations that we termed ‘elite associations’. Elite associations were defined as cCREs either that are located within the promoter of a gene, or where at least two independent methods agreed on the same target gene.

### Functional enrichment analyses

Genes associated with differentially active cCREs using elite associations (*n* = 1,280; Supplementary Table 10) were tested for functional enrichment using GO^33^, the HPO database^34^ (release 2024-04-26) and the GAD^35^. To control for potential biases arising from factors such as variant choice, MPRA design or the genomic distribution of SCREEN elements, we used as background all genes linked to active cCREs (*n* = 9,975; Supplementary Table 10). Thus, the test and background gene sets differed only by whether their variants alter gene activity. Differential activity was defined using a minimum threshold of |FC| > 1.333; cCREs below this threshold were treated as active but non-differentially active and included in the background set. Hypergeometric test *P* values were calculated for each term. To reduce multiple-testing burden, terms associated with fewer than 20 differentially active genes were excluded. For HPO enrichment, we used only phenotypes related to the skeletal system, as defined by Gene ORGANizer^117^.

Similarly, we ran an enrichment analysis on the set of 4,463 human-derived differentially expressed genes, comparing them to the set of expressed, but non-differentially expressed, genes. The IPA enrichment analysis was run on version 159584291, 6 May 2026. Input included the set of 4,463 human-derived differentially expressed genes along with their fold change and FDR-adjusted *P* values for differential expression.

### GAG biosynthesis selection analysis

We defined GAG-related genes as those associated with any of the four GAG metabolic pathways in KEGG^38^. This included 73 genes related to GAG synthesis (hsa00532, hsa00534 and hsa00533) or degradation (hsa00531). For simplicity, in Fig. 3d, we show only genes related to the synthesis of the linker or chain region. Genes related to sulfation patterns were not included. Dermatan sulfate was not included, because it does not bind to aggrecan^37^. For keratan sulfate, only KSII (*O*-linked) biosynthesis was included, because it is the mode of glycosylation in aggrecan^118^.

The lineage-specific selection test was performed by comparing the cCREs associated with the 73 GAG genes described above with other cCREs. We first defined a set of cCREs that met three criteria: (i) were significantly active; (ii) had a minimum of 50 barcodes for both the ancestral and human-derived alleles; and (iii) were linked through elite associations to genes expressed in chondrocytes. This yielded a total of 40,941 cCREs. Of these, 11,700 active and 2,768 differentially active cCREs were linked to KEGG pathway annotations^38^. For cCREs that were active but not differentially active, the ln(fold change) was set to zero. To generate a neutral expectation, we randomized fold-change values across cCREs, while maintaining their gene associations. This procedure was repeated *n* = 500,000 times. We then compared the observed sum of ln(fold change) associated with each of the four GAG pathways to *n* random samplings (*S*^rand^) of the same number of cCREs affecting the same number of genes. For stringency, we required that each randomized sample include at least one differentially active cCRE. Statistical significance was assessed by computing a *P* value as the fraction of random iterations in which the sum of ln(fold change) was at least as extreme as the one observed (*S*_obs_) for each pathway, as defined below:

$$P=\frac{{\sum }_{i=1}^{n}({S}_{i}^{\mathrm{rand}}\ge {S}_{\mathrm{obs}})+1}{n+1}.$$

### Cell-type specificity analysis

cCREs for 13 cell types were downloaded from SCREEN v.4 on 24 April 2026. We filtered the available SCREEN datasets to the ‘core’ collection. Because the available chondrocyte cell type in SCREEN is in vitro differentiated, we compared it with other in-vitro-differentiated cell types. For each cell type, we computed the correlation of the MPRA-derived cCRE activity (MAD score) with the SCREEN DNase *z*-score per cCRE. DNase *z*-scores are normalized DNase accessibility signals, which are comparable across cell types. If several SCREEN elements overlapped a cCRE, we used the cCRE with the highest *z*-score. We repeated the analysis twice: once for all active chromatin types (promoter-like signature (PLS), chromatin-accessible (CA)-H3K4me3, proximal enhancer-like signature (pELS) and distal enhancer-like signature (dELS)) and once for enhancers only (pELS and dELS) (Supplementary Table 3).

#### Comparing cellular *trans* environments

Whereas the human allele was tested in its native human *trans* environment, the human–chimpanzee ancestral allele was assayed in a human rather than in a human–chimpanzee ancestral *trans* environment. For an MPRA sequence to exhibit differential behaviour across *trans* environments, the interacting *trans* factors must differ—either in their protein sequence (affecting binding affinity) or in their expression levels. We therefore sought to assess these sources of potential divergence.

First, we examined evolutionary changes in TF coding sequences that might alter DNA-binding specificity. We used a previous study^119^ that identified human-specific variants that are predicted to affect TF DNA-binding domains. Notably, only three TFs (ZFAT, ZFHX4 and ZNF18) were implicated. Of these, ZNF18 is not expressed in chondrocytes (TPM < 1). Using FIMO^40^, we tested motifs for ZFAT and ZFHX4 within our test sequences and found that none of these TFs is predicted to bind to them (FDR > 0.05). This suggests that coding changes between humans and chimpanzees in TF DNA-binding domains are unlikely to make a major contribution to our results.

Next, we evaluated differences in TF expression levels between humans and chimpanzees. Notably, human-versus-chimpanzee divergence overestimates human-versus-ancestor divergence, because $\mathop{5}\limits^{ \sim }$0% of human-versus-chimpanzee changes are chimpanzee-specific and thus do not contribute to differences between humans and their ancestors. Because expression data from human and chimpanzee chondrocytes are, to our knowledge, unavailable, we analysed cranial neural crest cells (CNCCs)^25^, which give rise to the facial skeleton. Across TF-encoding genes (*n* = 736, as defined by JASPAR^97^), we observed high correlations between species (Pearson’s *r* = 0.90, *P* = 1.8 × 10^−261^, Spearman’s *ρ* = 0.90, *P* = 5.2 × 10^−272^), indicating broadly conserved TF abundance across these skeletal contexts.

Because differences in measurements of TF expression between these human and chimp cells are driven not only by genetically encoded changes, but also by technical factors (for example, experimental noise), we turned to investigating TF expression in the human–ape hybrid osteochondral progenitors, which provide a more tightly controlled system for assessing genetically encoded *cis*-regulatory differences that affect TF genes. We found that TF-encoding genes show a high correlation in these cells as well (Pearson’s *r* = 0.94, *P* = 1.1 × 10^−276^.

Finally, we also compared gene expression profiles between human chondrocytes and human osteochondral progenitors, and observed high overall concordance (Pearson’s *r* = 0.86, *P* = 2.2 × 10^−308^), further supporting the notion that closely related skeletal cell types share a similar *trans* environment.

Although these results suggest that most TFs are likely to have similar levels in human and human–chimp ancestor cells, there are nevertheless some differences. Previous studies suggest that such variation affects mainly the magnitude rather than the direction of allelic effects. For example, in our MPRA of modern-human-derived variants in osteoblasts and neural progenitor cells, all allelic pairs (100%) showed concordant directionality of effect across the two cellular contexts^12^. To further examine this, we analysed our positive control sequences, previously assayed in osteoblasts and here in chondrocytes, and found that 89.52% retained the same direction of differential activity across cell types, and also exhibited a strong correlation in effect size (Pearson’s *r* = 0.64, *P* = 2.2 × 10^−23^).

Overall, although the precise contribution of *trans* differences between a human cell and an ancestral cell to differential activity is difficult to quantify, our analyses suggest that this contribution is limited, particularly with respect to the directionality of allelic effects.

#### GWAS catalogue analysis

To test the overlap of differentially active cCREs with skeletal-related loci, we first generated a list of skeletal-related phenotypes from the GWAS Catalog^120^ using the following set of keywords and regular expressions: musculoskeletal; skelet(al|on); bone(s)?; cartilage; cartilaginous; ossif.; joint(s)?; arthritis; osteoarthritis; rheumatoid; osteoporosis; osteopenia; fracture(s)?; scoliosis; kyphosis; lordosis; osteonecrosis; osteomyelitis; osteomalacia; osteopetrosis; osteogenesis imperfecta; rickets; spondyl.; ankylos.; gout(y)?; Paget; achondroplasia; chondrodysplasia; chondrocalcinosis; osteochondr.; osteophyte(s)?; intervertebral; disc degeneration; hallux valgus; bunion; osteosarcoma; spine; spinal; vertebra(e|l)?; hip(s)?; knee(s)?; ankle(s)?; elbow(s)?; shoulder(s)?; wrist(s)?; hand(s)?; foot; feet; femur; femoral; tibia; tibial; fibula; fibular; humerus; humeral; radius; ulna; ulnar; pelvis; pelvic; craniofacial; mandible; mandibular; maxilla; maxillary; carpal; tarsal; metacarpal; metatarsal; phalange.*; calcaneus; rib(s)?; bone mineral density; BMD; eBMD; bone area; bone geometry; trabecular; cortical (bone|thickness); heel bone; and quantitative ultrasound. Occurrences of joint(s)? were excluded when followed by statistical-context terms, including analysis, analyses, test, model, modeling, modelling, meta, effect, effects, distribution, study or studies. We then defined 1-kb windows centred on GWAS (genome-wide association study) variants associated with these terms, and tested whether human-derived variants in differentially active cCREs were enriched in these regions relative to other variants in our MPRA library. We found that human-derived variants in differentially active cCREs showed a significant enrichment in skeletal GWAS regions (Fisher’s exact test, *P* = 0.02).

### Generation of human–gorilla composite cell lines (hybrid cells)

#### Ethics statement

Approval for the derivation of human iPS cell lines used in this study was granted by the University of Chicago institutional review board (IRB) under protocol 11-0524. The human donors in this study consented to the use of their cells (fibroblasts) to generate iPS cells for studies of evolution and cross-species comparisons, and to the generation of other cell types that would be derived from these iPS cells. Donors consented to the deposition of any resulting data from the study in the NCBI GEO. The generation of human–ape composite iPS cells was approved by the Weizmann IRB under protocol 1586-2.

We note that these tetraploid cells are not approved for use in vivo or for attempting to generate an organism (which, biologically, is likely to be impossible). We recommend that all future applications of these cells occur in close consultation with bioethicists.

#### Generation and characterization of gorilla iPS cells

Gorilla iPS cells were generated by the CRYOZOO biobank of animal cell lines (UPF, EMBL Barcelona, Barcelona Zoo and MCNB) from primary skin fibroblasts using a Sendai virus-based reprogramming approach (CytoTune -iPS 2.0 Sendai Reprogramming Kit, Invitrogen, A16517). Fibroblasts at passage 2 were seeded 2 days before transduction at a density of 5 × 10^4^ cells per well in six-well plates coated with growth-factor-reduced Matrigel (80 μg ml^−1^) and cultured in αMEM supplemented with 10% fetal bovine serum (FBS), 1% penicillin–streptomycin and 1% GlutaMAX. On day 0, cells were transduced with Sendai viral vectors encoding KOS (human KLF4, human OCT4 and human SOX2), human MYC and human KLF4 at an MOI of 5:5:3, following the manufacturer’s instructions with minor modifications.

After transduction, cultures were fed daily with fresh αMEM-based fibroblast medium until day 6. On day 7, cells were passaged at a 1:2 ratio onto Matrigel-coated plates and maintained in αMEM-based medium. Cultures were progressively transitioned from fibroblast medium to mTeSR1, first using a 3:1 fibroblast medium ratio and subsequently to complete mTeSR1 once iPS cell colonies became morphologically distinct. Well-defined iPS cell colonies emerged after approximately 2 weeks in complete mTeSR1. Individual colonies were expanded and maintained on Geltrex (0.16 μg ml^−1^) in StemMACSiPS-Brew XF medium supplemented with iWR-1 (0.5 μM) and CHIR99021 (1 μM). For cryopreservation, iPS cell cultures at approximately 80% confluence were frozen in FBS containing 10% dimethyl sulfoxide (DMSO).

The pluripotent state of the gorilla iPS cell line GiPSC16 was assessed by immunocytochemistry using the StemLight Pluripotency IF Antibody Sampler Kit (Cell Signaling Technology, 9656). Expression of the pluripotency-associated markers OCT4, SSEA4, NANOG, TRA-1-60 and SOX2 was evaluated using the corresponding primary antibodies at a 1:200 dilution. Cells were washed twice with PBS and fixed with 4% formaldehyde for 20 min at room temperature. For detection of intracellular and nuclear antigens, cells were permeabilized with 0.1% Triton X-100 in PBS for 10 min and washed three times with PBS. Non-specific binding was blocked with 1% bovine serum albumin (BSA) and 0.1% Triton X-100 in PBS for 30 min. Cells were incubated with primary antibodies diluted in blocking buffer for 1 h at room temperature or overnight at 4 °C. After three washes with PBS, cells were incubated with the appropriate species-specific Alexa Fluor-conjugated secondary antibodies (Abcam) at a 1:200 dilution for 2 h at room temperature in the dark. Anti-rabbit IgG secondary antibodies were used for OCT4, NANOG and SOX2, whereas the corresponding anti-mouse secondary antibodies were used for SSEA4 and TRA-1-60. Cells were subsequently washed three times with PBS, and nuclei were counterstained with DAPI (0.1–1 μg ml^−1^) for 1 min.

Functional pluripotency was further assessed by directed differentiation of GiPSC16 into derivatives of the three embryonic germ layers using the Human Pluripotent Stem Cell Functional Identification Kit (R&D Systems, SC027B), according to the manufacturer’s instructions. Lineage identity was evaluated by immunocytochemical detection of SOX17 (endoderm), OTX2 (ectoderm) and brachyury (mesoderm), using the primary antibodies supplied with the kit at a final concentration of 10 μg ml^−1^. After incubation with primary antibodies, cells were incubated with the corresponding Alexa Fluor-conjugated anti-goat IgG secondary antibodies (Abcam) at a 1:200 dilution for 2 h at room temperature in the dark. Nuclei were counterstained with DAPI. Fluorescence images were acquired using a Leica TCS SP8 confocal laser-scanning microscope.

#### Cell fusion

Before cell fusion, 4 to 5 × 10^6^ human (H21792)^121^ and gorilla iPS cell lines were thawed in 2 ml Dulbecco’s PBS (DPBS; Sartorius, 020201A) supplemented with 5% FBS (Gioco, 12070106). Cells were centrifuged at 1,000 rpm for 5 min, the supernatant was aspirated and pellets were resuspended in 10 ml mTeSR1 Plus medium (STEMCELL Technologies, 100-0274) supplemented with 10 μM ROCK inhibitor Y-27632 (Tocris, 1254/10) and 50 μl penicillin streptomycin (2,500 U; Gibco, 15070063). Cells were seeded onto 10-cm plates coated with Matrigel (Corning, 354277; 1:100 in DMEM/F-12, Diagnobum, D814). The medium was changed after 2 days. On day 3, cells were dissociated with accutase (Sigma, A6964) for 2 min at 37 °C, and dissociation was stopped with DPBS containing 5% FBS. Cells from each line were transferred to separate 15-ml conical tubes and centrifuged at 1,000 rpm for 5 min. The supernatant was aspirated, and cell pellets were resuspended in 10 ml mTeSR1 Plus medium supplemented with 10 μM ROCK inhibitor Y-27632. Cells were counted using an automated cell counter (RWD, C100-SE) and distributed into five 15-ml conical tubes as follows: six million human iPS cells; six million gorilla iPS cells; one million human iPS cells; one million gorilla iPS cells; and a mixed population containing 0.5 million human iPS cells and 0.5 million gorilla iPS cells. Cell labelling and fusion were performed as previously described^24^, with modifications detailed below. Cells were centrifuged, the medium was aspirated and pellets were resuspended in 2 ml dye solution as follows: human iPS cells were labelled with CellTracker Deep Red (1.5 μM in DPBS; Invitrogen, C34565), gorilla iPS cells were labelled with CellTracker Green CMFDA (5 μM in DPBS; Invitrogen, C7025) and the mixed-cell sample was resuspended in DPBS containing DMSO (1:1,000) only. Tubes were incubated for 30 min at 37 °C with resuspension every 10 min, followed by centrifugation. Dye solutions were aspirated, and cells were washed three times with DPBS. Cells were then plated onto two Matrigel-coated 6-well plates (fusion and control) in 3 ml mTeSR1 Plus medium supplemented with 10 μM ROCK inhibitor Y-27632. Each well of the fusion plate contained one million human iPS cells and one million gorilla iPS cells. The control (no-fusion) plate included one well with unlabelled mixed cells (0.5 million human iPS cells and 0.5 million gorilla iPS cells), one well with one million gorilla iPS cells only, one well with one million human iPS cells only and one well with labelled mixed cells (0.5 million of each). Plates were incubated overnight at 37 °C. Fusion was performed the following day. Medium was aspirated from each well of the fusion plate, and cells were washed twice with DPBS. Polyethylene glycol 1500 (PEG) was added to each well (1 ml per well) and incubated at 37 °C for 2 min. PEG was aspirated, and cells were washed three times with mTeSR1 Plus medium, after which 4 ml mTeSR1 Plus medium supplemented with 10 μM ROCK inhibitor Y-27632 was added to each well. A day after fusion, medium was changed for all the cells (mTeSR1 Plus medium with 5 μM ROCK) and four Matrigel-coated 10-cm plates were prepared. The following day, cells were dissociated with accutase as described above. After centrifugation and removal of DPBS and accutase, cells were resuspended in sorting buffer by pipetting using a P1000 pipette, as described previously^25^. Cells were then placed on ice before sorting. Cells positive for both Deep Red and Green CMFDA, and negative for DAPI, were sorted using a FACSAria cell sorter into 15-ml tubes containing 2 ml ice-cold DPBS supplemented with 5% FBS (Supplementary Fig. 1). Cells were centrifuged at 1,000 rpm for 5 min, DPBS was aspirated and cells were resuspended in mTeSR1 Plus medium with 10 μM ROCK inhibitor Y-27632 and 2,500 U penicillin–streptomycin. Cells were plated on the prepared Matrigel plates at a density of 10,000 cells per plate. For the following five days, culture medium was supplemented with 5 μM ROCK inhibitor and replaced every two days until colonies became clearly visible. Colonies were picked and transferred, one colony per well of a 12-well Matrigel-coated plate. Each picked colony was assigned a unique identifier (1–96). The medium was replaced every two days, and once colonies reached a sufficient size, each line was passaged into a single well of a Matrigel-coated six-well plate. Cells were maintained under feeder-free conditions until reaching approximately 90% confluency. Each human–gorilla composite cell line was then dissociated using 300 μl accutase, as described above. After centrifugation, cells were resuspended in mTeSR1 Plus medium supplemented with 10 μM ROCK inhibitor Y-27632 and 2,500 U penicillin–streptomycin. A total of 0.5–1 million cells from each line were collected for PCR screening of potential composite cell lines, and the remaining cells were seeded onto pre-prepared 10-cm plates. DNA was extracted from each cell line using the Monarch Genomic DNA Purification Kit (NEB, T3010), according to the manufacturer’s instructions. PCR was performed for each cell line using AR primers specific to human (1,674 bp) and gorilla (536 bp) amplicons. Then, 160 ng genomic DNA from each sample was amplified in a 20-μl reaction containing GoTaq Green Master Mix (Promega, M7122) using a MiniAmp Plus thermal cycler (Thermo Fisher Scientific). Karyotyping was performed on all human–gorilla composite cell lines exhibiting both human and gorilla PCR bands by G-banding, following standard procedures^122^.

### Differentiation into osteochondral progenitor cells

Differentiation of human–gorilla and human–chimpanzee composite iPS cells into limb osteochondral progenitor cells was performed as described^123,124^, with minor modifications. In brief, iPS cells were counted and seeded at 9,000 cells per well in triplicate onto Matrigel-coated 24-well plates (10 μl ml^−1^ in DMEM, at least 1 h at 37 °C) and cultured for 24 h in mTeSR1 (STEMCELL Technologies, 85850) supplemented with Y-27632 (Tocris, 1254, 10 μM) and penicillin–streptomycin (25 IU ml^−1^). Medium was replaced with mTeSR1 containing penicillin–streptomycin without Y-27632, and cells were cultured for an additional 48 h. After DPBS washing, cells were cultured for 24 h in mid-primitive streak (MPS) medium prepared as described^123,124^. To accommodate rapid cellular expansion, an additional split point was introduced at this stage. Cells were dissociated by accutase (BioLegend, 423201) and replated onto Matrigel-coated 12-well plates in lateral plate mesoderm (LPM) medium, followed by 24 h of culture. Cells were washed with DPBS, the medium was replaced with limb bud mesenchyme (LBM) medium and cells were cultured for 48 h. LBM cells were washed with DPBS and dissociated with accutase, and viable cells were counted. A total of 90,000 cells per well were seeded onto fibronectin-coated (R&D Systems 1918-FN, 4 μg ml^−1^ in DPBS, 1 h at 37 °C) six-well plates. The medium was replaced after 48 h, and cells were collected 24 h later and snap-frozen in liquid nitrogen, to be used for RNA extraction.

#### RNA extraction and sequencing

RNA was extracted by detaching the entire well using accutase, followed by centrifugation at 200*g* for 5 min. The supernatant was aspirated, and the resulting cell pellet was immediately plunged into liquid nitrogen to prevent RNA degradation. The RNeasy Mini Kit (QIAGEN, 74104) was used according to the manufacturer’s instructions. The RNase-Free DNase Set (QIAGEN, 79254) was applied to the membrane during RNA extraction according to the manufacturer’s instructions. RNA purity and concentration were assessed using a NanoDrop spectrophotometer. Three samples out of ten underwent an additional clean-up step following the manufacturer’s instructions. RNA concentration was quantified using the Qubit RNA Assay Kit (Qubit RNA BR Assay kit, Q10210) and measured with a Qubit 3 Fluorometer. RNA integrity was assessed using the Agilent 2200 TapeStation System by an external service provider. Two technical replicates from each sample were submitted for RNA-sequencing library preparation using TruSeq Stranded mRNA, following the manufacturer’s instructions. Library preparation was performed by the Crown Genomics institute of the Nancy and Stephen Grand Israel National Center for Personalized Medicine, and sequencing was performed on an Illumina (NovaSeq X Plus 1.5B) platform, generating approximately 160 million paired-end reads per sample (2 × 150 bp).

For clarity, we use the term osteochondral progenitor cells and not expandable limb bud mesenchyme^123^, to provide a biological rather than a technical interpretation for these cells’ identity, as previously described^123,125^. We further validated this identity by a principal component analysis (PCA) (Extended Data Fig. 6b), and by confirming expression of the osteochondral markers *SOX9*, *PRRX1* and absence of *NANOG* expression (Supplementary Table 8), as described^125^. Data have been deposited in the NCBI GEO under accession number GSE316892.

### Identification of gene expression differences

We used the allele-specific expression pipeline adapted from a previous study^25^. The whole pipeline was done twice independently using human (GRCh38) and chimpanzee (panTro6) reference genomes for human–chimpanzee hybrids, and human (GRCh38) and gorilla (gorGor6) reference genomes for human–gorilla hybrids. The alignments were performed using STAR (v.2.7.11b) with arguments: -outSAMattributes MD NH -outFilterMultimapNmax 1 -sjdbGTFfile -sjdbOverhang 149. Two-pass mappings were performed (with the option –sjdbFileChrStartEnd to specify the splice junctions identified in the first round of mapping) to improve alignment accuracy. Duplicate reads were removed using Picard v.2.18.27 with the argument DUPLICATE_SCORING_STRATEGY = RANDOM. The set of single-nucleotide variants used to assign reads to either the human or chimpanzee genome was generated as previously described^27^. The human–gorilla single-nucleotide variant set was constructed using a similar approach, excluding filtering for single-nucleotide variants identified as homozygous in the human and gorilla parental lines. To minimize potential allelic imbalance biases when aligning one species to the genome of another species, we used a modified version of WASP^126^ (https://github.com/TheFraserLab/Hornet). In this pipeline, only reads that are mapped to the same position after in silico allele swapping are kept, thus ensuring that the variants in themselves do not create biased read mappability. Reads overlapping indels were also discarded. Read count data have been deposited in the NCBI GEO under accession number GSE316892.

To compute differential gene expression between human and chimpanzee, and between human and gorilla, we used DESeq2 (ref. ^127^). We used the likelihood ratio test and the model cond_Cell+cond_Species, in which cond_Cell represents the replicates and cond_Species represents the species. The analysis was done twice, first with counts derived from alignment to the GRCh38 reference genome, and then with counts derived from alignment to the panTro6 or gorGor6 genome. *P* values were adjusted for multiple testing using the Benjamini–Hochberg false discovery rate. Log_2_-transformed fold change (log_2_FC) estimates of human versus ape were shrunk following the recommendations of the DESeq2 workflow^128^. A gene was classified as differentially expressed between the pair of species only if it met all of the following criteria: (i) it was annotated in both genomes; (ii) it showed significant differential expression when reads were aligned to the human reference, and again when they were aligned to the non-human ape reference genome; and (iii) the log_2_FC values of differential expression when aligned to the human and non-human ape reference genome were in the same direction and differed by no more than 1.

In the osteochondral progenitor human–chimpanzee composite cell lines, differentially expressed genes were classified as human-derived if the gorilla allele expression level (TPM) in the osteochondral human–gorilla hybrid cells was closer to the chimpanzee TPM than to the human TPM. Conversely, genes were classified as chimpanzee-derived if the gorilla TPM was closer to the human TPM than the chimpanzee TPM (Fig. 2d). For this purpose, we did not require the human–gorilla difference to be significant.

#### Detection of aneuploidy

**Extended Data Fig. 11:**
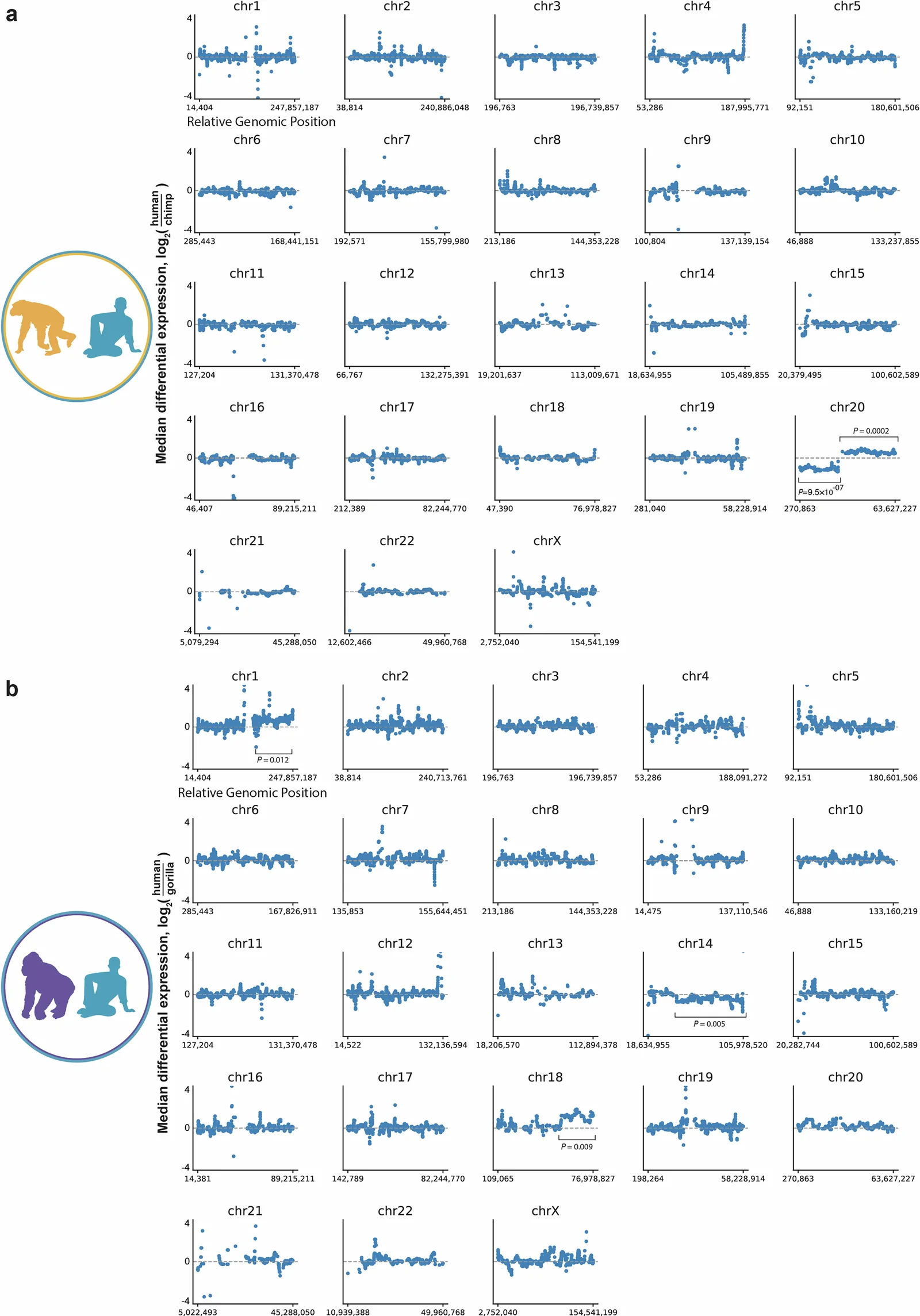
Aneuploidy test for each chromosome in the human–chimpanzee and human–gorilla hybrid limb osteochondral progenitor cells. **a**, Median differential expression (human versus chimpanzee) in 20-gene bins along each chromosome. **b**, Same for human versus gorilla. Bonferroni-adjusted two-sided Wilcoxon rank sum test *P* values are shown for significant bins.

To identify potential aneuploidy, we tested for species-biased stretches of deviations of the log_2_FC values along each chromosome using a Mann–Whitney *U*-test. In the osteochondral progenitor human–chimpanzee hybrid cells, we found a bias towards the human allele in the long arm of chromosome 20 and a bias towards the chimpanzee allele in the short arm (Extended Data Fig. 11a). For osteochondral progenitor human–gorilla cells, we found a bias towards the human allele in part of chromosome 18 (chr. 18: 43276708–73564699, GRCh38) for both cell lines (HG1 and HG2) and in part of chromosome 1 (chr.1: 150000000–248956422) for cell line HG1. In addition, there was a bias towards the gorilla allele in part of chromosome 14 (chr. 14: 50000000–107043718) in both cell lines (Extended Data Fig. 11b). We therefore removed these sections from all downstream calculations (TPM and differential expression).

#### Differentiation PCA

PCA was performed on the combination of a previously published dataset (Yamada et al.^123^) and our osteochondral progenitor human–chimpanzee and human–gorilla hybrid datasets. Genes with TPM > 1 in at least two samples were used. TPM values were transformed using log_2_(TPM + 1), and the 1,000 most variable genes in the Yamada et al. samples were selected. To mitigate technical differences between datasets, samples were assigned to batches according to their identifiers (Yamada, human–chimpanzee hybrids or human–gorilla hybrids), and batch effects were regressed out on a per-gene basis using a linear model with batch as a categorical covariate. The resulting residual expression values were then *z*-scored for each gene across samples using scaling parameters learned from the Yamada et al. dataset. PCA was subsequently fitted on the standardized Yamada et al. expression matrix, and the remaining samples were projected onto the same PCA space.

### *ACAN* GAG anchor repeat analysis

To determine the number of repeat units in *ACAN*, the repeat unit sequence defined as ‘repeat type 2’ in a previous report^45^ (GGGCTTCCTTCTGGAGAAGTTCTAGAGACCGCTGCCCCTGGAGTAGAGGACATCAGC) was aligned to 342 long-read de-novo-assembled haploid or diploid genome builds from 158 humans (316 haplotypes)^129,130^ (Supplementary Table 14) and 20 non-human great apes (26 haplotypes)^131–134 ^(Supplementary Table 13). Alignments were performed using BLAST+ (v.2.14.0)^135^ with the following parameters: ‘-task blastn -word_size 7 -evalue 1e-1 -perc_identity 70 -qcov_hsp_perc 80’. In cases in which a genome build was unavailable or when the *ACAN* locus was poorly assembled, the raw long-read sequences were used instead^136,137^.

Alignment matches were inspected manually, because occasional misclassification of DNA segments led to missed repeat calls. Thus, the number of repeat units was corrected by dividing the genomic span of the detected repeat region by the repeat unit length (57 bp), calculated as the position of the most downstream match minus the position of the most upstream match.

#### Partitioning of human repeat-count distribution

To characterize heterogeneity in the distribution of repeat counts across human haplotypes, we applied model-based clustering using the Mclust function from the mclust R package (v.6.0.0)^138^. The distribution of repeat counts was modelled as a finite mixture of Gaussian components, with the number of components *G* ranging from 1 to 5. Model selection was performed using the Bayesian information criterion (BIC), and the model with the highest BIC was selected (*G* = 2). For each haplotype, model-based posterior classification probabilities were computed, and haplotypes were assigned to the component with the highest posterior probability.

### Comparison of GAG composition between humans and non-human great apes

#### Ethics statement

All tissues were collected post-mortem (Extended Data Figs. 8 and 9 and Supplementary Table 15). No animals were killed for the purpose of this study. Ape specimens were obtained opportunistically after death. Chimpanzee hand samples were obtained from a 60-year-old female individual, who died of natural causes at the Zoological Center in Ramat Gan, Israel on 27 April 2024). Hand specimens were stored at −80 °C and thawed before tissue collection. All other ape specimens were obtained through collaboration with European zoos (call through the European Association of Zoos and Aquaria). All procedures were performed in accordance with approval number M011/2026 from the KU Leuven Ethical Committee of Animal Experimentation.

Formalin-fixed human specimens were obtained from the Farkas Family Center for Anatomical Research and Education (CARE), Rappaport Faculty of Medicine, Technion—Israel Institute of Technology. All procedures conformed to the ethical guidelines of the Technion and the Israeli Ministry of Health. Informed consent was obtained from all body donors, explicitly permitting the use of donated tissues for anatomical education and research.

#### Sample collection

Hand articular cartilage: articular cartilage samples were collected from human and chimpanzee specimens from the following hand joints: the first metacarpophalangeal joint (MCP; *n* = 14), the second–fourth MCP joint (*n* = 21), the proximal interphalangeal joint (PIP; *n* = 17) and the distal interphalangeal joint (DIP; *n* = 18). Full-thickness cartilage samples, measuring approximately 0.5–1 cm in maximal dimension depending on joint size and available articular surface, were collected from each site. For joint samples, full thickness was defined as extending from the articular surface to the underlying subchondral bone. A skin incision was made directly over each joint, followed by careful dissection and reflection of surrounding soft tissues to expose the joint capsule. When necessary, the fibrous digital flexor sheath was opened at the level of the annular pulleys to facilitate access to the articular cartilage.

Elbow articular cartilage (capitulum): the upper limb was positioned in supination. Skin and subcutaneous tissue over the cubital fossa were reflected to expose the distal arm and proximal forearm. The plane between the brachialis and brachioradialis muscles was dissected to gain access to the elbow joint. After identification of the humeral capitulum, the overlying articular cartilage was collected using a no. 10 scalpel blade.

Elbow articular cartilage (trochlea): the upper limb was positioned in supination. Skin and subcutaneous tissues were reflected over the cubital fossa to expose the distal arm and proximal forearm. The brachialis, pronator teres and common flexor tendon were identified and reflected to gain access to the medial aspect of the elbow joint. The medial epicondyle of the humerus was identified as an anatomical landmark, and the humeral trochlea was exposed immediately distal to it. The articular cartilage overlying the trochlea was then dissected using a no. 10 scalpel blade.

Knee: medial femoral condyle: the skin overlying the knee was reflected to expose the distal portions of the vastus medialis and vastus lateralis muscles, as well as the superior border of the patellar ligament. A transverse incision was then made at the level of the patellar ligament and extended superiorly along the margins of the vastus medialis and vastus lateralis for approximately 8–10 cm, circumferentially outlining the knee joint. This soft-tissue flap was reflected superiorly to expose the knee-joint capsule. The capsule was incised, and the leg was subsequently flexed to open the joint space and allow clear visualization of the femoral condyles. Articular cartilage was then collected from the medial femoral condyle using a no. 10 scalpel blade.

Knee: proximal tibia: the skin overlying the knee was reflected to expose the distal portions of the vastus medialis and vastus lateralis, as well as the superior border of the patellar ligament. A transverse incision was made at the level of the patellar ligament. The soft-tissue flap was reflected superiorly to expose the knee-joint capsule. After capsular incision and flexion of the leg, the proximal articular surface of the tibia was visualized. The menisci and tibial plateau were identified, and articular cartilage was collected from the medial tibial facet using a no. 10 scalpel blade.

Shoulder: humeral head: the donor body was positioned prone. The skin over the shoulder and proximal arm was reflected to expose the deltoid muscle and the proximal portion of the triceps brachii. These muscles were then reflected to gain access to the glenohumeral joint region. The humeral head was identified, and the overlying articular cartilage was collected using a no. 10 scalpel blade.

Wrist: scaphoid articular cartilage: the distal forearm was dissected to expose the flexor and extensor tendons of the radial aspect of the wrist. The tendons forming the anatomical snuff box—that is, the abductor pollicis longus, extensor pollicis brevis and extensor pollicis longus—were identified and reflected to expose the radioscaphoid joint. On the flexor aspect, the radial artery and the tendon of flexor carpi radialis were identified and reflected to improve visualization of the joint. The articular surface of the scaphoid was then identified, and the overlying cartilage was collected using a no. 10 scalpel blade.

Hip: femoral head: an anterior approach was used. The inguinal ligament was identified, and the soft tissues over the proximal anterolateral thigh were dissected from the level of the anterior superior iliac spine distally to expose the trochanteric region of the femur. The femoral head was then mobilized from the hip joint, and the overlying articular cartilage was collected using a no. 10 scalpel blade.

All samples were immersed in 1× PBS and stored at 4 °C.

#### DNA quantification

The DNA content of each sample was quantified using a Hoechst 33258 fluorescence assay. A dye buffer was prepared from 10 mM Tris base, 1 mM EDTA and 0.1 mM NaCl, adjusted to pH 7.4. Hoechst 33258 (Sigma, 94403) was prepared as a 1 mg ml^−1^ stock solution in distilled water and stored protected from light at 4 °C. Immediately before use, the dye was diluted in dye buffer to a final concentration of 0.1 µg ml^−1^.

Double-stranded DNA standards were prepared in PBS from a 50 µg ml^−1^ working stock to generate a standard curve ranging from 0 to 6 µg ml^−1^. Before dilution, the DNA standard (Sigma, D4522) was heated at 100 °C for 10 min. Papain-digested samples (see ‘Sample digestion and DMMB assay’) were diluted in PBS with a 1:25 dilution.

Standards and samples were loaded in triplicate into black 96-well plates at 10 μl per well. Hoechst dye solution was then added at 200 µl per well and incubated for 10 min. Fluorescence was measured using excitation at 350 nm and emission at 450 nm. DNA concentrations in the samples were calculated from the standard curve and corrected for dilution (Supplementary Table 20). Correlation between technical replicates was high (Pearson’s *r* = 0.97, *P* = 2.5 × 10^−7^ (Extended Data Fig. 10a). DNA content was correlated with sample weight (Pearson’s *r* = 0.70, *P* = 1.4 × 10^−21^ (Extended Data Fig. 10b).

#### DMMB preparation

The DMMB reagent was prepared by dissolving 16 mg DMMB (1,9-dimethylmethylene blue) in 1 l distilled water containing 3.04 g glycine and 2.37 g sodium chloride. The solution was stirred at room temperature protected from light, and the pH was adjusted to 3.0 using HCl before use^139^.

#### Sample digestion and DMMB assay

Samples were weighed, finely minced, and digested in 400 μl papain digestion buffer containing 40 μg ml^−1^ papain (prepared by diluting a 25 mg ml^−1^ papain stock; Sigma, P3125) at 65 °C for 48 h. After papain digestion, samples were diluted in 1% (w/v) BSA. sGAG content was quantified using the DMMB assay, with a standard curve generated from chondroitin sulfate sodium salt (Sigma, C8529). Ten microlitres of each sample or standard was loaded in triplicate into a transparent 96-well plate, followed by 200 μl DMMB reagent per well. Absorbance was measured immediately at 525 nm. Standard error per sample ranged between 0.02 and 5.94, with an average of 0.49. Triplicate measurements were averaged to generate a single value representing the sGAG content of each sample. Measurements were normalized separately by two factors: DNA content (see above) and weight. Normalized values for both methods are provided (Supplementary Table 17). The normalized sGAG content was strongly correlated between technical replicates (Extended Data Fig. 10c) and similar to the literature^140–143^.

#### Confounder analysis: sex

To test potential confounders, we analysed the effects of age and sex on normalized sGAG content. Sex was not a significant predictor of sGAG content in either humans or apes (Extended Data Fig. 10d). Similarly, no significant effect of sex was detected when each joint was analysed separately (*P* between 0.116 and 0.687 for joints with three samples or more) (Extended Data Fig. 10e).

#### Confounder analysis: age

We used two methods to correct ape ages for cross-species comparison: (i) division by maximum observed lifespan per species^144^ and (ii) a time-translation framework^145^. To this end, we generated a set of human-to-ape age conversion tables based on a previously published time-translation framework for developmental events^145^. Using the pipeline provided in the paper, we produced an output table in which each row is an event × species observation. Gestation days per species were taken from the AnAge Database (build 15)^144^. We converted the post-conception days (DD) values to years after birth using the following function:

$${\rm{A}}{\rm{g}}{\rm{e}}({\rm{y}}{\rm{e}}{\rm{a}}{\rm{r}}{\rm{s}})=\frac{{10}^{{\rm{D}}{\rm{D}}}-\mathrm{gestation}\_\mathrm{time}}{365}$$

The resulting event–age pairs were sorted and projected onto the closest monotonically non-decreasing curve using isotonic regression (sklearn.isotonic.IsotonicRegression), which removed minor non-monotonicities introduced by imputation noise without distorting the fit. A monotone interpolating curve was then fitted to the isotonic-corrected pairs using a piecewise cubic Hermite interpolating polynomial (PCHIP), with extrapolation disabled.

All species PCHIPs were evaluated on a shared dense grid of 2,000 values along Model_Event, restricted to the intersection of their observed event ranges. The resulting paired points (human_age and ape_age) were then used to fit a final per-species linear regression across the full valid range, by ordinary least squares (numpy.polyfit (degree 1)):

$$\mathrm{ape}\_\mathrm{age}\,=\,\alpha +\beta \times \mathrm{human}\_\mathrm{age}.$$

The linear regression enabled us to extrapolate values beyond the provided range (Extended Data Fig. 10f,g).

Similarly to sex, chronological age was not a significant predictor of GAG content in either humans or apes (Extended Data Fig. 10f). Furthermore, using both age-adjustment methods, ape samples consistently showed a higher GAG content than did their adjusted age-matched human counterparts (Extended Data Fig. 10h).

### Statistical analysis

#### Joint-level test

In cases in which an individual had more than one sample per joint, the mean of the samples was used. After this procedure, 74 data points remained (57 humans and 17 apes). For phalangeal subregions (PIP, MCP and DIP) with a single ape sample, parametric *z*-score tests were performed. For other joints (wrist, shoulder, hip, elbow and knee) with larger sample sizes, independent two-sided *t*-tests were used to compare human and ape values. *P* values were FDR-adjusted using the Benjamini–Hochberg method^92^.

#### Global (all-joint) test

Data points were further collapsed to retain a single value per individual. After this procedure, 49 data points remained (42 humans and 7 apes). A two-sided *t*-test was used to compare human and ape values.

### Reporting summary

Further information on research design is available in the Nature Portfolio Reporting Summary linked to this article.

## Online content

Any methods, additional references, Nature Portfolio reporting summaries, source data, extended data, supplementary information, acknowledgements, peer review information; details of author contributions and competing interests; and statements of data and code availability are available at https://doi.org/10.1038/s41586-026-11053-x.

## Supplementary information

Supplementary Information This file contains Supplementary Results, Supplementary Discussion and Supplementary Fig. 1.

Reporting Summary

Supplementary Tables Supplementary Tables 1–20

Peer Review File

## Data Availability

Data have been deposited in the NCBI GEO under accession numbers GSE316891 and GSE316892, and in the DDBJ database under accession number PRJDB40123. Publicly available datasets analysed in this study included gnomAD v.2.1.1 (GRCh37) and v.4 (GRCh38), ENCODE SCREEN (v.2; GRCh37), 1KG and HGDP (taken from gnomAD v.3.1; GRCh38), JASPAR2024, the HPO database (release 2024-04-26), IPA (v.159584291), ENCODE SCREEN (v.4; GRCh38), GWAS catalogue (all associations, v.1.0.2, downloaded 26 September 2021), AnAge (build 15) and the Human Pangenome Reference Consortium (HPRC) human assemblies (downloaded 17 February 2026). PBM data were downloaded from the NCBI GEO under accession number GSE53348.

## Code availability

All custom scripts used for preprocessing, statistical analyses and generating figures are available at GitHub: https://github.com/GokhmanLabOrganization/the_gene_regulatory_evolution_of_the_human_skeleton and https://github.com/GokhmanLabOrganization/differential-TF-binding. Processed data matrices required to reproduce the analyses are provided within the repository.

## References

[1] Aiello, L. & Dean, C. *An Introduction to Human Evolutionary Anatomy* (Academic Press, 1990).

[2] Housman, G. Advances in skeletal genomics research across tissues and cells. *Curr. Opin. Genet. Dev.* **88**, 102245 10.1016/j.gde.2024.102245 (2024). 39180931

[3] Housman, G. in *Evolutionary Cell Processes in Primates*, Vol. 2 (eds Pitirri, M. K. & Richtsmeier, J. T.) Ch. 4 (CRC Press, 2021).

[4] Jurmain, R. Degenerative joint disease in African great apes: an evolutionary perspective. *J. Hum. Evol.* **39**, 185–203 10.1006/jhev.2000.0413 (2000). 10968928

[5] Poe, D. J. *The Prevalence of Osteoarthritis in Wild vs Captive Great Ape Skeletons*. PhD thesis, Univ. New Mexico (2009).

[6] King, M. C. & Wilson, A. C. Evolution at two levels in humans and chimpanzees. *Science* **188**, 107–116 10.1126/science.1090005 (1975). 1090005

[7] Fraser, H. B. Gene expression drives local adaptation in humans. *Genome Res.* **23**, 1089–1096 10.1101/gr.152710.112 (2013). PMC3698502 23539138

[8] Wittkopp, P. J. & Kalay, G. *Cis*-regulatory elements: molecular mechanisms and evolutionary processes underlying divergence. *Nat. Rev. Genet.* **13**, 59–69 10.1038/nrg3095 (2011). 22143240

[9] Cotney, J. et al. The evolution of lineage-specific regulatory activities in the human embryonic limb. *Cell* **154**, 185–196 10.1016/j.cell.2013.05.056 (2013). PMC3785101 23827682

[10] Easterlin, R. & Ahituv, N. Lineage-specific regulatory evolution: insights from massively parallel reporter assays. *Curr. Opin. Genet. Dev.* **93**, 102372 10.1016/j.gde.2025.102372 (2025). PMC13215266 40543151

[11] Gallego Romero, I. & Lea, A. J. Leveraging massively parallel reporter assays for evolutionary questions. *Genome Biol.* **24**, 26 10.1186/s13059-023-02856-6 (2023). PMC9926830 36788564

[12] Weiss, C. V. et al. The *cis*-regulatory effects of modern human-specific variants. *eLife* **10**, e63713 10.7554/eLife.63713 (2021). PMC8062137 33885362

[13] Okamoto, A. S., Coveney, C. R., Ganapathee, D. S. & Capellini, T. D. In vitro massively parallel screening of human regulatory elements involved in postcranial skeletal development for differential activity compared to chimpanzee. *Genome Biol. Evol.* **18**, evag121 10.1093/gbe/evag121 (2026). PMC13198024 42119152

[14] Pavlovic, B. J., Fox, D., Schaefer, N. K. & Pollen, A. A. Rethinking nomenclature for interspecies cell fusions. *Nat. Rev. Genet.* **23**, 315–320 10.1038/s41576-021-00447-4 (2022). 35082442

[15] Kuderna, L. F. K. et al. A global catalog of whole-genome diversity from 233 primate species. *Science* **380**, 906–913 10.1126/science.abn7829 (2023). PMC12120848 37262161

[16] Karczewski, K. J. et al. The mutational constraint spectrum quantified from variation in 141,456 humans. *Nature* **581**, 434–443 10.1038/s41586-020-2308-7 (2020). PMC7334197 32461654

[17] Mafessoni, F. et al. A high-coverage Neandertal genome from Chagyrskaya Cave. *Proc. Natl Acad. Sci. USA* **117**, 15132–15136 10.1073/pnas.2004944117 (2020). PMC7334501 32546518

[18] Prüfer, K. et al. The complete genome sequence of a Neanderthal from the Altai Mountains. *Nature* **505**, 43–49 10.1038/nature12886 (2014). PMC4031459 24352235

[19] Prüfer, K. et al. A high-coverage Neandertal genome from Vindija Cave in Croatia. *Science* **358**, 655–658 10.1126/science.aao1887 (2017). PMC6185897 28982794

[20] Meyer, M. et al. A high-coverage genome sequence from an archaic Denisovan individual. *Science* **338**, 222–226 10.1126/science.1224344 (2012). PMC3617501 22936568

[21] Moore, J. E. et al. Expanded encyclopaedias of DNA elements in the human and mouse genomes. *Nature* **583**, 699–710 10.1038/s41586-020-2493-4 (2020). PMC7410828 32728249

[22] Ashuach, T. et al. MPRAnalyze: statistical framework for massively parallel reporter assays. *Genome Biol.* **20**, 183 10.1186/s13059-019-1787-z (2019). PMC6717970 31477158

[23] Fishilevich S. et. al. *Quality Control Pipeline for Massively Parallel Reporter Assays (MPRAs)* https://gokhmanlaborganization.github.io/MPRA_QC_book/ (2026).

[24] Agoglia, R. M. et al. Primate cell fusion disentangles gene regulatory divergence in neurodevelopment. *Nature* **592**, 421–427 10.1038/s41586-021-03343-3 (2021). PMC8719633 33731928

[25] Gokhman, D. et al. Human–chimpanzee fused cells reveal *cis*-regulatory divergence underlying skeletal evolution. *Nat. Genet.* **53**, 467–476 10.1038/s41588-021-00804-3 (2021). PMC8038968 33731941

[26] Song, J. H. T. et al. Genetic studies of human-chimpanzee divergence using stem cell fusions. *Proc. Natl Acad. Sci. USA* **118**, e2117557118 10.1073/pnas.2117557118 (2021). PMC8713981 34921118

[27] Wang, B., Starr, A. L. & Fraser, H. B. Cell-type-specific *cis*-regulatory divergence in gene expression and chromatin accessibility revealed by human-chimpanzee hybrid cells. *eLife* **12**, RP89594 10.7554/eLife.89594 (2024). PMC10942608 38358392

[28] Song J. H. T. et al. Human–chimpanzee tetraploid system defines mechanisms of species-specific neural gene regulation. Preprint at *bioRxiv* 10.1101/2025.03.31.646367 (2025).

[29] Carter A. C. et al. FOS binding sites are a hub for the evolution of activity-dependent gene regulatory programs in human neurons. Preprint at *bioRxiv* 10.1101/2025.03.31.646366 (2025).

[30] Schmitz, D. A. et al. Unraveling mitochondrial influence on mammalian pluripotency via enforced mitophagy. *Cell* **188**, 4773–4789 10.1016/j.cell.2025.05.020 (2025). PMC12354063 40499542

[31] Fishilevich, S. et al. GeneHancer: genome-wide integration of enhancers and target genes in GeneCards. *Database* **2017**, bax028 10.1093/database/bax028 (2017). PMC5467550 28605766

[32] Krämer, A., Green, J., Pollard, J. & Tugendreich, S. Causal analysis approaches in Ingenuity Pathway Analysis. *Bioinformatics* **30**, 523–530, 10.1093/bioinformatics/btt703 (2014). PMC3928520 24336805

[33] Aleksander, S. A. et al. The Gene Ontology knowledgebase in 2023. *Genetics* **224**, iyad031 10.1093/genetics/iyad031 (2023). PMC10158837 36866529

[34] Gargano, M. A. et al. The Human Phenotype Ontology in 2024: phenotypes around the world. *Nucleic Acids Res.* **52**, D1333–D1346 10.1093/nar/gkad1005 (2024). PMC10767975 37953324

[35] Becker, K. G., Barnes, K. C., Bright, T. J. & Wang, S. A. The Genetic Association Database. *Nat. Genet.* **36**, 431–432 10.1038/ng0504-431 (2004). 15118671

[36] Raikov, B. et al. Methods for determining the molecular composition of knee joint structures in osteoarthritis: collagen, proteoglycans and water content: a systematic review. *Collagen Leather* **6**, 30. 10.1186/s42825-024-00173-7 (2024).

[37] Kiani, C., Chen, L., Wu, Y. J., Yee, A. J. & Yang, B. B. Structure and function of aggrecan. *Cell Res.* **12**, 19–32 10.1038/sj.cr.7290106 (2002). 11942407

[38] Kanehisa, M. & Goto, S. KEGG: Kyoto Encyclopedia of Genes and Genomes. *Nucleic Acids Res.* **28**, 27–30 10.1093/nar/28.1.27 (2000). PMC102409 10592173

[39] Okamoto, A. et al. Modular genetic architecture underlies human hand and foot evolution. *Proc. Natl Acad. Sci. USA* **123**, e2603297123 10.1073/pnas.2603297123 (2026). PMC13187773 42118837

[40] Grant, C. E., Bailey, T. L. & Noble, W. S. FIMO: scanning for occurrences of a given motif. *Bioinformatics* **27**, 1017–1018 10.1093/bioinformatics/btr064 (2011). PMC3065696 21330290

[41] Housman, G., Briscoe, E. & Gilad, Y. Evolutionary insights into primate skeletal gene regulation using a comparative cell culture model. *PLoS Genet.* **18**, e1010073 10.1371/journal.pgen.1010073 (2022). PMC8936463 35263340

[42] Gokhman, D. et al. Reconstructing the DNA methylation maps of the Neandertal and the Denisovan. *Science* **344**, 523–527 10.1126/science.1250368 (2014). 24786081

[43] Gokhman, D. et al. Differential DNA methylation of vocal and facial anatomy genes in modern humans. *Nat. Commun.* **11**, 1189 10.1038/s41467-020-15020-6 (2020). PMC7055320 32132541

[44] Beyter, D. et al. Long-read sequencing of 3,622 Icelanders provides insight into the role of structural variants in human diseases and other traits. *Nat. Genet.* **53**, 779–786 10.1038/s41588-021-00865-4 (2021). 33972781

[45] Mukamel, R. E. et al. Protein-coding repeat polymorphisms strongly shape diverse human phenotypes. *Science* **373**, 1499–1505 10.1126/science.abg8289 (2021). PMC8549062 34554798

[46] Pajic, P. & Gokcumen, O. Evolutionary balancing of genetic consequence and innovation in mammals through variable number tandem repeats. *Genome Biol. Evol.* **18**, evaf250 10.1093/gbe/evaf250 (2026). PMC12776774 41437782

[47] Sulovari, A. et al. Human-specific tandem repeat expansion and differential gene expression during primate evolution. *Proc. Natl Acad. Sci. USA* **116**, 23243–23253 10.1073/pnas.1912175116 (2019). PMC6859368 31659027

[48] Sah, R. L.-Y. et al. Biosynthetic response of cartilage explants to dynamic compression. *J. Orthop. Res.* **7**, 619–636 10.1002/jor.1100070502 (1989). 2760736

[49] Bachrach, N. M. et al. Changes in proteoglycan synthesis of chondrocytes in articular cartilage are associated with the time-dependent changes in their mechanical environment. *J. Biomech.* **28**, 1561–1569 10.1016/0021-9290(95)00103-4 (1995). 8666595

[50] Larsson, T., Aspden, R. M. & Heinegård, D. Effects of mechanical load on cartilage matrix biosynthesis in vitro. *Matrix* **11**, 388–394 10.1016/s0934-8832(11)80193-9 (1991). 1787831

[51] Wareing, K. A. *Adaptation of the Non-Human Great Ape Lower Limb in Response to Locomotor Behaviour*. PhD thesis, Univ. Liverpool 10.17638/03001676 (2016).

[52] Yamada, S., Sugahara, K. & Özbek, S. Evolution of glycosaminoglycans: comparative biochemical study. *Commun. Integr. Biol.* **4**, 150–158 10.4161/cib.4.2.14547 (2011). PMC3104567 21655428

[53] Mishol, N. et al. Candidate Denisovan fossils identified through gene regulatory phenotyping. *Proc. Natl Acad. Sci. USA* **122**, e2513968122 10.1073/pnas.2513968122 (2025). PMC12415249 40857315

[54] Gokhman, D. et al. Reconstructing Denisovan anatomy using DNA methylation maps. *Cell* **179**, 180–192 10.1016/j.cell.2019.08.035 (2019). 31539495

[55] Gokhman, D., Harris, K. D., Carmi, S. & Greenbaum, G. Predicting the direction of phenotypic difference. *Nat. Commun.* **16**, 6898 10.1038/s41467-025-62355-z (2025). PMC12297712 40715205

[56] Silagi, E. S., Shapiro, I. M. & Risbud, M. V. Glycosaminoglycan synthesis in the nucleus pulposus: dysregulation and the pathogenesis of disc degeneration. *Matrix Biol.* **71–72**, 368–379 10.1016/j.matbio.2018.02.025 (2018). PMC6119535 29501510

[57] Paganini, C., Costantini, R., Superti-Furga, A. & Rossi, A. Bone and connective tissue disorders caused by defects in glycosaminoglycan biosynthesis: a panoramic view. *FEBS J.* **286**, 3008–3032 10.1111/febs.14984 (2019). 31286677

[58] Casal-Beiroa, P. et al. Optical biomarkers for the diagnosis of osteoarthritis through raman spectroscopy: radiological and biochemical validation using ex vivo human cartilage samples. *Diagnostics* **11**, 546 10.3390/diagnostics11030546 (2021). PMC8003208 33803917

[59] Shamdani, S. et al. Heparan sulfate functions are altered in the osteoarthritic cartilage. *Arthritis Res. Ther.* **22**, 283 10.1186/s13075-020-02352-3 (2020). PMC7722421 33287871

[60] Richard, D. et al. Evolutionary selection and constraint on human knee chondrocyte regulation impacts osteoarthritis risk. *Cell* **181**, 362–381 10.1016/j.cell.2020.02.057 (2020). PMC7179902 32220312

[61] Rivera, F. et al. Effectiveness of intra-articular injections of sodium hyaluronate-chondroitin sulfate in knee osteoarthritis: a multicenter prospective study. *J. Orthop. Traumatol.* **17**, 27–33 10.1007/s10195-015-0388-1 (2016). PMC4805636 26577936

[62] Kircher, M. et al. Saturation mutagenesis of twenty disease-associated regulatory elements at single base-pair resolution. *Nat. Commun.* **10**, 3583 10.1038/s41467-019-11526-w (2019). PMC6687891 31395865

[63] Khaitovich, P., Pääbo, S. & Weiss, G. Toward a neutral evolutionary model of gene expression. *Genetics* **170**, 929–939 10.1534/genetics.104.037135 (2005). PMC1450413 15834146

[64] Walsh, B. & Lynch, M.* Evolution and Selection of Quantitative Traits* (Oxford Univ. Press, 2018).

[65] Durinck, S., Spellman, P. T., Birney, E. & Huber, W. Mapping identifiers for the integration of genomic datasets with the R/Bioconductor package biomaRt. *Nat. Protoc.* **4**, 1184–1191 10.1038/nprot.2009.97 (2009). PMC3159387 19617889

[66] Schwartz, N. B. & Domowicz, M. S. Proteoglycans in brain development and pathogenesis. *FEBS Lett.* **592**, 3791–3805 10.1002/1873-3468.13026 (2018). 29513405

[67] Chou, H. H. et al. Inactivation of CMP-*N*-acetylneuraminic acid hydroxylase occurred prior to brain expansion during human evolution. *Proc. Natl Acad. Sci. USA* **99**, 11736–11741 10.1073/pnas.182257399 (2002). PMC129338 12192086

[68] Xue, Y. et al. Mountain gorilla genomes reveal the impact of long-term population decline and inbreeding. *Science* **348**, 242–245 10.1126/science.aaa3952 (2015). PMC4668944 25859046

[69] Prado-Martinez, J. et al. Great ape genetic diversity and population history. *Nature* **499**, 471–475 10.1038/nature12228 (2013). PMC3822165 23823723

[70] de Manuel, M. et al. Chimpanzee genomic diversity reveals ancient admixture with bonobos. *Science* **354**, 477–481 10.1126/science.aag2602 (2016). PMC5546212 27789843

[71] Nater, A. et al. Morphometric, behavioral, and genomic evidence for a new orangutan species. *Curr. Biol.* **27**, 3487–3498 10.1016/j.cub.2017.09.047 (2017). 29103940

[72] Gao, H. et al. The landscape of tolerated genetic variation in humans and primates. *Science* **380**, eabn8153 10.1126/science.abn8197 (2023). PMC10713091 37262156

[73] Smit, A., Hubley, R. & Green P. RepeatMasker Open-3.0 https://www.repeatmasker.org/ (1996).

[74] Benson, G. Tandem repeats finder: a program to analyze DNA sequences. *Nucleic Acids Res.* **27**, 573–580 10.1093/nar/27.2.573 (1999). PMC148217 9862982

[75] Karolchik, D. et al. The UCSC Table Browser data retrieval tool. *Nucleic Acids Res.* **32**, D493–D496 10.1093/nar/gkh103 (2004). PMC308837 14681465

[76] Amemiya, H. M., Kundaje, A. & Boyle, A. P. The ENCODE blacklist: identification of problematic regions of the genome. *Sci. Rep.* **9**, 9354 10.1038/s41598-019-45839-z (2019). PMC6597582 31249361

[77] Agarwal, V. et al. Massively parallel characterization of transcriptional regulatory elements. *Nature* **639**, 411–420 10.1038/s41586-024-08430-9 (2025). PMC11903340 39814889

[78] Sayers, E. W. et al. Database resources of the National Center for Biotechnology Information in 2025. *Nucleic Acids Res.* **53**, D20–D29 10.1093/nar/gkae979 (2025). PMC11701734 39526373

[79] Perez, G. et al. The UCSC Genome Browser database: 2025 update. *Nucleic Acids Res.* **53**, D1243–D1249 10.1093/nar/gkae974 (2025). PMC11701590 39460617

[80] Quinlan, A. R. BEDTools: the Swiss-Army tool for genome feature analysis. *Curr. Protoc. Bioinformatics* **47**, 11.12.1–11.12.34 10.1002/0471250953.bi1112s47 (2014). PMC4213956 25199790

[81] Danecek, P. et al. The variant call format and VCFtools. *Bioinformatics* **27**, 2156–2158 10.1093/bioinformatics/btr330 (2011). PMC3137218 21653522

[82] Danecek, P. et al. Twelve years of SAMtools and BCFtools. *GigaScience* **10**, giab008 10.1093/gigascience/giab008 (2021). PMC7931819 33590861

[83] Lee, B. T. et al. The UCSC Genome Browser database: 2022 update. *Nucleic Acids Res.* **50**, D1115–D1122 10.1093/nar/gkab959 (2022). PMC8728131 34718705

[84] Issack, P. S., Fang, C., Leslie, M. P. & Di Cesare, P. E. Chondrocyte-specific enhancer regions in the COMP gene. *J. Orthop. Res.* **18**, 345–350 10.1002/jor.1100180304 (2000). 10937619

[85] Kvon, E. Z. et al. Progressive loss of function in a limb enhancer during snake evolution. *Cell* **167**, 633–642 10.1016/j.cell.2016.09.028 (2016). PMC5484524 27768887

[86] Leung, V. Y. L. et al. SOX9 governs differentiation stage-specific gene expression in growth plate chondrocytes via direct concomitant transactivation and repression. *PLoS Genet.* **7**, e1002356 10.1371/journal.pgen.1002356 (2011). PMC3207907 22072985

[87] Jash, A., Yun, K., Sahoo, A., So, J. S. & Im, S. H. Looping mediated interaction between the promoter and 3′ UTR regulates type II collagen expression in chondrocytes. *PLoS ONE* **7**, e40828 10.1371/journal.pone.0040828 (2012). PMC3397959 22815835

[88] Liu, Y., Li, H., Tanaka, K., Tsumaki, N. & Yamada, Y. Identification of an enhancer sequence within the first intron required for cartilage-specific transcription of the α_2_(XI) collagen gene. *J. Biol. Chem.* **275**, 12712–12718 10.1074/jbc.275.17.12712 (2000). 10777565

[89] Cheung, K. et al. Histone ChIP-seq identifies differential enhancer usage during chondrogenesis as critical for defining cell-type specificity. *FASEB J.* **34**, 5317–5331 10.1096/fj.201902061RR (2020). PMC7187454 32058623

[90] Langmead, B. & Salzberg, S. L. Fast gapped-read alignment with Bowtie 2. *Nat. Methods* **9**, 357–359 10.1038/nmeth.1923 (2012). PMC3322381 22388286

[91] Gordon, M. G. et al. lentiMPRA and MPRAflow for high-throughput functional characterization of gene regulatory elements. *Nat. Protoc.* **15**, 2387–2412 10.1038/s41596-020-0333-5 (2020). PMC7550205 32641802

[92] Benjamini, Y. & Hochberg, Y. Controlling the false discovery rate: a practical and powerful approach to multiple testing. *J. R. Stat. Soc. B* **57**, 289–300 10.1111/j.2517-6161.1995.tb02031.x (1995).

[93] Corces, M. R. et al. An improved ATAC-seq protocol reduces background and enables interrogation of frozen tissues. *Nat. Methods* **14**, 959–962 10.1038/nmeth.4396 (2017). PMC5623106 28846090

[94] Chen, S. fastp 1.0: an ultra-fast all-round tool for FASTQ data quality control and preprocessing. *iMeta* **4**, e70078 10.1002/imt2.70078 (2025). PMC12527978 41112039

[95] Zhang, Y. et al. Model-based analysis of ChIP-Seq (MACS). *Genome Biol.* **9**, R137 10.1186/gb-2008-9-9-r137 (2008). PMC2592715 18798982

[96] Fisch, K. M. et al. Identification of transcription factors responsible for dysregulated networks in human osteoarthritis cartilage by global gene expression analysis. *Osteoarthritis Cartilage* **26**, 1531–1538 10.1016/j.joca.2018.07.012 (2018). PMC6245598 30081074

[97] Rauluseviciute, I. et al. JASPAR 2024: 20th anniversary of the open-access database of transcription factor binding profiles. *Nucleic Acids Res.* **52**, D174–D182 10.1093/nar/gkad1059 (2024). PMC10767809 37962376

[98] Hume, M. A., Barrera, L. A., Gisselbrecht, S. S. & Bulyk, M. L. UniPROBE, update 2015: new tools and content for the online database of protein-binding microarray data on protein–DNA interactions. *Nucleic Acids Res.* **43**, D117–D122 10.1093/nar/gku1045 (2015). PMC4383892 25378322

[99] Weirauch, M. T. et al. Determination and inference of eukaryotic transcription factor sequence specificity. *Cell* **158**, 1431–1443 10.1016/j.cell.2014.08.009 (2014). PMC4163041 25215497

[100] Mangan, R. J. et al. Adaptive sequence divergence forged new neurodevelopmental enhancers in humans. *Cell* **185**, 4587–4603 10.1016/j.cell.2022.10.016 (2022). PMC10013929 36423581

[101] Keough, K. C. et al. Three-dimensional genome rewiring in loci with human accelerated regions. *Science* **380**, eabm1696 10.1126/science.abm1696 (2023). PMC10999243 37104607

[102] Pollard, K. S. et al. An RNA gene expressed during cortical development evolved rapidly in humans. *Nature* **443**, 167–172 10.1038/nature05113 (2006). 16915236

[103] Knyazeva, A. S. & Shnaider, T. A. Human accelerated regions: functional studies and methodological approaches (a review). *Cell Tissue Biol.* **20**, 75–95 10.1134/S1990519X25600784 (2026).

[104] Hubisz, M. J. & Pollard, K. S. Exploring the genesis and functions of human accelerated regions sheds light on their role in human evolution. *Curr. Opin. Genet. Dev.* **29**, 15–21 10.1016/j.gde.2014.07.005 (2014). 25156517

[105] Dutrow, E. V. et al. Modeling uniquely human gene regulatory function via targeted humanization of the mouse genome. *Nat. Commun.* **13**, 304 10.1038/s41467-021-27899-w (2022). PMC8758698 35027568

[106] Sayers, E. W. et al. Database resources of the national center for biotechnology information. *Nucleic Acids Res.* **50**, D20–D26 10.1093/nar/gkab1112 (2022). PMC8728269 34850941

[107] Chen, Y. et al. Dynamic chromatin accessibility landscapes of osteoblast differentiation and mineralization. *Biochim. Biophys. Acta Mol. Basis Dis.* **1870**, 166938 10.1016/j.bbadis.2023.166938 (2024). 37931716

[108] Fulco, C. P. et al. Activity-by-contact model of enhancer–promoter regulation from thousands of CRISPR perturbations. *Nat. Genet.* **51**, 1664–1669 10.1038/s41588-019-0538-0 (2019). PMC6886585 31784727

[109] Gasperini, M. et al. A genome-wide framework for mapping gene regulation via cellular genetic screens. *Cell* **176**, 377–390 10.1016/j.cell.2018.11.029 (2019). PMC6690346 30612741

[110] The GTEx Consortium. The GTEx Consortium atlas of genetic regulatory effects across human tissues. *Science* **369**, 1318–1330 10.1126/science.aaz1776 (2020). PMC7737656 32913098

[111] Jung, I. et al. A compendium of promoter-centered long-range chromatin interactions in the human genome. *Nat. Genet.* **51**, 1442–1449 10.1038/s41588-019-0494-8 (2019). PMC6778519 31501517

[112] Bittner, N. et al. Primary osteoarthritis chondrocyte map of chromatin conformation reveals novel candidate effector genes. *Ann. Rheum. Dis.* **83**, 1048–1059 10.1136/ard-2023-224945 (2024). PMC11287644 38479789

[113] Thulson, E. et al. 3D chromatin structure in chondrocytes identifies putative osteoarthritis risk genes. *Genetics* **222**, iyac141 10.1093/genetics/iyac141 (2022). PMC9713432 36099032

[114] Kim, T. K. et al. Widespread transcription at neuronal activity-regulated enhancers. *Nature* **465**, 182–187 10.1038/nature09033 (2010). PMC3020079 20393465

[115] Andersson, R. et al. An atlas of active enhancers across human cell types and tissues. *Nature* **507**, 455–461 10.1038/nature12787 (2014). PMC5215096 24670763

[116] Sloan, C. A. et al. ENCODE data at the ENCODE portal. *Nucleic Acids Res.* **44**, D726–D732 10.1093/nar/gkv1160 (2016). PMC4702836 26527727

[117] Gokhman, D. et al. Gene ORGANizer: linking genes to the organs they affect. *Nucleic Acids Res.* **45**, W138–W145 10.1093/nar/gkx302 (2017). PMC5570240 28444223

[118] Funderburgh, J. L. Keratan sulfate biosynthesis. *IUBMB Life* **54**, 187–194 10.1080/15216540214932 (2002). PMC2874674 12512857

[119] King, S. B. & Singh, M. Primate protein–ligand interfaces exhibit significant conservation and unveil human-specific evolutionary drivers. *PLoS Comput. Biol.* **19**, e1010966 10.1371/journal.pcbi.1010966 (2023). PMC10035887 36952575

[120] Buniello, A. et al. The NHGRI-EBI GWAS Catalog of published genome-wide association studies, targeted arrays and summary statistics 2019. *Nucleic Acids Res.* **47**, D1005–D1012 10.1093/nar/gky1120 (2019). PMC6323933 30445434

[121] Gallego Romero, I. et al. A panel of induced pluripotent stem cells from chimpanzees: a resource for comparative functional genomics. *eLife* **4**, e07103 10.7554/eLife.07103 (2015). PMC4502404 26102527

[122] Viukov, S. et al. Human primed and naïve PSCs are both able to differentiate into trophoblast stem cells. *Stem Cell Rep.* **17**, 2484–2500 10.1016/j.stemcr.2022.09.008 (2022). PMC9669397 36270280

[123] Yamada, D. et al. Induction and expansion of human PRRX1^+^ limb-bud-like mesenchymal cells from pluripotent stem cells. *Nat. Biomed. Eng.* **5**, 926–940 10.1038/s41551-021-00778-x (2021). PMC12680540 34373601

[124] Takao, T., Yamada, D. & Takarada, T. A protocol to induce expandable limb-bud mesenchymal cells from human pluripotent stem cells. *STAR Protoc.* **3**, 101786 10.1016/j.xpro.2022.101786 (2022). PMC9617203 36317170

[125] Akiyama, H. et al. Osteo-chondroprogenitor cells are derived from Sox9 expressing precursors. *Proc. Natl Acad. Sci.* **102**, 14665–14670 10.1073/pnas.0504750102 (2005). PMC1239942 16203988

[126] van de Geijn, B., McVicker, G., Gilad, Y. & Pritchard, J. K. WASP: allele-specific software for robust molecular quantitative trait locus discovery. *Nat. Methods* **12**, 1061–1063 10.1038/nmeth.3582 (2015). PMC4626402 26366987

[127] Love, M. I., Huber, W. & Anders, S. Moderated estimation of fold change and dispersion for RNA-seq data with DESeq2. *Genome Biol.* **15**, 550 10.1186/s13059-014-0550-8 (2014). PMC4302049 25516281

[128] Zhu, A., Ibrahim, J. G. & Love, M. I. Heavy-tailed prior distributions for sequence count data: removing the noise and preserving large differences. *Bioinformatics* **35**, 2084–2092 10.1093/bioinformatics/bty895 (2019). PMC6581436 30395178

[129] Logsdon, G. A. et al. Complex genetic variation in nearly complete human genomes. *Nature* **644**, 430–441 10.1038/s41586-025-09140-6 (2025). PMC12350169 40702183

[130] Liao, W. W. et al. A draft human pangenome reference. *Nature* **617**, 312–324 10.1038/s41586-023-05896-x (2023). PMC10172123 37165242

[131] Mao, Y. et al. Structurally divergent and recurrently mutated regions of primate genomes. *Cell* **187**, 1547–1562 10.1016/j.cell.2024.01.052 (2024). PMC10947866 38428424

[132] Yoo, D. et al. Complete sequencing of ape genomes. *Nature* **641**, 401–418 10.1038/s41586-025-08816-3 (2025). PMC12058530 40205052

[133] Porsborg, P. S. et al. Long-read sequencing of primate testis and human sperm allows identification of recombination events in individuals. *Nat. Commun.* **16**, 10337 10.1038/s41467-025-65248-3 (2025). PMC12644622 41285744

[134] Nelson, D. R. et al. A near telomere-to-telomere phased reference assembly for the male mountain gorilla. *Sci. Data* **12**, 842 10.1038/s41597-025-05114-5 (2025). PMC12098815 40404646

[135] Camacho, C. et al. BLAST+: architecture and applications. *BMC Bioinformatics* **10**, 421 10.1186/1471-2105-10-421 (2009). PMC2803857 20003500

[136] Shao, Y. et al. Phylogenomic analyses provide insights into primate evolution. *Science* **380**, 913–924 10.1126/science.abn6919 (2023). 37262173

[137] Poszewiecka, B., Gogolewski, K., Karolak, J. A., Stankiewicz, P. & Gambin, A. PhaseDancer: a novel targeted assembler of segmental duplications unravels the complexity of the human chromosome 2 fusion going from 48 to 46 chromosomes in hominin evolution. *Genome Biol.* **24**, 205 10.1186/s13059-023-03022-8 (2023). PMC10496407 37697406

[138] Scrucca, L., Fop, M., Murphy, T. B. & Raftery, A. E. mclust 5: clustering, classification and density estimation using Gaussian finite mixture models. *R J.* **8**, 289–317 10.32614/RJ-2016-021 (2016). PMC5096736 27818791

[139] Ladner, Y. D., Alini, M. & Armiento, A. R. in *Cartilage Tissue Engineering: Methods in Molecular Biology*, Vol. 2598 (eds Stoddart, M. J. et al.) 115–121 (2023). 10.1007/978-1-0716-2839-3_1 36355280

[140] Neumann, A. J. et al. Human articular cartilage progenitor cells are responsive to mechanical stimulation and adenoviral-mediated overexpression of bone-morphogenetic protein 2. *PLoS ONE* **10**, e0136229 10.1371/journal.pone.0136229 (2015). PMC4546341 26292283

[141] Temple-Wong, M. M. et al. Biomechanical, structural, and biochemical indices of degenerative and osteoarthritic deterioration of adult human articular cartilage of the femoral condyle. *Osteoarthritis Cartilage* **17**, 1469–1476 10.1016/j.joca.2009.04.017 (2009). PMC2763930 19464244

[142] Tsuchida, A. I. et al. Cytokine profiles in the joint depend on pathology, but are different between synovial fluid, cartilage tissue and cultured chondrocytes. *Arthritis Res. Ther.* **16**, 441 10.1186/s13075-014-0441-0 (2014). PMC4201683 25256035

[143] Vonk, L. A. et al. Mesenchymal stromal/stem cell-derived extracellular vesicles promote human cartilage regeneration in vitro. *Theranostics* **8**, 906–920 10.7150/thno.20746 (2018). PMC5817101 29463990

[144] de Magalhães, J. P. et al. Human ageing genomic resources: updates on key databases in ageing research. *Nucleic Acids Res.* **52**, D900–D908 10.1093/nar/gkad927 (2024). PMC10767973 37933854

[145] Charvet, C. J., Ofori, K., Falcone, C. & Rigby Dames, B. A. Transcription, structure, and organoids translate time across the lifespan of humans and great apes. *PNAS Nexus* **2**, pgad230 10.1093/pnasnexus/pgad230 (2023). PMC10406161 37554928

