# Supplementary material for: The gene-regulatory evolution of the human skeleton
Source: Nature. 2026 Sep 23;658(8136):698–706. doi: 10.1038/s41586-026-11053-x (PMC13668659; doi:10.1038/s41586-026-11053-x)
Supplement: Supplementary file 1 [file 41586_2026_11053_MOESM2_ESM.pdf]

nature portfolio

Corresponding author(s): David Gokhman, Fumitaka Inoue

Last updated by author(s): 20.07.2026

Reporting Summary

Nature Portfolio wishes to improve the reproducibility of the work that we publish. This form provides structure for consistency and transparency in reporting. For further information on Nature Portfolio policies, see our Editorial Policies and the Editorial Policy Checklist.

Please do not complete any field with "not applicable" or n/a. Refer to the help text for what text to use if an item is not relevant to your study.
For final submission: please carefully check your responses for accuracy; you will not be able to make changes later.

Statistics

For all statistical analyses, confirm that the following items are present in the figure legend, table legend, main text, or Methods section.

n/a	Confirmed	
☐	☒	The exact sample size (*n*) for each experimental group/condition, given as a discrete number and unit of measurement
☐	☒	A statement on whether measurements were taken from distinct samples or whether the same sample was measured repeatedly
☐	☒	The statistical test(s) used AND whether they are one- or two-sided<br>*Only common tests should be described solely by name; describe more complex techniques in the Methods section.*
☐	☒	A description of all covariates tested
☐	☒	A description of any assumptions or corrections, such as tests of normality and adjustment for multiple comparisons
☐	☒	A full description of the statistical parameters including central tendency (e.g. means) or other basic estimates (e.g. regression coefficient) AND variation (e.g. standard deviation) or associated estimates of uncertainty (e.g. confidence intervals)
☐	☒	For null hypothesis testing, the test statistic (e.g. *F*, *t*, *r*) with confidence intervals, effect sizes, degrees of freedom and *P* value noted<br>*Give P values as exact values whenever suitable.*
☐	☒	For Bayesian analysis, information on the choice of priors and Markov chain Monte Carlo settings
☐	☒	For hierarchical and complex designs, identification of the appropriate level for tests and full reporting of outcomes
☐	☒	Estimates of effect sizes (e.g. Cohen's *d*, Pearson's *r*), indicating how they were calculated

Our web collection on statistics for biologists contains articles on many of the points above.

Software and code

Policy information about availability of computer code

Data collection	No software was used for data collection
Data analysis	bowtie2 v.2.3.5.1, samtools v.1.9, MPRAnalyze 1.9.1, STAR (v.2.7.11b), Picard v.2.18.27, DESeq2 v.1.34.0, BLAST+ (v2.14.0), mclust R package (v6.0.0), FastQC v0.12.1, fastp v1.0.1, Picard MarkDuplicates v3.0.0, MACS3 v3.0.1. Codes: https://github.com/GokhmanLabOrganization/the_gene_regulatory_evolution_of_the_human_skeleton and https://github.com/GokhmanLabOrganization/differential-TF-binding

For manuscripts utilizing custom algorithms or software that are central to the research but not yet described in published literature, software must be made available to editors and reviewers. We strongly encourage code deposition in a community repository (e.g. GitHub). See the Nature Portfolio guidelines for submitting code & software for further information.

Data

Policy information about availability of data

All manuscripts must include a data availability statement. This statement should provide the following information, where applicable:

- Accession codes, unique identifiers, or web links for publicly available datasets
- A description of any restrictions on data availability
- For clinical datasets or third party data, please ensure that the statement adheres to our policy

MPRA sequencing data was deposited at GSE316891. Differentiated hybrids sequencing data was deposited at GSE316892. ATAC-seq and H3K27ac data was desposited in DDBJ under accession number PRJDB40123

Research involving human participants, their data, or biological material

Policy information about studies with human participants or human data. See also policy information about sex, gender (identity/presentation), and sexual orientation and race, ethnicity and racism.

Reporting on sex and gender	Sex is mentioned for all human samples.
Reporting on race, ethnicity, or other socially relevant groupings	Age is specified for each individual or a group of individuals.
Population characteristics	No data on population characteristics was collected or used
Recruitment	Formalin-fixed human specimens were obtained from the Farkas Family Center for Anatomical Research and Education (CARE), Rappaport Faculty of Medicine, Technion – Israel Institute of Technology. All tissues were collected post-mortem.
Ethics oversight	Approval for the derivation of human iPSC lines used in this study was granted by the University of Chicago Institutional Review Board, protocol 11–0524. Human donors of the cells used in this study consented to the use of their cells (fibroblasts) to generate iPSCs for studies of evolution and cross-species comparisons, and to the generation of other cell types that would be derived from these iPSCs. Donors consented to the deposition of any resulting data. Generation of hybrid human-ape iPSCs was approved by the Weizmann Institutional Review Board (IRB) (protocol 1586-2).

Please select the one below that is the best fit for your research. If you are not sure, read the appropriate sections before making your selection.

☒ Life sciences ☐ Behavioural & social sciences ☐ Ecological, evolutionary & environmental sciences

For a reference copy of the document with all sections, see nature.com/documents/nr-reporting-summary-flat.pdf

Life sciences study design

All studies must disclose on these points even when the disclosure is negative.

Sample size	DMMB asasy - 42 human individuals,7 ape individuals. Hybrid cells - 2 human-gorilla hybrids,3 human-chimp hybrids. We took the maximum samples available. Similar sample sizes in other studies, as well as in the current study, are able to reach statistical significance
Data exclusions	No Data was excluded
Replication	DMMB - 3 plate reading replicates. Reproducibility in all experiments was determined using correlation between replicates (reported in the manuscript). All replications were successful.
Randomization	Randomization is used to prevent placebo and confounding effects when individuals are assigned to treatment groups. These do not apply to our study design (no treatment groups)
Blinding	Blinding is used to prevent placebo and confounding effects when individuals are assigned to treatment groups. these do not apply to our study design as there was no treatment groups

Behavioural & social sciences study design

All studies must disclose on these points even when the disclosure is negative.

Study description	
Research sample	
Sampling strategy	
Data collection	
Timing	
Data exclusions	
Non-participation	
Randomization	

Ecological, evolutionary & environmental sciences study design

All studies must disclose on these points even when the disclosure is negative.

Study description	
Research sample	
Sampling strategy	
Data collection	
Timing and spatial scale	
Data exclusions	
Reproducibility	
Randomization	
Blinding	

Did the study involve field work? ☐ Yes ☐ No

Field work, collection and transport

Field conditions	
Location	
Access & import/export	
Disturbance	

Reporting for specific materials, systems and methods

We require information from authors about some types of materials, experimental systems and methods used in many studies. Here, indicate whether each material, system or method listed is relevant to your study. If you are not sure if a list item applies to your research, read the appropriate section before selecting a response.

Materials & experimental systems

n/a	Involved in the study
☐	☒ Antibodies
☐	☒ Eukaryotic cell lines
☒	☐ Palaeontology and archaeology
☒	☐ Animals and other organisms
☒	☐ Clinical data
☒	☐ Dual use research of concern
☒	☐ Plants

Methods

n/a	Involved in the study
☒	☐ ChIP-seq
☐	☒ Flow cytometry
☒	☐ MRI-based neuroimaging

Antibodies

Antibodies used	anti-H3K27ac antibody (ab4729, Abcam), anti-rabbit IgG (ab6702, Abcam), TRA-1-60 antibody, NANOG antibody , OCT4 antibody (StemLight™ Pluripotency IF Antibody Sampler Kit (#9656) for all three), SSEA4 antibody, OTX2 antibody, Sox17 antibody, Brachyury antibody (Human Pluripotent Stem Cell Functional Identification Kit (R&D Systems, Cat. #SC027B for all three).
Validation	All antibodies were validated by vendors:Anti-Histone H3 (acetyl K27) antibody - ChIP Grade (ab4729) was first used in a scientific publication in 1975 and has been cited over 2188 times in peer reviewed journals. The Human Pluripotent Stem Cell Functional Identification Kit has been used in 127 studies according to the manufacturer's website. StemLight™ Pluripotency IF Antibody Sampler Kit (#9656) has been used in 59 studies according to the manufacturer's website.

Eukaryotic cell lines

Policy information about cell lines and Sex and Gender in Research

Cell line source(s)	HEK293T - ATCC, CRL-3216, Chondrocytes - Cell Applications, 402-05f
Authentication	HEK293T cells were authenticated by venter (ATCC). Chondrocytes were validated by vendor (Cell Applications): Each lot is tested for its ability to attach and spread on tissue culture ware surface, and proliferate in Chondrocyte Growth Medium. HIV1, Hepatitis B, Hepatitis C,bacteria, yeast, fungi not detected)
Mycoplasma contamination	Not detected
Commonly misidentified lines (See ICLAC register)	No commonly misidentified cell lines were used.

Palaeontology and Archaeology

Specimen provenance	
Specimen deposition	
Dating methods	

☐ Tick this box to confirm that the raw and calibrated dates are available in the paper or in Supplementary Information.

Ethics oversight	

Note that full information on the approval of the study protocol must also be provided in the manuscript.

Animals and other research organisms

Policy information about studies involving animals; ARRIVE guidelines recommended for reporting animal research, and Sex and Gender in Research

Laboratory animals	
Wild animals	
Reporting on sex	
Field-collected samples	
Ethics oversight	

Note that full information on the approval of the study protocol must also be provided in the manuscript.

Clinical data

Policy information about clinical studies

All manuscripts should comply with the ICMJE guidelines for publication of clinical research and a completed CONSORT checklist must be included with all submissions.

Clinical trial registration	
Study protocol	
Data collection	
Outcomes	

Dual use research of concern

Policy information about dual use research of concern

Hazards

Could the accidental, deliberate or reckless misuse of agents or technologies generated in the work, or the application of information presented in the manuscript, pose a threat to:

No | Yes

- [ ] [ ] Public health
- [ ] [ ] National security
- [ ] [ ] Crops and/or livestock
- [ ] [ ] Ecosystems
- [ ] [ ] Any other significant area

Experiments of concern

Does the work involve any of these experiments of concern:

No | Yes

- [ ] [ ] Demonstrate how to render a vaccine ineffective
- [ ] [ ] Confer resistance to therapeutically useful antibiotics or antiviral agents
- [ ] [ ] Enhance the virulence of a pathogen or render a nonpathogen virulent
- [ ] [ ] Increase transmissibility of a pathogen
- [ ] [ ] Alter the host range of a pathogen
- [ ] [ ] Enable evasion of diagnostic/detection modalities
- [ ] [ ] Enable the weaponization of a biological agent or toxin
- [ ] [ ] Any other potentially harmful combination of experiments and agents

Plants

Seed stocks

Novel plant genotypes

Authentication

ChIP-seq

Data deposition

- [ ] Confirm that both raw and final processed data have been deposited in a public database such as GEO.
- [ ] Confirm that you have deposited or provided access to graph files (e.g. BED files) for the called peaks.

Data access links
May remain private before publication.

Files in database submission

Genome browser session
(e.g. UCSC)

Methodology

Replicates

Sequencing depth

Antibodies

Peak calling parameters

Data quality

Software

Flow Cytometry

Plots

Confirm that:

[X] The axis labels state the marker and fluorochrome used (e.g. CD4-FITC).

[X] The axis scales are clearly visible. Include numbers along axes only for bottom left plot of group (a 'group' is an analysis of identical markers).

[X] All plots are contour plots with outliers or pseudocolor plots.

[X] A numerical value for number of cells or percentage (with statistics) is provided.

Methodology

Sample preparation: iPSCs were co-cultured and treated with PEG to induce fusion. Cells were subsequently sorted according to fluorescent dyes. Double-positive cells containing both dyes were isolated as fused cells.

Instrument: BD FACSAria II

Software: BD FACSDiva 8.0.1

Cell population abundance: The double positive population of cells represented 10%

Gating strategy: gating out doublets, gating out dead cells, sorting for red and green double positives; FSC forward scatter, SSC side scatter, -A area, -H height; Pacific Blue measures DAPI, FITC measures Green CMFDA, and APC measures Deep Red

[X] Tick this box to confirm that a figure exemplifying the gating strategy is provided in the Supplementary Information.

Magnetic resonance imaging

Experimental design

Design type

Design specifications

Behavioral performance measures

Imaging type(s)

Field strength

Sequence & imaging parameters

Area of acquisition

Diffusion MRI [ ] Used [ ] Not used

Preprocessing

Preprocessing software

Normalization

Normalization template

Noise and artifact removal

Volume censoring

Statistical modeling & inference

Model type and settings

Effect(s) tested

Specify type of analysis: ☐ Whole brain ☐ ROI-based ☐ Both

Statistic type for inference
(See Eklund et al. 2016)

Correction

Models & analysis

n/a	Involved in the study
☐	☐ Functional and/or effective connectivity
☐	☐ Graph analysis
☐	☐ Multivariate modeling or predictive analysis

Functional and/or effective connectivity

Graph analysis

Multivariate modeling and predictive analysis

This checklist template is licensed under a Creative Commons Attribution 4.0 International License, which permits use, sharing, adaptation, distribution and reproduction in any medium or format, as long as you give appropriate credit to the original author(s) and the source, provide a link to the Creative Commons license, and indicate if changes were made. The images or other third party material in this article are included in the article's Creative Commons license, unless indicated otherwise in a credit line to the material. If material is not included in the article's Creative Commons license and your intended use is not permitted by statutory regulation or exceeds the permitted use, you will need to obtain permission directly from the copyright holder. To view a copy of this license, visit http://creativecommons.org/licenses/by/4.0/
