# Supplementary material for: The gene-regulatory evolution of the human skeleton
Source: Nature. 2026 Sep 23;658(8136):698–706. doi: 10.1038/s41586-026-11053-x (PMC13668659; doi:10.1038/s41586-026-11053-x)
Supplement: Supplementary file 2 [file 41586_2026_11053_MOESM4_ESM.pdf]

nature portfolio

Peer Review File

The Gene Regulatory Evolution of the Human Skeleton

Corresponding Author: Dr David Gokhman

Any redactions in this file are there to maintain patient confidentiality, the confidentiality of unpublished data, or to remove third-party material.

This file contains all reviewer reports in order by version, followed by all author rebuttals in order by version.

Version 0:

Reviewer comments:

Referee #1

(Remarks to the Author)
The study by David Gokhman's laboratory explores the human-specific evolution of genetic regulatory elements in skeletal tissues. The authors utilized highly advanced genomic approaches, including massive parallel reporter assays (MPRAs) and interspecies hybrid iPSC systems. Major findings include predicted evolutionary changes in cartilage glycosaminoglycan (GAG) content and regulation of the Hedgehog signaling pathway, particularly involving the EVC gene. The manuscript provides deep and conceptually novel insights into human evolution and contains a wealth of interesting and valuable data. The experimental tools developed in this work are highly multidisciplinary and broadly applicable.

The manuscript is well written, the data are clearly presented, and most conclusions are appropriately balanced. Overall, this is a nice and ambitious study addressing an important question in human evolution and providing deep insights into the evolution of cis-regulatory elements underlying skeletal divergence between humans and apes.

I do not have sufficient expertise to critically evaluate all technical aspects of evolutionary genetics or MPRA methodology. Therefore, my comments primarily focus on skeletal aspects of the work (my primary expertise area). Below, I outline my concerns and suggestions for further strengthening the biological interpretation and the manuscript's overall impact.

Major Points (arranged by importance, with the most important first)

1. The cCRE-based predictions suggest an evolutionarily driven decrease in GAG content in human cartilage relative to apes. However, this proposed reduction may be functionally compensated by additional anchoring sites that evolved within the human aggrecan gene. It is therefore unclear whether this represents an adaptive evolutionary shift, divergence, or fluctuations of unclear nature.

To assess GAG content functionally, the authors compared human samples with those from a single chimpanzee. Given the biological variability of cartilage, this sample size is insufficient to draw robust conclusions.

The authors further argue that mechanical loading reduces proteoglycan and GAG content (refs 79–83) and therefore focus on the thumb, which is not involved in knuckle-walking (lines 542–543). However, mechanical loading has a dual role in cartilage biology. It is widely accepted that physiological loading promotes cartilage health and increases proteoglycan synthesis (PMIDs: 2760736; 8666595; 1787831). In light of this, the similarity in GAG concentrations observed between humans and chimpanzees in the 2nd–4th metacarpophalangeal and interphalangeal joints (Fig. 4g) weakens the interpretation of evolutionary deterioration of human cartilage.

I therefore strongly recommend a more comprehensive comparison of GAG levels across multiple chimpanzees, gorillas, and humans. While I recognize the difficulty of obtaining such samples, specimens from animals that die of natural causes in zoological institutions may be accessible.

In addition, differentiation of chimpanzee, gorilla, and human iPSCs into cartilage and direct comparison of their matrix properties would provide additional and strong functional validation of the evolutionary predictions.

2. The section dedicated to Hedgehog signaling appears disconnected from the main narrative and reads almost as an independent sub-project. Its integration into the overall story should be improved.
One possible solution would be to introduce the Hedgehog pathway earlier, framing the study as a continuation of prior work (ref 42), and positioning cartilage evolution as a downstream consequence of evolutionary modulation of this pathway. Given that Hedgehog signaling is a central regulator of cartilage development and skeletal morphology, structuring the manuscript around this axis could improve narrative coherence.

3. Although the manuscript is generally well written, several sections appeared mixed between Results and Discussion. Extended interpretative passages (e.g., lines 608–631) would be more appropriate in the Discussion and should be shortened or relocated.
Conversely, parts of the Discussion could be more focused on cartilage biology and Hedgehog signaling. For example, the section discussing the role of GAGs in the immune system (lines 879–896) is not directly relevant to the obtained results and could be replaced or condensed in favor of a discussion of GAG-related skeletal disorders, which are currently addressed in the Results section (lines 608–631). This reorganization would improve thematic consistency.

Minor Comments
1. The abstract states that humans have a greater susceptibility to degenerative joint disease than chimpanzees. Please provide a supporting reference.

2. Line 9: Replace “large brain” with “large skull,” as the brain itself is not a skeletal feature.

3. Line 86: Replace “the solo resident cell type” with “the major resident cell type” or “the primary functional resident cell type.” Superficial zone cells include chondro-progenitors and are not exclusively mature chondrocytes.

4. The integration of MPRA and hybrid cell approaches to identify human-specific cis-regulated genes (Fig. 2e; lines 331–338) is conceptually important but is not presented clearly enough. If I interpret the analysis correctly, the authors compare the fractions of up- and downregulated human alleles associated with upregulation of cCRE activity. In this case, the substantial overlap between up- and downregulated groups in the violin plots suggests modest predictive power. Please clarify in the manuscript: (i) the total number of up- and downregulated human-specific cis-regulated genes (out of 4463 identified), (ii) the proportion of each group overlapping with active cCREs, (iii) the interpretation and limitations of this overlap.

5. Lines 353–356: Please provide supplementary figures showing enrichment results for each database analyzed. In Figure 3b, enrichment for GAG metabolism is shown, but the legend mentions both GO and HPO datasets. Please clarify whether this is derived from one database or a combined analysis.

6. To complement and strengthen the cCRE-based pathway enrichment analysis (Fig. 3), please perform a similar enrichment analysis for the 4463 human-specific cis-regulated genes identified in hybrid cells and present the enriched pathways.

7. In Figure 4, the authors link human-lineage regulatory divergence to EVC and Hedgehog signaling. However, functional validation of pathway activity is lacking. It would significantly strengthen the manuscript to directly demonstrated changed activity of the Hedgehog pathway. For example, utilizing Prrx1+ iPSC-derived osteoprogenitors and/or mature chondrocytes from human, chimpanzee, and gorilla iPSCs. The differentiation protocol employed by the authors was recently further extended (https://doi.org/10.1101/2025.01.09.631479), which may be particularly helpful for this issue and major issue 1.

Referee #2

(Remarks to the Author)
I co-reviewed this manuscript with one of the reviewers who provided the listed reports.

Referee #3

(Remarks to the Author)
This manuscript includes comprehensive experimental evidence that human specific genetic variation has contributed to skeletal evolution in our species. In particular, the authors focus on several data types and analyses:

• First, the authors use MPRAs in human chondrocytes to assay 561,410 SNPs that are fixed in humans in putative regulatory elements. They find that 65,576 of tested fragments have regulatory activity in the query cell type, and that 15,077 loci have evidence for human-specific activity when comparing the derived and ancestral allele. These data point to down regulation of polysaccharide (glycosaminoglycan) biosynthesis genes.
• Second, they generate and assay human-ape hybrid cells (including human-chimp AND human-gorilla) — to identify between species gene expression differences in a common trans cellular background — and differentiate them into osteochondral progenitors. Specifically, they identify genes in both hybrid cells with between species expression differences indicating regulatory evolution (~4100 chimp specific and ~4400 human specific). These data confirm down regulation of polysaccharide (glycosaminoglycan) biosynthesis genes.
• Third, the authors followed up on the reduction in polysaccharide content in human compared with chimpanzee cartilage

using primary samples, finding that in the thumb – where both species experience similar mechanical stress – of a single chimpanzee exhibited 2.3x higher GAG content than 21 humans. They also use loss-of-function phenotypes from human monogenic disorders to predict that downregulation of GAG would result in skeletal changes consistent with those observed between species.
• Finally, the identify genome-wide outliers of differential activity at the EVC locus, and provide several analyses to identify the likely causal SNP as well as the evolutionary history of the locus.
Overall, I found the work to be exciting, comprehensive, and a pleasure to read; the authors provide many different lines of evidence for each conclusion, and the paper addresses an important and understudied topic in human evolution. However, I do have a few methodological questions that I think are important to dig into to evaluate some of the conclusions. Also, maybe I missed it, but I didn't see a code availability statement for the complete set of analyses performed in this manuscript, and I sometimes found it hard to track down the specific statistical test accompanying a p-value provided in the main text. It would be great to include a GitHub with the processed data matrices and the code used to perform statistical analyses (especially main analyses like differential expression, differential activity, figures, etc). Given that there is a lot going on in this manuscript (because the authors have so many experiments and analyses), seeing the code will be important for making sure all statistical choices about filtering, modeling, multiple hypothesis testing correction, etc are clear and transparent. I appreciate the comprehensive MPRA QC manual, test data sets, and associated scripts, but it is not the same as what I am asking for.

Major comments:

In Fig 1D, it looks like the cCREs only have minimally higher activity than the negative controls. Could you separate the cCREs that you call as 1) having no regulatory activity, 2) having regulatory activity but no allelic differences, and 3) having regulatory activity and allelic differences? Maybe this could be an inset or something to show that the ~18% you call as active hopefully look more like the positive controls. Otherwise, please provide some explanation about these patterns.

The authors asked whether "genes whose human allele is upregulated in the hybrids are enriched for upregulating cCREs in the MPRA, and vice versa." They find that this is weakly true, but they restrict to "genes linked to ≥ 5 differentially active cCREs", which seems like a lot. What happens if this threshold is reduced? And how many genes met this threshold? Some of the weak overlap may also be attributed to chondrocytes and osteochondral progenitors not being the same cell type, so I wonder if you could subset to genes you know are similarly expressed in both cell types. In general, could you also give some intuition or analysis on how similar chondrocytes and osteochondral progenitors are, since inference is linked across the two cell types.

The observation that a single chimpanzee exhibited 2.3x higher GAG content than 21 humans is interesting but obviously very limited in having access to only 1 ape biospecimen. How are the authors producing a p-value from 1 observation (I cannot find the analysis strategy in the methods, and only a p-value is provided in the main text)? I think this analysis needs to be reframed as much more exploratory. For example the authors state "our results indicate… reduced GAG content in human relative to chimpanzee joints" but this is really the joint of a single chimpanzee. I also think it's also important to provide some information about the standard error across the 3 technical replicates, since a lot of inference is resting on the single chimpanzee data point. Additionally, were the human donors age and sex matched to the chimpanzee?

The reader needs much more information about the performance of the MPRALegNet model to evaluate its use. What procedures were done to understand model performance? How accurate are the predictions from this model? And then the point of this analysis is just to disentangle whether chr4:5775029 versus chr4:5775100 is the likely SNPs driving between species differences (because these 2 SNPs were assayed on the same fragment)? I was confused why it was necessary to train a predictive model when the region was already evaluated empirically, but I assume this is the goal.

My biggest concern, which the authors acknowledge, is that the MPRA is only conducted in human cells, and "the trans environment of chondrocytes might have changed since the human-chimpanzee ancestor". I would like to see the authors convince the reader a bit more that the trans environment is likely similar enough between human and chimp that we should not be concerned about this technical limitation, but only citations to previous work are provided. Are there analyses the authors can include to assuage this concern? For example - what's the correlation in expression levels between the human chondrocytes and chimp osteochondral progenitors? It would also be useful to know what this looks like for human chondrocytes versus human osteochondral progenitors. Obviously, the best comparison would be RNA-seq from human chondrocytes vs chimp chondrocytes; I'm guessing the authors do not have those data, but any between species comparisons for a relevant cell type — with special attention to the genes and pathways they are trying to highlight — would be helpful.

The authors provided some analyses to attempt to link the between species datasets to within human variation and human diseases (e.g., osteoarthritis), but I felt like this could have gone further. If the argument is that genes with cCREs with significant between species differences are important for skeletal traits, I would also expect this to be true within species when variation occurs in these regions. For example, are genes with cCREs with significant between species differences enriched for GWAS associations with human skeletal diseases or traits? Or is genetic variation within humans that occurs in these cCREs more likely to generate eQTL? Analyses along these lines would be helpful for understanding the likely relevance of these regions for human disease.

Minor comments:

To identify human specific fixed alleles, the authors compared ape genomes to gnomAD. Are these variants also fixed in 1000 Genomes and HGDP, which include a more global and diverse set of whole genome sequences? For example, it would be helpful to check the variants here: https://pmc.ncbi.nlm.nih.gov/articles/PMC11216312/

To narrow down the list of human specific variants to include in the MPRA, the authors overlapped the candidate SNP list with putative regulatory elements from SCREEN v2, which draws on active chromatin marks across 839 human cell types and tissues. The vast majority of these 839 human cell types are not relevant to the skeleton. Why did the authors choose to use this broad approach, rather than subsetting to putative regulatory elements in cell types of interest?

The authors state that there is “higher activity of cCREs that (i) overlap open chromatin in chondrocytes ($P = 4.9 \times 10^{-324}$, Kolmogorov-Smirnov test), or (ii) overlap promoter marks ($P = 1.3 \times 10^{-307}$, Kolmogorov-Smirnov test).” Is this enrichment higher than if they use annotations from other cell types? In other words, how specific is the enrichment/signal to chondrocytes?

Could you add an x=y line to Fig 1c?

The authors state that they found ~4100 chimp specific and ~4400 human specific cis regulatory expression changes. What about gorilla specific expression differences?

I was surprised to not see any general tests for TF binding enrichment analyses for the differentially expressed cCREs. Are these sequences enriched for any known TF motifs?

The total pool of candidate sequences were divided across 9 sub libraries to accommodate technical constraints. Were any sequences repeated across sub libraries to understand batch effects? Were there any batch effects on activity levels?

The authors state that “Some cCREs with multiple barcodes had one or more barcodes with extreme RNA read counts compared to the other barcodes of the same cCRE allele, while not having higher DNA counts. Such outliers are likely attributed to hyperactive integration loci, and do not represent the true endogenous activity of the cCRE.” Can you provide some information on how often this lack of replication happens?

The authors use both MAD score and RNA/DNA ratio as a measure of activity. Could you provide some information about how correlated these are? They seem to be used interchangeably.

In methods section 6.3.3, could you provide some more information on what’s going on with seq325030?

In methods section 6.9.1, did a gene need to be differentially expressed in both chimp-human and gorilla-human comparisons? Or just significantly differentially expressed in at least one comparison, with the other comparison used to polarize?

In extended data figure 2, why are there a large number of cCREs that have RNA/DNA >> 0 but are not called active? The correlation between replicates generally looks good, so I am surprised if there is a large class of elements with high enough standard errors that they are not reaching significance thresholds.

Referee #4

(Remarks to the Author)
Yan et al. present an in-depth analysis of cis-regulatory divergence on the human lineage with respect to chondrocytes.

Overview:
The authors use a large-scale MPRA in human chondrocytes to test over 300,000 regulatory regions containing more than 500,000 DNA substitutions that are fixed in humans. This provides an overview of cis-regulatory divergence in human chondrocytes at the DNA level, due to substitution mutations. Additionally, the authors use human-chimpanzee and human-gorilla cell fusions to create allotetraploid cells that they differentiated into chondrocytes. They then compare the expression levels of the human and non-human alleles in these allotetraploid chondrocytes, which allows them to polarize human-chimp gene expression differences into changes on the human or chimp branches. This analysis provides an overview of cis-regulatory divergence in human chondrocytes at the RNA level. In both of these analyses, the authors note the cis-regulatory downregulation of the GAG biosynthesis pathway in humans, compared to the other great apes. Consistent with this observation, the authors also see reduced GAG content in human joints compared to chimpanzees. To understand additional phenotypic changes that could be associated with this cis-regulatory downregulation, the authors use phenotypes from human disorders linked to this pathway to propose that human-specific skeletal changes and osteoarthritis risk are likely impacted by these cis-regulatory changes.

This is an impressive body of work that will be useful to the scientific community both as a resource and for its biological discoveries. While I have some comments on the manuscript (see below), the comments do not dampen my enthusiasm for this work.

Comments:
1) The breadth of the MPRA experiment is impressive, but it was difficult to understand what percent of the human genome could be assessed for fixed human differences, and what percentage of the genome may still hold fixed alleles that have not

been tested yet. It would be helpful to know the percentage of the genome where the great ape allele could be assessed (i.e. passing the read depth filters, more than 10bp away from an indel, etc), the human allele could be confidently called across all human samples (it was a bit unclear if the authors only required a lack of a variant call, or if all samples needed a confident call of matching the human reference assembly for the gnomAD samples), and avoided the problematic tracks from UCSC and ENCODE. I don't disagree with the filters that the authors applied in order to have a high-confidence set, but I was left wondering how much of the genome was not included due to these conservative filters.

2) The main text says there were 1,527 positive controls, 3,436 negative controls, and 1,329 sequence pairs as controls for differential expression. In the methods section "MPRA controls," I only found 38 + 12 positive controls, 100 + 100 + 100 negative controls, and 407 * 2 controls for differential expression. I couldn't figure out how to make the numbers in the main text and the methods match up.

3) The MPRA library was filtered for being in regulatory elements (i.e., SCREEN), but I believe this regulatory signal could have come from tissues/cells unrelated to chondrocytes. The main text does say that the MPRA-positive fragments in the library tend to overlap open chromatin, but I am curious about the exact numbers of MPRA-positive/negative elements that do/don't overlap open chromatin. Would MPRA-positive fragments in closed chromatin be considered false positives (in the context of the MPRA experiment)?

4) The cell fusions are an elegant and powerful system for studying cis-regulatory changes. When the cell fusions between humans and other great apes are first introduced, I felt that the description was too brief; there was a mention of more details about the tradeoffs of this system in the discussion, but I did not find this in the discussion. For example, the text states "... fully tetraploid hybrids of closely related species ... maintain the balanced gene dosage of diploid cells ..." This is largely true, but one of the studies cited to support this claim (PMID: 34921118) shows that about 180 genes change their regulation by more than 2 fold when comparing standard human/chimp diploid cells with autotetraploid cells. Additionally, tetraploid human pregnancies rarely go to term and are associated with skeletal changes (PMID: 14632567), which hints at gene regulatory differences. I think it would benefit the reader to have a more thorough description of the strengths and limitations of the system.

5) The authors use phrases like "human-ape hybrid" to describe the cells, which has both pros and cons. The term hybrid is broadly accessible, which is helpful, but also evokes the idea of reproduction between the species. "Allotetraploid" is a more precise description of the cells, although it is a less well known term. It may be beneficial to incorporate some suggestions from the paper, PMID: 35082442, in the revision.

6) I am wondering if the p-values related to the lineage specific selection test need to be corrected for multiple tests. It was unclear to me from the main text and methods section if the p-values were corrected or not. I am thinking that there should be a correction because the main text reads that a number of gene annotation databases (i.e., IPA, GO, HPA, GAD) were assembled and then the authors searched across the annotations for terms with many affected genes, including which terms had the most down-regulation associated with them. Example, "Similarly, we found that in IPA, the most significantly downregulated process was glycosaminoglycan metabolism." This reads to me that the authors searched across many terms to identify those with the most downregulation, but then only applied the lineage-specific statistical test to the most extreme term that they identified (with no correction for having looked across all terms).

Version 1:

Reviewer comments:

Referee #1

(Remarks to the Author)
Generally, the authors have addressed my major concerns. Some technical limitations were anticipated and are understandable. The manuscript has improved substantially during revision and, in my opinion, is suitable for publication in Nature.

I have one minor request.

The authors removed Fig. 2E from the revised manuscript with reasonable justification. However, this change has left the corresponding sections of the Results somewhat difficult to follow.

Specifically, the manuscript first uses MPRA to identify potential cis-regulatory elements (Section 2.1) and subsequently employs human–ape hybrid cells to identify cis-regulated gene expression (Section 2.2).
The chapter concludes by stating that “Combining hybrid cells with MPRAs offers complementary analytical power…” (line 342), followed by “Using this atlas, we studied the genes, pathways, and functions whose regulation diverged in human skeletal evolution.”

This wording suggests that the two datasets were integrated into a unified atlas that was subsequently used for downstream analyses.

However, my interpretation is that this integrated atlas is not actually used in the subsequent analyses. Instead, the

evolutionary changes are analyzed independently using the MPRA dataset (lines 375–397) and the human–ape hybrid dataset (lines 402–420), with the integration occurring only at the level of the overall interpretation (line 421: “Overall, the integration of MPRA and human–ape hybrid data suggests that…”).

I found this transition somewhat confusing and would suggest clarifying how the two datasets are integrated throughout the manuscript. If the analyses are indeed performed largely in parallel, the wording should reflect this more explicitly. Alternatively, if an integrated atlas is central to the downstream analyses, this should be demonstrated more clearly.

(Remarks on code availability)

Referee #2

(Remarks to the Author)
I co-reviewed this manuscript with one of the reviewers who provided the listed reports.

(Remarks on code availability)

Referee #3

(Remarks to the Author)
The authors have thoroughly addressed my comments, amplifying my already high level of enthusiasm for their work.

(Remarks on code availability)
The code underlying the paper now appears to be provided and well organized. I did not attempt to run the code.

Referee #4

(Remarks to the Author)
I feel the authors did a good work in responding to my initial comments, and I have no further comments or concerns. I congratulate the authors on an impressive manuscript.
Craig Lowe

(Remarks on code availability)
I have looked at the organization of the code, but have not gone as far as reproducing results/figures from the manuscript. It does appear that the code is organized with nicely named files and files for similar analyses are grouped together. I think GitHub is a good place to host the code repository. The repository would benefit from having a README file that gives readers an overview of the repository.

Open Access This Peer Review File is licensed under a Creative Commons Attribution 4.0 International License, which

permits use, sharing, adaptation, distribution and reproduction in any medium or format, as long as you give appropriate credit to the original author(s) and the source, provide a link to the Creative Commons license, and indicate if changes were made.
In cases where reviewers are anonymous, credit should be given to 'Anonymous Referee' and the source.
The images or other third party material in this Peer Review File are included in the article’s Creative Commons license, unless indicated otherwise in a credit line to the material. If material is not included in the article’s Creative Commons license and your intended use is not permitted by statutory regulation or exceeds the permitted use, you will need to obtain permission directly from the copyright holder.
To view a copy of this license, visit https://creativecommons.org/licenses/by/4.0/

Comments by Reviewers

Referee #1 (Remarks to the Author):

The study by David Gokhman's laboratory explores the human-specific evolution of genetic regulatory elements in skeletal tissues. The authors utilized highly advanced genomic approaches, including massive parallel reporter assays (MPRAs) and interspecies hybrid iPSC systems. Major findings include predicted evolutionary changes in cartilage glycosaminoglycan (GAG) content and regulation of the Hedgehog signaling pathway, particularly involving the EVC gene. The manuscript provides deep and conceptually novel insights into human evolution and contains a wealth of interesting and valuable data. The experimental tools developed in this work are highly multidisciplinary and broadly applicable.

The manuscript is well written, the data are clearly presented, and most conclusions are appropriately balanced. Overall, this is a nice and ambitious study addressing an important question in human evolution and providing deep insights into the evolution of cis-regulatory elements underlying skeletal divergence between humans and apes.

We thank the reviewer for their thoughtful and encouraging assessment of our work, as well as for their constructive and specific feedback, which we believe substantially improved the manuscript. We also share the reviewer's hope that the experimental approaches developed through the close collaboration between the Inoue and Gokhman labs will prove broadly useful in both evolutionary and non-evolutionary contexts.

I do not have sufficient expertise to critically evaluate all technical aspects of evolutionary genetics or MPRA methodology. Therefore, my comments primarily focus on skeletal aspects of the work (my primary expertise area). Below, I outline my concerns and suggestions for further strengthening the biological interpretation and the manuscript's overall impact.

Major Points (arranged by importance, with the most important first)

The cCRE-based predictions suggest an evolutionarily driven decrease in GAG content in human cartilage relative to apes. However, this proposed reduction may be functionally compensated by additional anchoring sites that evolved within the human aggrecan gene. It is therefore unclear whether this represents an adaptive evolutionary shift, divergence, or fluctuations of unclear nature.

To assess GAG content functionally, the authors compared human samples with those from a single chimpanzee. Given the biological variability of cartilage, this sample size is insufficient to draw robust conclusions.

The authors further argue that mechanical loading reduces proteoglycan and GAG content (refs 79–83) and therefore focus on the thumb, which is not involved in knuckle-walking (lines 542–543). However, mechanical loading has a dual role in cartilage biology. It is widely accepted that physiological loading promotes cartilage health and increases proteoglycan synthesis (PMIDs: 2760736; 8666595; 1787831). In light of this, the similarity in GAG concentrations observed between humans and chimpanzees in the 2nd–4th metacarpophalangeal and interphalangeal joints (Fig. 4g) weakens the interpretation of evolutionary deterioration of human cartilage.

I therefore strongly recommend a more comprehensive comparison of GAG levels across multiple chimpanzees, gorillas, and humans. While I recognize the difficulty of obtaining such samples, specimens from animals that die of natural causes in zoological institutions may be accessible.

We agree with all of these points and introduced the following changes:

(i) Most importantly, we expanded the great ape joint cohort from a single chimpanzee individual to 7 great ape individuals spanning all great ape species (chimpanzee, bonobo, gorilla and orangutan). In addition, we extended the analysis beyond phalangeal joints to include 5 additional joint types, including the elbow (capitulum and trochlea), hip (femoral head), knee (femoral medial condyle and proximal tibia), shoulder and wrist. Altogether, the GAG content analysis now includes a total of 28 great ape joint samples. A summary of these samples now appears in Extended Data Fig. 8-9 and Supplementary Table 21.

(ii) The previous GAG estimation approach normalized samples by tissue weight. However, because articular cartilage mass is composed largely of water, and increased GAG content itself promotes water retention, this normalization introduces a degree of circularity. We therefore implemented an additional, more robust normalization strategy based on DNA content, which is the more common normalization method in comparative analyses of cartilage GAG levels (PMID: 26292283,19464244, 25256035, PMID: 29463990). The manuscript now presents results obtained using both normalization methods.

Together, these results reveal a pronounced and human-specific decrease in GAG content across all examined joints, ranging between 2.6-fold and 4.0-fold. We now describe these results in a new figure (Fig. 4) and in the main text (L586-614).

(iii) We also agree with the reviewer that the previous version did not sufficiently emphasize the dual role of mechanical loading, which can often promote proteoglycan synthesis. Our new results further inform this point: joints that differ substantially in their mechanical loading profiles, including joints inferred to experience higher loading in humans (e.g., the knee ISBN:9781444320039, PMID: 10502242) all exhibit a human-specific reduction in GAG content. This suggests that although differences in mechanical loading likely influence GAG levels, they are unlikely to fully explain the patterns observed here. We now discuss the multifaceted role of mechanical loading and its potential contribution to our findings more explicitly in the main text, and cite the studies suggested by the reviewer (L598-609).

(iv) Finally, we agree with the reviewer that the previous version lacked a sufficiently detailed discussion of whether the observed shift in GAG content is consistent with neutral evolution or adaptation. The evolutionary picture is likely complex, with evidence for both sides. We now discuss the different possible scenarios in the main text: "Whereas great apes show conservation in the GAG pathway at both the genetic and phenotypic levels (Fig. 3,4), the shift in human GAG biosynthesis displays mixed signatures of selection. On one hand, some observations are compatible with relaxation of negative selection, since in the absence of selection, gene expression levels are more likely to decrease than increase(Khaitovich et al., 2005., Kircher et al., 2019), and within-species phenotypic variation tends to increase(Walsh and Lynch, 2018). On the other hand, several other observations are more difficult to reconcile with purely relaxed constraint, and instead point to non-neutral, potentially adaptive changes: (i) the extent of downregulation exceeds neutral expectations derived from saturation mutagenesis experiments (Kircher et al., 2019); (ii) at the protein level, GAG genes show conservation levels comparable to those of other genes (P = 0.635, two-sided t-test on dN/dS values (Durinck et al., 2009), which is inconsistent with relaxed negative selection; and (iii) the differences in GAG content are large and are tightly associated with substantial effects on multiple phenotypes and diseases (Paganini et al.), making a purely neutral explanation less likely. Furthermore, the increase in GAG anchor points in aggrecan could itself reflect either relaxed negative selection or a compensatory adaptive response to reduced GAG levels. Regardless of whether these changes reflect adaptation or relaxation of negative selection, they point to a shift in the selective pressures shaping human skeletal biology, with implications for both skeletal morphology and health." (L880-917).

In addition, differentiation of chimpanzee, gorilla, and human iPSCs into cartilage and direct comparison of their matrix properties would provide additional and strong functional validation of the evolutionary predictions.

A controlled *in vitro* experiment is indeed a powerful complementary approach to studying GAG levels *in vivo*. For such comparisons to be robust and interpretable, the differentiation protocol must be reliably optimized to both human and nonhuman ape cells. Over the past several months, we attempted to adapt several 2D and 3D cartilage differentiation protocols to chimpanzee and gorilla cells. Unfortunately, the gorilla cultures were unstable and highly variable, and despite repeated efforts (including using the extended protocol the reviewer mentioned in point #7), none of the dozens of chimpanzee replicates successfully progressed to the cartilage stage. As observed for other differentiation systems, protocols optimized for human cells proved more difficult to transfer across species than initially anticipated.

Despite these technical limitations, we believe that the extensive in vivo validation across multiple primary joint tissues, individuals, and ape species provides strong support for evolutionary differences in GAG content between humans and nonhuman apes.

The section dedicated to Hedgehog signaling appears disconnected from the main narrative and reads almost as an independent sub-project. Its integration into the overall story should be improved.

One possible solution would be to introduce the Hedgehog pathway earlier, framing the study as a continuation of prior work (ref 42), and positioning cartilage evolution as a downstream consequence of evolutionary modulation of this pathway. Given that Hedgehog signaling is a central regulator of cartilage development and skeletal morphology, structuring the manuscript around this axis could improve narrative coherence.

To make the transition to the *EVC* chapter more integrated into the overall story, while maintaining the funnel-like structure of the manuscript (i.e., moving from genome-wide to pathway-level to specific genes and variants of interest - *EVC*), we added an introductory paragraph to this section, which on one hand, as the reviewer suggested, emphasizes the link between Hedgehog and GAGs, and on the other hand, more clearly flags to the reader that this section represents a different branch of investigation: “In addition to the extensive divergence we observe in GAG biosynthesis, our analyses also point to recurrent evolutionary changes in Hedgehog signaling, a central regulator of skeletal development whose activity is tightly modulated by GAG content (Whalen et al., 2013)” (L669-675).

Although the manuscript is generally well written, several sections appeared mixed between Results and Discussion. Extended interpretative passages (e.g., lines 608–631) would be more appropriate in the Discussion and should be shortened or relocated.

Following the reviewer's suggestion, we reordered several sections to better distinguish between Results and Discussion.

Specifically, the text related to osteoarthritis (previously, lines 608-631) was completely interpretive, and therefore, we moved most of it to the Discussion (L846-879), leaving a summary sentence in the Results: "Interestingly, reduced GAG levels are also linked to several skeletal disorders - all of which are more common in humans than nonhuman apes, even after controlling for age, sex and environmental factors (see Discussion)." (L646-651).

We also shortened the paragraph about the evolutionary history of CSGALNACT1 and ACAN in modern human evolution, which mainly focuses on previous findings (L498-509).

Conversely, parts of the Discussion could be more focused on cartilage biology and Hedgehog signaling. For example, the section discussing the role of GAGs in the immune system (lines 879–896) is not directly relevant to the obtained results and could be replaced or condensed in favor of a discussion of GAG-related skeletal disorders, which are currently addressed in the Results section (lines 608–631). This reorganization would improve thematic consistency.

Following the reviewer's comment, we considerably condensed the non-skeletal section in the discussion, and expanded the section related to GAG-related skeletal disorders (see above).

Minor Comments

1. The abstract states that humans have a greater susceptibility to degenerative joint disease than chimpanzees. Please provide a supporting reference.

We added three references indicating that humans tend to have higher frequencies of degenerative joint diseases compared to other great apes (Jurmain et al., 1989, Jurmain et al., 2000, Poe et al., 2009, and Housman 2021 (L852-864).

2. Line 9: Replace "large brain" with "large skull," as the brain itself is not a skeletal feature.

We have revised the text accordingly, using "braincases" instead of "skulls", as some cranial features became reduced in humans and some great apes have larger skulls overall. We also replaced "a highly divergent cranial shape" with "slender bones" to better reflect subcranial features, which are the primary focus of this manuscript (L2-13, 134-142).

3. Line 86: Replace “the solo resident cell type” with “the major resident cell type” or “the primary functional resident cell type.” Superficial zone cells include chondro-progenitors and are not exclusively mature chondrocytes.

The text was adjusted accordingly (L85).

4. The integration of MPRA and hybrid cell approaches to identify human-specific cis-regulated genes (Fig. 2e; lines 331–338) is conceptually important but is not presented clearly enough. If I interpret the analysis correctly, the authors compare the fractions of up- and downregulated human alleles associated with upregulation of cCRE activity. In this case, the substantial overlap between up- and downregulated groups in the violin plots suggests modest predictive power. Please clarify in the manuscript: (i) the total number of up- and downregulated human-specific cis-regulated genes (out of 4463 identified), (ii) the proportion of each group overlapping with active cCREs, (iii) the interpretation and limitations of this overlap.

We agree with the reviewer that the current analysis demonstrates only modest predictive power. There are several likely reasons why stronger concordance was not observed. First, although related, the two experimental systems are not identical: the MPRA was performed in primary chondrocytes, whereas the hybrid analyses were conducted in osteochondral progenitor cells. Second, differential expression in the hybrid cells reflects the combined effects of many classes of genetic variation, including indels, structural variants, repeat variation, post-transcriptional regulation, and RNA stability, which are not captured by the MPRA design. In addition, enhancer-gene interactions are highly complex and context dependent. Whereas MPRAs measure the intrinsic regulatory potential of isolated sequences, endogenous expression in hybrid cells reflects the integrated output of many regulatory elements acting simultaneously within their native chromatin and 3D genomic context. More broadly, predicting the combined effects of regulatory elements on gene expression remains a major unresolved challenge in genetics and is likely beyond the scope of the current study. Given these limitations, together with the substantial expansion of other sections of the manuscript, we decided that it would be more appropriate to remove this analysis from the current version of the paper.

5. Lines 353–356: Please provide supplementary figures showing enrichment results for each database analyzed. In Figure 3b, enrichment for GAG metabolism is shown, but the legend mentions both GO and HPO datasets. Please clarify whether this is derived from one database or a combined analysis.

Indeed this was unclear. This enrichment analysis was based on three databases: HPO, GO, and GAD disease. Due to space constraints, we presented the top terms from all

three databases in a single figure, while the full results appear in Supplementary Tables 11-14. To clarify the source of each term, we (i) added icons representing the database from which each term originates, (ii) corrected the caption to include GAD, and (iii) expanded the Supplementary Tables to include all, rather than only significant, results (Supplementary Tables 11-14).

6. To complement and strengthen the cCRE-based pathway enrichment analysis (Fig. 3), please perform a similar enrichment analysis for the 4463 human-specific cis-regulated genes identified in hybrid cells and present the enriched pathways.

Following the reviewer's suggestion, we added an enrichment analysis for the 4463 human-specific *cis*-regulatory expression changes identified in the hybrid cells. This analysis further supported our previous results, identifying the glycosaminoglycan metabolism pathway as significantly downregulated (FDR = 2.6×10^{-3}, right-tailed Fisher's Exact).

We also tested enrichment using GO. GAG pathways showed more differential expression than expected by chance: chondroitin sulfate proteoglycan biosynthetic process (2.5x, $P = 5.9 \times 10^{-3}$), glycosaminoglycan metabolic process (1.5x, $P = 0.01$), glycosaminoglycan biosynthetic process (1.5x, $P = 0.03$), glycosaminoglycan catabolic process (1.8x, $P = 0.03$). However these results did not remain significant after multiple-testing correction. We now report this, as well as analyses of HPO and GAD, in Supplementary Tables 15-18 and in L398-424 and L1926-1936.

7. In Figure 4, the authors link human-lineage regulatory divergence to EVC and Hedgehog signaling. However, functional validation of pathway activity is lacking. It would significantly strengthen the manuscript to directly demonstrated changed activity of the Hedgehog pathway. For example, utilizing Prrx1+ iPSC-derived osteoprogenitors and/or mature chondrocytes from human, chimpanzee, and gorilla iPSCs. The differentiation protocol employed by the authors was recently further extended (https://doi.org/10.1101/2025.01.09.631479), which may be particularly helpful for this issue and major issue 1.

We agree with the reviewer that further validation of Hedgehog activity would help clarify the phenotypic consequences of the differential regulatory activity we observe. Experimental induction of Hedgehog signaling in human cells is particularly challenging. Therefore, in collaboration with the Rohatgi lab (Stanford University), which specializes in Hedgehog signaling, we previously attempted to activate this pathway in several human and chimpanzee skeletal cells. Unfortunately, human cells rapidly lose Hedgehog responsiveness in culture and despite repeated efforts, these experiments were unsuccessful. To more clearly acknowledge this limitation, we added the following

sentence to the manuscript: “It remains to be determined how these regulatory changes combine to affect overall Hedgehog signaling activity” (L772-775).

Referee #3 (Remarks to the Author):

This manuscript includes comprehensive experimental evidence that human specific genetic variation has contributed to skeletal evolution in our species. In particular, the authors focus on several data types and analyses:

- First, the authors use MPRAs in human chondrocytes to assay 561,410 SNPs that are fixed in humans in putative regulatory elements. They find that 65,576 of tested fragments have regulatory activity in the query cell type, and that 15,077 loci have evidence for human-specific activity when comparing the derived and ancestral allele. These data point to down regulation of polysaccharide (glycosaminoglycan) biosynthesis genes.
- Second, they generate and assay human-ape hybrid cells (including human-chimp AND human-gorilla) — to identify between species gene expression differences in a common trans-cellular background — and differentiate them into osteochondral progenitors. Specifically, they identify genes in both hybrid cells with between species expression differences indicating regulatory evolution (~4100 chimp specific and ~4400 human specific). These data confirm down regulation of polysaccharide (glycosaminoglycan) biosynthesis genes.
- Third, the authors followed up on the reduction in polysaccharide content in human compared with chimpanzee cartilage using primary samples, finding that in the thumb – where both species experience similar mechanical stress – of a single chimpanzee exhibited 2.3x higher GAG content than 21 humans. They also use loss-of-function phenotypes from human monogenic disorders to predict that downregulation of GAG would result in skeletal changes consistent with those observed between species.
- Finally, the identify genome-wide outliers of differential activity at the EVC locus, and provide several analyses to identify the likely causal SNP as well as the evolutionary history of the locus.

Overall, I found the work to be exciting, comprehensive, and a pleasure to read; the authors provide many different lines of evidence for each conclusion, and the paper addresses an important and understudied topic in human evolution.

We are grateful for the reviewer’s careful evaluation of our study,for the constructive and specific suggestions, and for recognizing the complementary strengths of the MPRA and human-ape hybrid approaches.

However, I do have a few methodological questions that I think are important to dig into to evaluate some of the conclusions. Also, maybe I missed it, but I didn't see a code availability statement for the complete set of analyses performed in this manuscript, and I sometimes found it hard to track down the specific statistical test accompanying a p-value provided in the main text. It would be great to include a GitHub with the processed data matrices and the code used to perform statistical analyses (especially main analyses like differential expression, differential activity, figures, etc). Given that there is a lot going on in this manuscript (because the authors have so many experiments and analyses), seeing the code will be important for making sure all statistical choices about filtering, modeling, multiple hypothesis testing correction, etc are clear and transparent. I appreciate the comprehensive MPRA QC manual, test data sets, and associated scripts, but it is not the same as what I am asking for.

We completely agree with the reviewer that the GitHub repository was incomplete (https://github.com/gokhmanlab/humanMPRA). To enable full reproducibility, we have substantially expanded it to include scripts covering all analyses presented in the manuscript. These include the MPRA pipeline (cCRE–barcode association, activity estimation, and differential activity), the human-ape hybrid pipeline (alignment, count estimation, and differential expression), TF binding analyses, term enrichment analyses, selection scans, figure-generation scripts, now described in the "Code Availability" statement (L1090-1101). The processed data matrices were uploaded to GEO (GSE316891, GSE316892) and as supplementary tables.

In addition, we have uploaded our full MPRA quality control pipeline to GitHub (https://github.com/GokhmanLabOrganization/MPRA_QC_analysis.git). The accompanying manuscript on MPRA best practices is currently under 2nd revision, and we will add a reference to it once it is published.

Finally, we carefully revised the manuscript to more consistently specify the statistical tests associated with each reported *P*-value.

Major comments:

In Fig 1D, it looks like the cCREs only have minimally higher activity than the negative controls. Could you separate the cCREs that you call as 1) having no regulatory activity, 2) having regulatory activity but no allelic differences, and 3) having regulatory activity and allelic differences? Maybe this could be an inset or something to show that the ~18% you call as active hopefully look more like the positive controls. Otherwise, please provide some explanation about these patterns.

Indeed, this is a common behavior in MPRAs that scan cCREs for activity, rather than test previously validated CREs. Because the majority of tested cCREs exhibit little or no

activity, the aggregate signal is dominated by inactive sequences, making the overall effect appear relatively weak. In the revised figure, we now stratify cCREs into active and inactive groups, and the two groups show markedly different activity levels. In addition, following the reviewer's suggestion, we included a new Extended Data Figure (Extended Data Fig. 2i) that further subdivides sequences into those having regulatory activity but no allelic differences, and those having regulatory activity and allelic differences.

The authors asked whether "genes whose human allele is upregulated in the hybrids are enriched for upregulating cCREs in the MPRA, and vice versa." They find that this is weakly true, but they restrict to "genes linked to ≥ 5 differentially active cCREs", which seems like a lot. What happens if this threshold is reduced? And how many genes met this threshold? Some of the weak overlap may also be attributed to chondrocytes and osteochondral progenitors not being the same cell type, so I wonder if you could subset to genes you know are similarly expressed in both cell types. In general, could you also give some intuition or analysis on how similar chondrocytes and osteochondral progenitors are, since inference is linked across the two cell types.

Following this comment and minor comment #4 by Reviewer 1, as well as the substantial expansion of other sections of the manuscript and the fact that this analysis is part of a major unresolved challenge in genetics and is likely beyond the scope of the current study, we decided to remove this paragraph from the paper. For the limitations behind this analysis, please see our response Reviewer 1's minor comment #4.

We agree with the reviewer that part of the weak signal in this analysis was likely attributed to the two experimental systems being similar, but not identical (primary chondrocytes vs osteochondral progenitor cells). To gain insight into the differences between these cells, we followed the reviewer's suggestion and compared gene expression between osteochondral progenitors and chondrocytes. To this end, we used the RNA-seq data from the original Yamada et al. paper (PMID: 34373601) and a published dataset of articular cartilage chondrocytes. The overall correlation is high (Pearson's r = 0.745. $P < 2.23 \times 10^{-308}$), but we agree with the reviewer that the mismatch is partially responsible for the weak predictability of the MPRA of the hybrid data.

The observation that a single chimpanzee exhibited 2.3x higher GAG content than 21 humans is interesting but obviously very limited in having access to only 1 ape biospecimen. How are the authors producing a p-value from 1 observation (I cannot find the analysis strategy in the methods, and only a p-value is provided in the main text)? I think this analysis needs to be reframed as much more exploratory. For example the authors state "our results indicate… reduced GAG content in human relative to chimpanzee joints" but this is really the joint of a single chimpanzee. I also think it's also

important to provide some information about the standard error across the 3 technical replicates, since a lot of inference is resting on the single chimpanzee data point. Additionally, were the human donors age and sex matched to the chimpanzee?

Following this comment, as well as comment #1 by Reviewer 1, we substantially expanded the set of ape joints to include all great ape species (chimpanzee, bonobo, gorilla, and orangutan) and 8 different joint types, comprising a total of 28 ape joint samples.

We also improved the GAG normalization method. The previous analysis normalized samples by tissue weight; however, because articular cartilage mass is composed largely of water, and increased GAG content itself promotes water retention, this normalization introduces a degree of circularity. We therefore implemented an additional, more robust normalization strategy based on DNA content, which is more commonly used in comparative analyses of cartilage GAG levels (see for example PMID: 26292283, 19464244, 25256035, 29463990). The Pearson's correlation between weight and DNA content in our samples is 0.70 ($P = 1.43 \times 10^{-21}$). To test the level of variation between replicates, we examined the correlation between each pair of replicates. We found a high degree of concordance between the three replicates (Extended Data Fig. 10). We also report for each measurement the standard error across the 3 technical replicates (Average 0.49, values ranging between 0.02-5.94) in Supplementary Table 23.

Both the human and ape samples included individuals from both sexes, and their age range partly overlapped: 51-97 (mean: 77+-11.5) for humans, and 20-60 (mean: 30.41+-11.7) for apes, corresponding to 25.6-76.5 (mean: 41.4+-15.1) when corrected for maturation rate differences between humans and nonhuman apes. However, because these factors were not perfectly matched between the groups, we explicitly tested whether they could affect our observed results. First, we examined the correlation between age and GAG content. We found no significant correlation in either humans or apes (Pearson's $r = 0.17$, $P = 0.112$ for humans, and Pearson's $r = -0.20$, $P = 0.440$ for apes). We then repeated this analysis for each joint and reached similar conclusions (P between 0.116 and 0.687 for joints with >=3 samples). We next focused on age- and sex-matched individuals and observed a similar trend to the overall dataset (e.g., a 60-year-old female chimpanzee shows 4.47-fold higher GAG content than the median of her age-matched human female counterparts). Finally, we tested whether males and females differed in their GAG content and found no significant sex effect ($P = 0.740$ for humans, and $P = 0.232$ for apes, two-tailed t-test). These results are now described in the text (L596-598, 2878-2938) and shown in Extended Data Fig. 11.

For joints for which only a single ape individual was available (MCP, PIP, DIP), instead of a *t*-test, we assessed deviation from the human distribution by converting the ape value to a z-score relative to the human mean and standard deviation. A similar two-sided *P*-value was calculated using a non-parametic distribution.

Overall, the updated results reveal a pronounced, human-specific reduction in GAG content across all examined joints, ranging between 2.6-fold and 4.0-fold. We now describe these results in a new figure (FIg. 4) and in L586-814.

The reader needs much more information about the performance of the MPRALegNet model to evaluate its use. What procedures were done to understand model performance? How accurate are the predictions from this model?

We agree with the reviewer that additional information on the performance of the MPRALegNet model (PMID: 39814889) was missing. To assess model performance, we compared the experimentally measured MPRA activity values with the values predicted by MPRALegNet, and added scatter plots of measured versus predicted values, together with Spearman's rank correlation coefficients, as a new Extended Data Fig. 12. Our library contains seven groups of positive and negative controls for activity, as well as the test group. Overall, the model predictions showed a strong correlation for positive controls (Spearman's rho ~0.66-0.85; $P = 2\times10^{-2}$ to 2.5×10^{-115}) and a moderate correlation for negative controls (Spearman's rho ~0.42-0.51; $P = 1.6\times10^{-5}$ to 3.1×10^{-58}) and the test group (Spearman's rho = 0.56; $P < 10^{-300}$), as expected for sequences with lower signal-to-noise levels. We also tested the performance of MPRALegNet in predicting variant effects, and found a moderate correlation for positive controls (Spearman's rho = 0.41; $P < 3.4\times10^{-15}$), and a lower correlation for test sequences (Spearman's rho = 014; $P < 10^{-300}$). This lower correlation was likely attributable, at least in part, to a substantial fraction of false-negative predictions among otherwise correctly predicted positive effects. We now present the performance evaluation of MPRALegNet in the revised manuscript (L712-725), Extended Data Fig. 12, and Methods (L2974-3069).

And then the point of this analysis is just to disentangle whether chr4:5775029 versus chr4:5775100 is the likely SNPs driving between species differences (because these 2 SNPs were assayed on the same fragment)? I was confused why it was necessary to train a predictive model when the region was already evaluated empirically, but I assume this is the goal.

The motivation behind this analysis was missing from the text. Indeed, the purpose of using MPRALegNet was not to replace the empirical evaluation of this region, but rather served two purposes: 1. the *in silico* saturation mutagenesis within the entire empirically assayed fragment helped provide a mechanistic interpretation for the regulatory effect

observed empirically. Specifically, this allowed us to estimate the effects of all possible nucleotide substitutions at every position in the region and thereby identify which bases are predicted to be most important for transcriptional activity. This base-resolution map helped uncover clusters of positions with strong predicted effects, which often correspond to TF binding sites. Indeed, the T→C substitution at chr4:5775100 falls in such a cluster, which also matches several TF binding motifs. 2. The examined fragment contained two human-derived variants. Therefore, based on the empirical data, it was impossible to decipher which of the two variants drove the observed differential activity. The *in silico* model helped point to the variant at chr4:5775100 as the likely driver of the differential activity observed in the MPRA. We revised the main text to reflect these two motivations and to explain our interpretation of the results: “To further examine the potential molecular mechanism underlying this divergence, and to decipher which of the two substitutions is more likely to drive the observed differential activity, we used MPRALegNet (Agarwal et al., 2025, Penzar et al., 2023), which is a deep learning model that predicts regulatory activity from an input sequence” (L698-705).

My biggest concern, which the authors acknowledge, is that the MPRA is only conducted in human cells, and “the trans environment of chondrocytes might have changed since the human-chimpanzee ancestor”. I would like to see the authors convince the reader a bit more that the trans environment is likely similar enough between human and chimp that we should not be concerned about this technical limitation, but only citations to previous work are provided. cell type Are there analyses the authors can include to assuage this concern? For example - what’s the correlation in expression levels between the human chondrocytes and chimp osteochondral progenitors? It would also be useful to know what this looks like for human chondrocytes versus human osteochondral progenitors. Obviously, the best comparison would be RNA-seq from human chondrocytes vs chimp chondrocytes; I’m guessing the authors do not have those data, but any between species comparisons for a relevant cell type — with special attention to the genes and pathways they are trying to highlight — would be helpful.

We agree with the reviewer that the potential impact of trans effects warrants further analysis and clarification. We added the following text to the manuscript: “While the human allele was tested in its native human *trans* environment, the human-chimpanzee ancestral allele was assayed in a human rather than a human-chimpanzee ancestral *trans* environment. For an MPRA sequence to exhibit differential behavior across *trans* environments, the interacting trans factors must differ - either in their protein sequence (affecting binding affinity) or in their expression levels. We therefore sought to assess these sources of potential divergence.

First, we examined evolutionary changes in TF coding sequences that possibly alter DNA-binding specificity. We leveraged the study by King et al. (PMID: 36952575), which identified human-specific variants predicted to affect TF DNA-binding domains. Notably, only three TFs (ZFAT, ZFHX4, and ZNF18) were implicated. Of the three, ZNF18 is not expressed in chondrocytes (TPM < 1). We tested motifs (using FIMO) for the other two TFs within our test sequences and found that none of these TFs is predicted to bind them (FDR > 0.05). This suggests that coding changes in TF DNA binding domains are unlikely to make a major contribution to our results.

Next, we evaluated differences in TF expression levels between humans and chimps. Importantly, human vs chimpanzee divergence overestimates human vs ancestor divergence, as ~50% of human vs chimpanzee changes are chimpanzee-specific and thus, do not contribute to differences between humans and their ancestors. Because expression data from human and chimpanzee chondrocytes are, to our knowledge, unavailable, we analyzed cranial neural crest cells (CNCCs) (PMID: 33731941), which give rise to the facial skeleton. Across TF-encoding genes (N = 736, as defined by JASPAR, PMID: 37962376), we observed high correlations between species (Pearson's r = 0.90, $P = 1.8 \times 10^{-261}$, Spearman's rho = 0.90, $P = 5.2 \times 10^{-272}$), indicating broadly conserved TF abundance across these skeletal contexts.

Because differences in measurements of TF expression between these human and chimp cells are driven not only by genetically-encoded changes, but also by technical factors (e.g., experimental noise), we turned to investigate TF expression in the human-ape hybrid osteochondral progenitors, which provide a more tightly controlled system for assessing genetically-encoded *cis*-regulatory differences affecting TF genes. We found that TF-encoding genes show a high correlation in these cells as well (Pearson's $r = 0.94$, $P = 1.1 \times 10^{-276}$).

Finally, as the reviewer suggested, we also compared gene expression profiles between human chondrocytes and human osteochondral progenitors (Yamada et al., PMID: PMID: 34373601), and observed high overall concordance (Pearson's r = 0.86, $P < 2.2 \times 10^{-308}$), further supporting the notion that closely related skeletal cell types share a similar trans environment.

While these results suggest that most TFs likely have similar levels in human and human-chimp ancestor cells, there are nevertheless some differences. Prior works suggest that such variation primarily affects the magnitude rather than the direction of allelic effects. For example, in our MPRA of modern human-derived variants in osteoblasts and neural progenitor cells, all allelic pairs (100%) showed concordant directionality of effect across the two cellular contexts (PMID: 33885362). To further examine this, we analyzed our positive control sequences, previously assayed in

osteoblasts and here in chondrocytes, and found that 89.52% retained the same direction of differential activity across cell types, and also exhibited a strong correlation in effect size (Pearson's r = 0.64, $P = 2.2 \times 10^{-23}$).

Overall, while the precise contribution of trans differences between a human cell and a human-chimp ancestor cell to differential activity is difficult to quantify, our analyses suggest that this contribution is likely limited, particularly with respect to the directionality of allelic effects." (L950-956, L1976-2077).

The authors provided some analyses to attempt to link the between species datasets to within human variation and human diseases (e.g., osteoarthritis), but I felt like this could have gone further. If the argument is that genes with cCREs with significant between species differences are important for skeletal traits, I would also expect this to be true within species when variation occurs in these regions. For example, are genes with cCREs with significant between species differences enriched for GWAS associations with human skeletal diseases or traits? Or is genetic variation within humans that occurs in these cCREs more likely to generate eQTL? Analyses along these lines would be helpful for understanding the likely relevance of these regions for human disease.

We agree with the reviewer that these cCREs are expected to be associated with within-species skeletal functions and phenotypes. This association is expected to be observed at both the cCRE- and target gene levels. To test this, we compiled a set of GWAS variants associated with skeletal traits by filtering the GWAS Catalog for phenotypes containing skeletal terms (see Methods). We then defined 1-kb windows centered on these variants and tested whether human-derived variants in differentially active cCREs were enriched in these regions relative to other variants in our MPRA library. Consistent with our expectation, human-derived variants in differentially active cCREs showed a significant enrichment within skeletal GWAS regions (Fisher's exact test, P = 0.02). We now present this result in L2078-2124.

We are not aware of a robust eQTL dataset for cartilage or bone, either in GTEx or in other sources. Nevertheless, several other findings in our study suggest an association between differentially active cCREs and loci/genes affecting skeletal variation within humans. These include (i) the enrichment of several skeletal terms among the top 20 enriched terms: *skeletal morphology* (GO), *campodactyly* (HPO), *Clindodactyly of the 5th finger* (HPO), *Body height* (GAD), and *Hip* (GAD, Fig. 3), and (ii) elevated MPRA activity within chondrocyte open chromatin regions (Fig. 1e).

Minor comments:

To identify human specific fixed alleles, the authors compared ape genomes to gnomAD. Are these variants also fixed in 1000 Genomes and HGDP, which include a

more global and diverse set of whole genome sequences? For example, it would be helpful to check the variants here: https://pmc.ncbi.nlm.nih.gov/articles/PMC11216312/

Following the reviewer's comment, we evaluated the allele frequencies of our assayed variants in additional datasets. The original library is based on gnomAD version 2.1, which does not include the HGDP and 1000 Genomes datasets. We therefore analyzed gnomAD version 4.0, which contains sequencing data from 730,947 individuals, including the individuals from the 1000 Genomes and HGDP datasets. In this dataset, 99.96% of our variants had a frequency above 0.999 (and 81% are completely fixed).

We also examined a combined dataset of 1KG+HGDP (downloaded from gnomAD version 3.1). In this dataset, 99.91% of our variants had a frequency above 0.999 (and 96.8% are completely fixed).

Together, these analyses indicate that the vast majority of variants in this library are either fixed or nearly fixed in humans. We added the frequency information above to the Methods (L1177-1190) and revised the text accordingly: *"...we assayed 561,410 fixed **or nearly fixed** human-derived single-nucleotide substitutions"* (abstract) and throughout the paper (L73-75, and Fig. 1 caption).

To narrow down the list of human specific variants to include in the MPRA, the authors overlapped the candidate SNP list with putative regulatory elements from SCREEN v2, which draws on active chromatin marks across 839 human cell types and tissues. The vast majority of these 839 human cell types are not relevant to the skeleton. Why did the authors choose to use this broad approach, rather than subsetting to putative regulatory elements in cell types of interest?

There were three main reasons for this choice:
(i) Transportability: to enable follow-up MPRAs in additional cell types, we designed a library that can be assayed across diverse cellular contexts, supporting robust cross-cell-type comparisons.
(ii) Data availability: at the time of library design, SCREEN annotations for chondrocytes were not yet available.
(iii) Generalizability: even if such annotations had been available, accessibility can differ across experiments and chondrocyte cell lines. To maximize robustness across current and future chondrocyte maps, we favored a broader selection of regions rather restrictive pre-filtering, as more specific filtering can be applied post hoc.

To characterize chondrocyte chromatin landscape and enable cell-type-specific filtering, we performed ATAC-seq and H3K27ac Cut&Tag on the primary chondrocytes used in the MPRA (data deposited in DDBJ under accession number PRJDB40123). This

allowed us to filter for chondrocyte-relevant cCREs where needed (e.g., Fig. 1e and Fig. 4a).

We now mention the ATAC-seq and H3K27ac Cut&Tag data in the main text of the results: "*Lastly, we performed ATAC-seq and H3K27ac Cut&Tag on these cells to identify endogenously accessible cCREs (see Methods)*" (L171-174). In addition, we added to Supplementary Table 4 the distance of each cCRE to chondrocyte ATAC-seq and H3K27ac peaks.

The authors state that there is "higher activity of cCREs that (i) overlap open chromatin in chondrocytes ($P = 4.9 \times 10^{-324}$, Kolmogorov-Smirnov test), or (ii) overlap promoter marks ($P = 1.3 \times 10^{-307}$, Kolmogorov-Smirnov test)." Is this enrichment higher than if they use annotations from other cell types? In other words, how specific is the enrichment/signal to chondrocytes?

To assess this, we compared the correlation between cCRE activity and chromatin accessibility in chondrocytes and 12 additional cell types. This analysis was performed twice: first across all cCREs, and then focusing on putative enhancers, which are more cell type-specific than promoters. We found that chondrocytes exhibit the strongest correlation between chromatin accessibility and MPRA activity for enhancers (Spearman's rho = 0.25), and the second strongest when considering all cCREs (Spearman's rho = 0.34, now presented in Extended Data Fig. 3). We updated the Methods and main text accordingly (L217-225 and L1954-1975).

Could you add an x=y line to Fig 1c?

Done.

The authors state that they found ~4100 chimp specific and ~4400 human specific cis regulatory expression changes. What about gorilla specific expression differences?

We identified 3,766 genes whose expression differs in gorilla relative to both humans and chimpanzees. Notably, these genes are not necessarily gorilla-derived, as some of the observed differences may instead reflect expression changes that emerged in the common ancestor of humans and chimpanzees. This number is slightly lower than the number of human- and chimp-derived changes, likely reflecting reduced statistical power due to the smaller number of human-gorilla hybrids (n = 2). We now present these data in Supplementary Table 8.

I was surprised to not see any general tests for TF binding enrichment analyses for the differentially expressed cCREs. Are these sequences enriched for any known TF motifs?

Indeed, an earlier version of the manuscript included a section on TF binding, which we ultimately removed to reduce overall complexity. However, we agree with the reviewer that this mechanistic insight is important. We have therefore reintroduced this analysis into the main text: “To gain insight into the mechanisms underlying differential activity, we analyzed transcription factor (TF) binding using two approaches: (i) sequence matching to previously reported TF binding motifs (Grant et al., 2011), and (ii) estimation of binding affinities using protein binding microarrays (Berger et al., 2006). We identified 20 TFs that are both enriched within differentially active sequences, and whose inferred differential binding levels are correlated with differential MPRA activity (Extended Data Fig. 4, Supplementary Tables 5-7), see Methods). These findings suggest that these 20 TFs may have contributed more prominently to divergence in regulatory activity” (L253-269).

The total pool of candidate sequences were divided across 9 sub libraries to accommodate technical constraints. Were any sequences repeated across sub libraries to understand batch effects? Were there any batch effects on activity levels?

All sublibraries share a large set of positive and negative controls (n=150 and n=300, respectively) to help identify potential batch effects (see *Library Design* section in the Methods). Following the reviewer’s comment, we added Extended Data Fig. 2h, which shows the correlations in positive control activity across all sublibrary pairs. Most sublibraries exhibit high correlations with one another (Pearson’s r = 0.69-0.97), with the exception of the pilot sublibrary (L1a1) and the smallest sublibrary (L4a1), which show moderate correlations (Pearson’s r = 0.29-0.83). Most importantly, sequences were randomly distributed across libraries, and both alleles of the same sequence were always included in the same sublibrary to avoid batch effects between allelic pairs.

The authors state that “Some cCREs with multiple barcodes had one or more barcodes with extreme RNA read counts compared to the other barcodes of the same cCRE allele, while not having higher DNA counts. Such outliers are likely attributed to hyperactive integration loci, and do not represent the true endogenous activity of the cCRE.” Can you provide some information on how often this lack of replication happens?

This filtering step indeed required additional clarification. On average, 3.58% of barcodes were removed per replicate (324,822 in total; 4.39 per cCRE). We now include this information in L1526-1528.

The authors use both MAD score and RNA/DNA ratio as a measure of activity. Could you provide some information about how correlated these are? They seem to be used interchangeably.

Indeed, two complementary measures of activity are used in the manuscript, each serving a slightly different purpose. While the two are correlated (Spearman's rho = 0.613), the MAD score is the preferred measure, as it is derived from a statistical model that controls for batch effects, replicate variation, barcode number, and additional sources of noise. Accordingly, we use this metric for the majority of analyses. In replicate-specific and barcode-specific analyses, where this statistical model is inapplicable, we use the simpler RNA/DNA ratio. We have added Extended Data Fig. 2a to illustrate the relationship between the two.

In methods section 6.3.3, could you provide some more information on what's going on with seq325030?

Seq325030 contained the highest number of barcodes (10,933) among all sequences in sublibrary L3a2, exceeding the capacity of the statistical model of MPRAnalyze. To address this, we randomly subsampled ~half of the barcodes (5,486), allowing the model to run while still retaining substantially higher BC coverage than the sublibrary average (203 barcodes per cCRE). We now explain this in the Methods (L1583-1585).

In methods section 6.9.1, did a gene need to be differentially expressed in both chimp-human and gorilla-human comparisons? Or just significantly differentially expressed in at least one comparison, with the other comparison used to polarize?

We required a gene to be differentially expressed only between human-chimp, with the human-gorilla data used for polarization. We clarified this in the text: "For this purpose, we did not require the human-gorilla difference to be significant" (L2532-2534).

In extended data figure 2, why are there a large number of cCREs that have RNA/DNA >> 0 but are not called active? The correlation between replicates generally looks good, so I am surprised if there is a large class of elements with high enough standard errors that they are not reaching significance thresholds.

The statistical framework used to determine the activity state of each oligo (MAD score) accounts for multiple factors beyond the RNA/DNA ratio, including read depth, and variation of DNA and RNA across replicates. This is critical, as cCREs with low read counts may exhibit inflated RNA/DNA ratios but lack sufficient statistical support to be classified as active. For example, seq_350395 shows 0.758 RNA CPM and 0.349 DNA CPM, yielding a relatively high RNA/DNA ratio of 2.2; however, due to limited DNA read depth and low number of barcodes (N= 82 and N=25, respectively), the model does not classify it as significantly active (P = 0.28, MAD = 0.57).

Referee #4 (Remarks to the Author):

Yan et al. present an in-depth analysis of cis-regulatory divergence on the human lineage with respect to chondrocytes.

Overview:

The authors use a large-scale MPRA in human chondrocytes to test over 300,000 regulatory regions containing more than 500,000 DNA substitutions that are fixed in humans. This provides an overview of cis-regulatory divergence in human chondrocytes at the DNA level, due to substitution mutations. Additionally, the authors use human-chimpanzee and human-gorilla cell fusions to create allotetraploid cells that they differentiated into chondrocytes. They then compare the expression levels of the human and non-human alleles in these allotetraploid chondrocytes, which allows them to polarize human-chimp gene expression differences into changes on the human or chimp branches. This analysis provides an overview of cis-regulatory divergence in human chondrocytes at the RNA level. In both of these analyses, the authors note the cis-regulatory downregulation of the GAG biosynthesis pathway in humans, compared to the other great apes. Consistent with this observation, the authors also see reduced GAG content in human joints compared to chimpanzees. To understand additional phenotypic changes that could be associated with this cis-regulatory downregulation, the authors use phenotypes from human disorders linked to this pathway to propose that human-specific skeletal changes and osteoarthritis risk are likely impacted by these cis-regulatory changes.

This is an impressive body of work that will be useful to the scientific community both as a resource and for its biological discoveries. While I have some comments on the manuscript (see below), the comments do not dampen my enthusiasm for this work.

We highly appreciate the reviewer's constructive comments, which we found very helpful in refining the manuscript. We are also pleased that the reviewer found both the resource and the biological insights valuable.

Comments:

1) The breadth of the MPRA experiment is impressive, but it was difficult to understand what percent of the human genome could be assessed for fixed human differences, and what percentage of the genome may still hold fixed alleles that have not been tested yet. It would be helpful to know the percentage of the genome where the great ape allele could be assessed (i.e. passing the read depth filters, more than 10bp away from an indel, etc), the human allele could be confidently called across all human samples (it

was a bit unclear if the authors only required a lack of a variant call, or if all samples needed a confident call of matching the human reference assembly for the gnomAD samples), and avoided the problematic tracks from UCSC and ENCODE. I don't disagree with the filters that the authors applied in order to have a high-confidence set, but I was left wondering how much of the genome was not included due to these conservative filters.

This is an important point, and we thank the reviewer for raising this oversight. Overall, 84.41% of genomic positions in SCREEN cCREs passed our quality filters. We added the following text to the Methods section (L1274-1284):

"In total, among genomic positions annotated as cCREs by SCREEN V2 (Moore et al. 2020, which represent 7.62% of the genome, 13.98% did not pass genotype quality control, 1.90% overlapped repetitive or ENCODE's set of "problematic" regions, and 2.54% were located near indels. After accounting for overlaps between these regions, 15.59% of the cCRE genomic territory was excluded. The remaining 84.41% was retained for identification of human-derived substitutions."

Regarding our approach to filtering out positions that are not fixed in the human population: our approach ascertains that no other individual in gnomAD shows the alternative (ancestral) allele. However, we did not require a minimum number of genotyped individuals in gnomAD, leaving open the possibility that the absence of the alternative allele reflects missing data rather than true fixation. To test this, we reanalyzed the gnomAD data and found that 99.9963% of positions in our catalog have at least 1,000 genotyped individuals in gnomAD (coverage >5 reads). We now report this in the Methods: "Overall, 99.9963% of positions had at least 1,000 genotyped individuals (read coverage > 5)" (L1143-1146).

2) The main text says there were 1,527 positive controls, 3,436 negative controls, and 1,329 sequence pairs as controls for differential expression. In the methods section "MPRA controls," I only found 38 + 12 positive controls, 100 + 100 + 100 negative controls, and 407 * 2 controls for differential expression. I couldn't figure out how to make the numbers in the main text and the methods match up.

We thank the reviewer for pointing this out. This part was unclear for two reasons: (i) We accidentally included in this description 27 deprecated control sequences that belonged to a different experiment. (ii) The description in the main text included the sum of control sequences across sublibraries, whereas the Methods described the numbers per sublibrary. For consistency, we updated the main text to reflect the total number of control sequences: "*In addition, we included 1,500 positive, and 3,000 negative controls for activity, as well as 1,307 sequence pairs as positive controls for differential activity.*"

(L163-167). Finally, we added Supplementary Table 1, which details all the controls used in this study.

3) The MPRA library was filtered for being in regulatory elements (i.e., SCREEN), but I believe this regulatory signal could have come from tissues/cells unrelated to chondrocytes. The main text does say that the MPRA-positive fragments in the library tend to overlap open chromatin, but I am curious about the exact numbers of MPRA-positive/negative elements that do/don't overlap open chromatin. Would MPRA-positive fragments in closed chromatin be considered false positives (in the context of the MPRA experiment)?

As the reviewer describes, the MPRA library was designed based on active chromatin regions across many cell types. There were three main reasons for this choice:
(i) Transportability: to enable follow-up MPRAs in additional cell types, we designed a library that can be assayed across diverse cellular contexts, supporting robust cross-cell-type comparisons.

(ii) Data availability: at the time of library design, SCREEN annotations for chondrocytes were not yet available.

(iii) Generalizability: even if such annotations had been available, accessibility can differ across experiments and chondrocyte cell lines. To maximize robustness across current and future chondrocyte maps, we favored a broader selection of regions rather restrictive pre-filtering, as more specific filtering can be applied post hoc.

More specifically, we do not classify active MPRA sequences lacking active chromatin marks as false positives. This is because (i) the set of chromatin marks used by SCREEN is relatively limited (e.g., a single histone modification mark - H3K27ac - for putative enhancers), (ii) some bona fide regulatory elements are known to lack canonical chromatin marks (e.g., PMID: 40775209), and (iii) the presence of active chromatin marks in other cell types suggests that these sequences may have regulatory potential outside of chondrocytes. Accordingly, while we consider cCREs that exhibit both MPRA activity and active chromatin marks in chondrocytes as higher-confidence cCREs, we do not consider active cCREs lacking such marks as false positives. Instead, for analyses requiring greater stringency (e.g., prioritization of top variants, such as in *EVC*), we prioritize variants supported by both MPRA activity and chromatin evidence (including the ATAC-seq and H3K27ac maps that we generated from the chondrocytes used in the MPRA).

As the reviewer mentioned, Fig 1e shows higher activity for cCREs located in open chromatin, but does not provide exact numbers. To address this, we overlapped our

chondrocyte ATAC-seq and H3K27Ac data with active and non-active cCREs, as described below:

	Closed chromatin (no ATAC-seq peak or H3K27Ac)	Open chromatin (ATAC-seq peak or H3K27Ac)
Active cCREs (MPRA)	23,374	28,658
Non-active cCREs (MPRA)	177,129	84,197

Applying Fisher's exact test on this contingency table, we found that active cCREs are ~3-fold more likely to reside in open vs closed chromatin ($P < 10^{-308}$). This is also in line with our previous MPRA on modern human-derived variants (Weiss et al., 2021). We adjusted the text in L217-225 to add this information.

4) The cell fusions are an elegant and powerful system for studying cis-regulatory changes. When the cell fusions between humans and other great apes are first introduced, I felt that the description was too brief; there was a mention of more details about the tradeoffs of this system in the discussion, but I did not find this in the discussion. For example, the text states "... fully tetraploid hybrids of closely related species ... maintain the balanced gene dosage of diploid cells ..." This is largely true, but one of the studies cited to support this claim (PMID: 34921118) shows that about 180 genes change their regulation by more than 2 fold when comparing standard human/chimp diploid cells with autotetraploid cells. Additionally, tetraploid human pregnancies rarely go to term and are associated with skeletal changes (PMID: 14632567), which hints at gene regulatory differences. I think it would benefit the reader to have a more thorough description of the strengths and limitations of the system.

We agree with the reviewer that this section needed further elaboration on the tradeoffs of this system. We therefore toned down the claim about dosage stability to: "fully tetraploid hybrids of closely related species ... **largely** maintain the balanced gene dosage of diploid cells…" (L298-301). We also added a passage in the Discussion about the limitations of the hybrid system: "while tetraploid cells largely maintain the balanced gene dosage of diploid cells, some genes exhibit ploidy-depenedent effects (Song et al., 2021). Moreover, although tetraploid cells can be stably differentiated to a wide range of cell types (Song et al. 2021, Agoglia et al. 2021, Gokhman et al. 2021, Wang et al. 2024, Song et al. 2025, Carter el al. 2025, Schmitz et al. 2025, Ovrebo et al. 2018), tetraploid human pregnancies rarely go to term and are associated with developmental abnormalities, including skeletal defects (Nakamura et al. 2003). In this context, integrating data results with MPRA results, which are not subject to

ploidy-related effects, helps provide orthogonal evidence for regulatory divergence." (L966-979). We also expanded the main text to clarify how these cells are generated (fusion rather than sexual reproduction (L282-285), see next comment).

5) The authors use phrases like "human-ape hybrid" to describe the cells, which has both pros and cons. The term hybrid is broadly accessible, which is helpful, but also evokes the idea of reproduction between the species. "Allotetraploid" is a more precise description of the cells, although it is a less well known term. It may be beneficial to incorporate some suggestions from the paper, PMID: 35082442, in the revision.

We agree that "allotetraploid" is a more precise term, whereas "hybrid" is more accessible. To balance between the pros and cons of each term, we made the following revisions to the text:
(i) As suggested by the reviewer and in PMID: 35082442, when we first introduce these cells in the introduction and main text we use "composite cell" and "allotetraploid composite cell", and then clarify for non-specialist readers that these cells are commonly known as interspecies hybrid cells: "*using allotetraploid composite cell lines, commonly known as interspecies hybrid cells*" (L280-282).
(ii) Following the recommendations in PMID: 35082442, we also explicitly note that "*These cell lines are generated via interspecies cell fusion, rather than sexual reproduction*" (L282-285) to alleviate ethical and legislative concerns.
(iii) Throughout the Methods, we now use "composite cells".
(iv) Given the broad readership of Nature, we prioritize accessibility in the main text by primarily using "hybrid cells," while noting "composite cells" or "allotetraploid composite cells" in parentheses at key points (L280-281, L315).

6) I am wondering if the p-values related to the lineage specific selection test need to be corrected for multiple tests. It was unclear to me from the main text and methods section if the p-values were corrected or not. I am thinking that there should be a correction because the main text reads that a number of gene annotation databases (i.e., IPA, GO, HPA, GAD) were assembled and then the authors searched across the annotations for terms with many affected genes, including which terms had the most down-regulation associated with them. Example, "Similarly, we found that in IPA, the most significantly downregulated process was glycosaminoglycan metabolism." This reads to me that the authors searched across many terms to identify those with the most downregulation, but then only applied the lineage-specific statistical test to the most extreme term that they identified (with no correction for having looked across all terms).

This part was indeed unclear and required several adjustments. Importantly, all P-values from the enrichment tests on the IPA, GO, HPO and GAD databases were already corrected for multiple testing. These analyses revealed GAG biosynthesis as the most enriched term genome-wide. Notably, unlike the rest of the databases above, IPA also tests the directionality of changes by integrating prior knowledge of each protein's functional role within the pathway (activator or repressor) with the observed direction (up or down), but not magnitude, of regulatory change. The IPA analysis, together with the additional results described in this section, pointed to a likely downregulation of this pathway. Based on these findings, we next focused on GAG biosynthesis as our primary target of investigation.

We then performed a more detailed investigation of this pathway by testing potential selective forces shaping its regulation. To this end, we applied a simulation-based lineage-specific selection test that integrates the distribution of variant effects sizes with genomic architecture (cCRE-gene links) to identify deviations from neutral evolution. To increase resolution within GAG biosynthesis, we partitioned it into its four constituent pathways using the KEGG database, and applied the test to each of these four pathways.

We acknowledge that these four tests should have been corrected for multiple comparisons, and we have now implemented this correction. After adjustment, the three previously significant GAG pathways remain significant (FDR = 0.01 for all three; L443-451).

We have also revised the main text to clarify the permutation-based test for lineage-specific selection: "*This test integrates the architecture of cCRE-gene links with the number and magnitude of up- and downregulatory effects to estimate whether their cumulative impact leads to more extreme regulatory shifts than expected under a random model (see Methods)*" (L430-436).

Finally, we confirmed that all other statistical tests in the manuscript have been appropriately corrected for multiple testing, and no additional changes were required.
