# Supplementary material for: The gene-regulatory evolution of the human skeleton
Source: Nature. 2026 Sep 23;658(8136):698–706. doi: 10.1038/s41586-026-11053-x (PMC13668659; doi:10.1038/s41586-026-11053-x)
Supplement: Supplementary file 3 [file 41586_2026_11053_MOESM1_ESM.docx]

Supplementary Information

# Supplementary Results

## MPRA validations

We validated the MPRA results and ensured it captured true regulatory activity by following the steps described in our MPRA quality control guidelines^23^. We tested the following key metrics: (i) DNA complexity, (ii) RNA complexity, (iii) reproducibility, (iv) dynamic range of activity, and (v) concordance with endogenous signals. We found that the experiment showed high scores across all five metrics (**Fig. 1**, **Extended Data Fig. 2**), including a high correlation between replicates (**Fig. 1b**), a wide dynamic range of activity (**Fig. 1c**, **Extended Data Fig. 2b**), a clear separation between positive and negative controls (**Fig. 1d**), and a high concordance with endogenous genomic signals (**Fig. 1e**), including higher activity of cCREs that (i) overlap open chromatin in chondrocytes (*P* = $4.9\times{10}^{-324}$, Kolmogorov-Smirnov test), or (ii) overlap promoter marks (*P* = $1.3\times{10}^{-307}$, Kolmogorov-Smirnov test). In line with the increasing importance of an enhancer’s intrinsic strength the further away it is positioned from its target gene^146^, we found that cCREs overlapping putative distal enhancers showed higher activity than proximal enhancers (*P* = $2.0\times{10}^{-72}$, Kolmogorov-Smirnov test, **Fig. 1e**). Active cCREs were threefold more likely to be located in active open chromatin than non-active cCREs, and their activity correlated most strongly with the level of open chromatin in chondrocytes compared to other cell types (Spearman’s rho = 0.25, *P* = $2.4\times{10}^{-44}$ for enhancer-like elements, **Extended Data Fig.** 3, **Supplementary Table** 3, see Methods). Overall, these results suggest that the MPRA successfully captured endogenous regulatory activity in chondrocytes across a wide range of activity and in a reproducible way.

## Transcription factor binding

To gain insight into the mechanisms underlying differential activity, we analyzed transcription factor (TF) binding using two approaches: (i) sequence matching to previously reported TF binding motifs^40^, and (ii) estimation of binding affinities using protein binding microarrays (PBMs)^147^. We identified 20 TFs that are both enriched within differentially active sequences, and whose inferred differential binding levels are correlated with differential MPRA activity (**Extended Data Fig. 4**, **Supplementary Tables 5-7**, see Methods). These findings suggest that these 20 TFs may have contributed more prominently to divergence in regulatory activity.

## Phenotypes associated with GAG reduction

Reductions in GAG biosynthesis affect many morphological phenotypes, primarily skeletal, including short stature, facial flattening, high forehead, short fingers, thumb reorientation, and more^34^. Changes in GAGs have also been implicated in shaping phenotypes across diverse species^52^. To assess the potential consequences of GAG downregulation, we applied our directional phenotyping framework^25,53–55^, which leverages loss-of-function phenotypes from human monogenic disorders to infer the expected direction of phenotypic change under reduced gene activity and tests their concordance with interspecies differences. Applied to GAG biosynthesis genes, this analysis revealed that phenotypes driven by reduced GAG biosynthesis match particularly well with human-specific phenotypes, with chondroitin sulfate biosynthesis showing the strongest signal (2.9x more matches than mismatches; *P* = $9.8\times{10}^{-3}$, *t*-test; **Extended Data Fig. 7b**, **Supplementary Table 18**). For example, reduced chondroitin sulfate biosynthesis results in traits such as short feet, broad forehead, flat face, lumbar spine hyperlordosis, narrow chest, small teeth, and short palms^34^ - all match the direction of human-specific traits^1^. Interestingly, reduced GAG levels are also linked to several skeletal disorders, each more common in humans than nonhuman apes, even after controlling for age, sex and environmental factors (see Discussion). Together, these observations suggest that the human-specific reduction in GAG content likely contributed to both skeletal morphology and to the increased susceptibility of humans to degenerative skeletal diseases.

# Supplementary Discussion

## Strengths and limitations of MPRAs and composite cells

MPRAs and interspecific hybrids currently represent the gold standard for measuring *cis*-regulatory divergence at scale, and have been shown to replicate sequence and gene activity in their endogenous context^24,25,62,148,149^. However, both approaches have limitations that can primarily generate false negatives. First, owing to oligonucleotide synthesis constraints, our MPRA library was limited to 270-bp inserts, potentially missing regulatory activity encoded in longer sequences^148^. Second, while interspecific hybrids capture *cis*-regulatory divergence arising from any underlying mechanism, including indels and structural variants, MPRAs do not. Third, because MPRA sequences integrate randomly into the genome, elements that depend on their native genomic context may show altered activity. Such effects should similarly influence the human and ape alleles^148^, but we further mitigate them by using antirepressors, multiple independent integrations, and filtering for endogenous active chromatin marks. Fourth, differences in cellular *trans* environment could influence *cis*-regulatory activity. However, previous MPRAs performed in both human and chimpanzee cells^150^, and across more divergent organisms^151^, demonstrate high conservation. We further validate this by examining human-derived amino acid changes in TFs, and comparing expression levels of TFs between humans and chimps and across skeletal cell types, indicating similar *trans* environments (see Methods). This suggests that the *trans* environments of the same cell type in two closely related organisms tend to affect *cis*-regulation similarly. Fifth, our library used strict 100% fixation criteria. However, as additional archaic and modern genomes become available, in particular from underrepresented populations^152^, future studies should also consider low-frequency^152^, and recurrent mutations across primates^15^. Sixth, while tetraploid cells largely maintain the balanced gene dosage of diploid cells, and can be stably differentiated to a wide range of cell types^24–30,153^, some genes exhibit ploidy-depenedent effects^26^, and tetraploid human pregnancies rarely go to term and are associated with developmental abnormalities, including skeletal defects^154^. Integrating hybrid with MPRA data, which are not subject to ploidy-related effects, helps provide orthogonal evidence for regulatory divergence. Finally, divergence in repressors is less likely to be detected in MPRAs due to the low basal activity of the minimal promoter used.

# Supplementary Figures

**Supplementary Fig. 1.** **Representative flow-cytometry gating strategy**. The main cell population was selected by FSC-A and SSC-A gating, followed by sequential exclusion of doublets using FSC-A/FSC-W and SSC-A/SSC-W. Viable cells were identified as DAPI-negative in the BV421 channel. The resulting viable singlet population was then analyzed for GFP and APC fluorescence, and the P5 gate was used to quantify the double-positive population.

# Supplementary Information References

146.	Wu Y, Li J, Bartley-Dier EL, Pitts C, Cohen BA. Long-range massively parallel reporter assay reveals rules of distal enhancer-promoter interactions [Internet]. bioRxiv; 2025 [cited 2025 Nov 1]. Available from: <https://www.biorxiv.org/content/10.1101/2025.04.21.649048v1> doi:[10.1101/2025.04.21.649048](https://doi.org/10.1101/2025.04.21.649048)

147.	Berger MF, Philippakis AA, Qureshi AM, He FS, Estep PW, Bulyk ML. Compact, universal DNA microarrays to comprehensively determine transcription-factor binding site specificities. Nature Biotechnology. 2006 Nov;24(11):1429–35. doi:[10.1038/nbt1246](https://doi.org/10.1038/nbt1246)

148.	F I, M K, B M, Gm C, Dm W, Mt M, et al. A systematic comparison reveals substantial differences in chromosomal versus episomal encoding of enhancer activity. Genome research. 2017 Jan;27(1). doi:[10.1101/gr.212092.116](https://doi.org/10.1101/gr.212092.116)

149.	Klein JC, Agarwal V, Inoue F, Keith A, Martin B, Kircher M, et al. A systematic evaluation of the design and context dependencies of massively parallel reporter assays. Nature Methods. 2020 Nov;17(11):1083–91. doi:[10.1038/s41592-020-0965-y](https://doi.org/10.1038/s41592-020-0965-y)

150.	Whalen S, Inoue F, Ryu H, Fairr T, Markenscoff-Papadimitriou E, Keough K, et al. Machine learning dissection of Human Accelerated Regions in primate neurodevelopment. Neuron. 2023 Mar;111(6):857–873.e8. doi:[10.1016/j.neuron.2022.12.026](https://doi.org/10.1016/j.neuron.2022.12.026)

151.	Mattioli K, Oliveros W, Gerhardinger C, Andergassen D, Maass PG, Rinn JL, et al. Cis and trans effects differentially contribute to the evolution of promoters and enhancers. Genome Biology. 2020 Aug;21(1):210. doi:[10.1186/s13059-020-02110-3](https://doi.org/10.1186/s13059-020-02110-3)

152.	Kuhlwilm M, Boeckx C. A catalog of single nucleotide changes distinguishing modern humans from archaic hominins. Scientific Reports. 2019 Jun;9(1):8463. doi:[10.1038/s41598-019-44877-x](https://doi.org/10.1038/s41598-019-44877-x)

153.	Øvrebø JI, Edgar BA. Polyploidy in tissue homeostasis and regeneration. Development (Cambridge, England). 2018 Jul;145(14):dev156034. doi:[10.1242/dev.156034](https://doi.org/10.1242/dev.156034)

154.	Nakamura Y, Takaira M, Sato E, Kawano K, Miyoshi O, Niikawa N. A tetraploid liveborn neonate: Cytogenetic and autopsy findings. Archives of Pathology & Laboratory Medicine. 2003 Dec;127(12):1612–4. doi:[10.5858/2003-127-1612-ATLNCA](https://doi.org/10.5858/2003-127-1612-ATLNCA)
